# Antimicrobial resistance of *Streptococcus pneumoniae* from invasive pneumococcal diseases in Latin American countries: a systematic review and meta-analysis

**DOI:** 10.3389/fpubh.2024.1337276

**Published:** 2024-01-22

**Authors:** María Macarena Sandoval, Silvina Ruvinsky, María Carolina Palermo, Tomás Alconada, Martín Eduardo Brizuela, Eugenia Ramirez Wierzbicki, Joaquín Cantos, Ariel Bardach, Agustín Ciapponi, Paula Gagetti

**Affiliations:** ^1^Instituto de Efectividad Clínica y Sanitaria (IECS-CONICET), Buenos Aires, Argentina; ^2^Coordinación de Investigación, Hospital de Pediatría “Dr. Juan P. Garrahan”, Buenos Aires, Argentina; ^3^Unidad de Pediatría, Hospital General de Agudos Vélez Sarsfield, Buenos Aires, Argentina; ^4^Centro de Investigaciones Epidemiológicas y Salud Pública (CIESP-IECS), CONICET, Buenos Aires, Argentina; ^5^Servicio Antimicrobianos, National Reference Laboratory (NRL), Instituto Nacional de Enfermedades Infecciosas (INEI)-ANLIS “Dr. Carlos G. Malbrán”, Buenos Aires, Argentina

**Keywords:** *Streptococcus pneumoniae*, invasive pneumococcal disease, antimicrobial resistance, serotypes, Latin America

## Abstract

**Background:**

Invasive pneumococcal disease has declined since pneumococcal conjugate vaccine introduction in Latin America and the Caribbean (LAC). However, serotype distribution and antimicrobial resistance patterns have changed.

**Methods:**

We conducted a systematic review to evaluate the frequency of antimicrobial resistance of *Streptococcus pneumoniae* from invasive disease in LAC. Articles published between 1 January 2000, and 27 December 2022, with no language restriction, were searched in major databases and gray literature. Pairs of reviewers independently selected extracted data and assessed the risk of bias in the studies. The quality of antimicrobial resistance (AMR) studies was evaluated according to WHO recommendations (PROSPERO CRD42023392097).

**Results:**

From 8,600 records identified, 103 studies were included, with 49,660 positive samples of *S. pneumoniae* for AMR analysis processed. Most studies were from Brazil (29.1%) and Argentina (18.4%), were cross-sectional (57.3%), reported data on AMR from IPD cases (52.4%), and were classified as moderate risk of bias (50.5%). Resistance to penicillin was 21.7% (95%IC 18.7–25.0, I^2^: 95.9), and for ceftriaxone/cefotaxime it was 4.7% (95%IC 3.2–6.9, I^2^: 96.1). The highest resistance for both penicillin and ceftriaxone/cefotaxime was in the age group of 0 to 5 years (32.1% [95%IC 28.2–36.4, I^2^: 87.7], and 9.7% [95%IC 5.9–15.6, I^2^: 96.9] respectively). The most frequent serotypes associated with resistance were 14 for penicillin and 19A for ceftriaxone/cefotaxime.

**Conclusion:**

Approximately one-quarter of invasive pneumococcal disease isolates in Latin America and the Caribbean displayed penicillin resistance, with higher rates in young children. Ongoing surveillance is essential to monitor serotype evolution and antimicrobial resistance patterns following pneumococcal conjugate vaccine introduction.

## 1 Introduction

Invasive disease caused by *Streptococcus pneumoniae* (IPD) is one of the leading causes of morbidity and mortality in children and older adults worldwide (1). IPD included mainly meningitis, bacteremia, and bacteremic pneumonia (2).

The invasiveness and pathogenesis of *S. pneumoniae* are defined by capsular composition; currently, one hundred serotypes have been identified (3).

In Latin America and the Caribbean, it is estimated that every year, IPD is responsible for up to 28,000 deaths, 182,000 hospitalizations, and 1.4 million outpatient consults (4).

To prevent IPD, different vaccines were developed, 23-valent pneumococcal polysaccharide vaccine (PPV23) and pneumococcal conjugate vaccines (PCVs) (5). In 2009 PCVs were introduced in Latin American countries, and since May 2016, 29 countries have incorporated PCV-10 or PCV-13 s in their national immunization programs (6).

Serotype distribution in IPD changes over time by age group, clinical manifestation, and regional location (7). SIREVA is an official regional laboratory surveillance program (SIREVA) that reports information about serotype distribution and antimicrobial resistance (AMR) in IPD in the pediatric and adult populations.

*S. pneumoniae* infections are frequently associated with inappropriate antimicrobial prescriptions both in the community and in the hospital. IPD rates have decreased since the implementation of national immunization programs. However, serotype distribution and resistance patterns have been modified (8).

Antimicrobial resistance has emerged in *S. pneumoniae* during the last years with a high impact on global health. Worldwide the highest rates of resistance to penicillin and erythromycin were found in serotypes 6B, 6A, 9 V, 14, 15A, 19F, 19A, and 23F (9). However, information about serotype distribution and antimicrobials in IPD is scarce.

This systematic review aimed to describe reported data about antimicrobial resistance and associated serotypes in IPD from Latin American and Caribbean countries.

## 2 Methods

The analysis presented here was part of a broader systematic review that included epidemiological data on the burden of pneumococcal disease in LAC. The findings on AMR are presented in this article. We conducted a systematic literature review and meta-analysis of AMR in IPD in LAC during the last 20 years following Cochrane methods (10), the MOOSE guidelines for observational studies (11), and the PRISMA statement for reporting systematic reviews and meta-analyses (12). The protocol is registered in PROSPERO CRD UK (registration number: CRD42023392097).

### 2.1 Inclusion criteria

Studies from any LAC countries, regardless of age or sex, risk groups, with at least 20 culture-confirmed cases from sterile sites (e.g., blood, cerebrospinal fluid, pleural fluid) were eligible for inclusion. IPD clinical presentations included sepsis/bacteremia, meningitis, bacteremic pneumonia, empyema, peritonitis, osteoarticular infection/septic arthritis, and endocarditis.

Cohort studies, case–control studies, cross-sectional studies, case series, epidemiological surveillance reports, hospital-based surveillance studies, interrupted time series (ITS), and controlled ITS (CITS) studies were included. Systematic reviews and meta-analyses were only considered as sources for primary studies. When data or data subsets reported in more than one publication were found, the one with the larger sample size or the most recent were selected.

### 2.2 Search strategy for identification of studies and data sources

Records published between 1 January 2000, and 27 December 2022, in the following databases: PubMed, Embase, CINAHL (Cumulative Index of Nursing and Allied Health Literature), LILACS (Latin American and Caribbean Health Science Literature)/ScieLO, EconLIT, Global Health, and Web of Science, with no language restriction, were searched. Strategies search, and terms for each database are presented in Supplementary Appendix A. The reference lists of the articles were hand-searched for additional information. We contacted the original authors to obtain any missing information or clarification, but for this analysis it was not necessary.

SIREVA, other regional or national surveillance databases, including antimicrobial resistance databases, and relevant sources like regional MoH, PAHO, and reports from referral hospitals were searched. Databases containing regional proceedings, congresses’ annals, doctoral theses, websites from regional scientific meetings, experts, and related associations were also conducted.

### 2.3 Outcomes of interest

We explored outcomes by type of IPD, serotype distribution, and antimicrobial resistance reported during the same period.

### 2.4 Selection of articles and data extraction

Publications were screened by two reviewers using title and abstract according to the eligibility criteria. Discrepancies were solved by consensus of the entire work team. Potentially eligible articles were retrieved in full text for further analysis. All screening phases of the study used COVIDENCE^®^ software (13), a web-based platform designed to process systematic reviews.

One reviewer performed data extraction and verified by a second one using a pre-specified extraction online form previously piloted in 10 studies. From eligible articles, the research team extracted the following study information: publication and study characteristics (type of publication, year published, authors, geographic location, study design including domains for risk of bias assessment), study population characteristics (age, sex, sample size, population risk, inclusion and exclusion criteria), and outcomes (frequency of AMR and frequency of serotypes associated with AMR).

### 2.5 Risk of bias assessment

Included studies were assessed for risk of bias by two independent reviewers, with discrepancies resolved by consensus with the whole team. The risk of bias in observational studies and the control arm of trials was assessed using the checklists developed by the U. S. National Heart, Lung, and Blood Institute, which classify studies as high risk of bias (POOR), moderate risk of bias (FAIR), and low risk of bias (GOOD). For the assessment of cohort studies and cross-sectional studies, the tool comprises 14 items, while nine items apply to the case series studies (14).

### 2.6 Quality assessment of AMR studies

The quality of AMR studies was evaluated according to WHO recommendations (15).

### 2.7 Statistical analysis

#### 2.7.1 Primary analysis

To analyze the data, descriptive statistics and performed a proportion meta-analysis, using metaprop {meta} package with R software version 4.2.2. were used (16, 17). We applied an arcsine transformation to stabilize the variance of proportions (Freeman-Tukey variant of the arcsine square root of transformed proportions method) (18). We applied DerSimonian-Laird weights for the random effects model where heterogeneity between studies was found. We calculated the I^2^ statistics as a measure of the proportion of the overall variation in the proportion that was attributable to between-study heterogeneity. An I^2^ > 60–70% was considered as substantial heterogeneity, and below 30% as low level of heterogeneity (19). Selective reporting within studies was assessed by comparing available protocols with the reports.

#### 2.7.2 Subgroup analysis, sensitivity analysis, and investigation of heterogeneity

We conducted subgroup analyses classifying the studies by five-year calendar period, country, age group (0–5 years, 6–64 years, 65 or more years old), and by antibiotic resistance. Both types of analyses could contribute to the investigation of heterogeneity causes.

## 3 Results

### 3.1 Literature search and study selection

**Figure 1:**
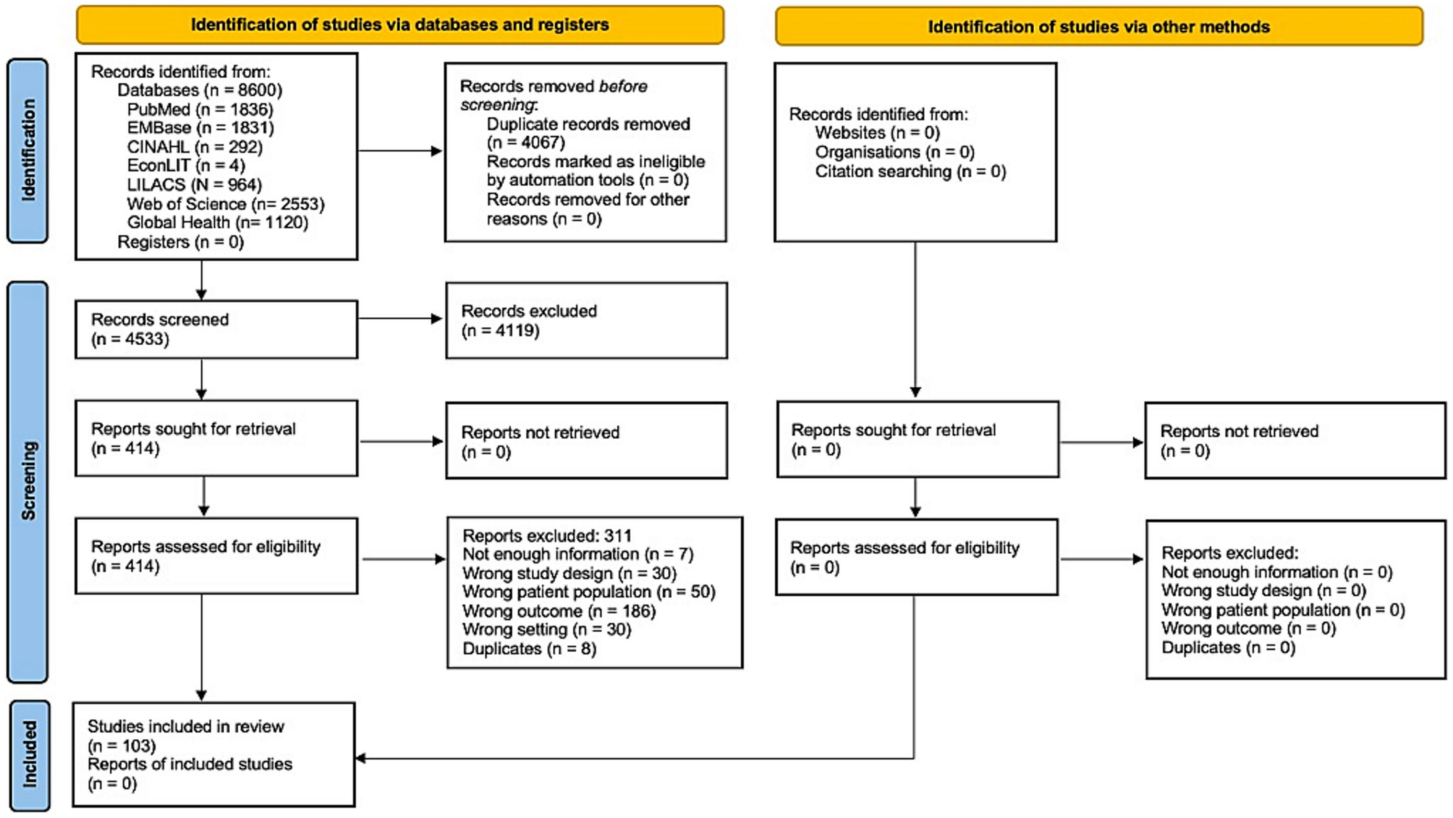
Study flowchart.

We identified 8,600 records in seven different databases. After eliminating duplicates, we screened the remaining 4,533 by title and abstract. We made a full-text assessment of 414 considered relevant to determine eligibility. Finally, 103 studies met the inclusion criteria (Figure 1).

### 3.2 Characteristics of included studies

The characteristics of the included studies are summarized in Supplementary Table 3 in Appendix A. There were 98 full texts, four abstracts, and one thesis; 59 (57.3%) were cross-sectional studies, 42 (40.9%) case series, one (0.9%) prospective cohort, and one (0.9%) non-comparative cohort.

The studies provided data on AMR in the following countries: Brazil (*n* = 30, 29.1%) between 1990 to 2019, Argentina (*n* = 19, 18.4%) between 1993 to 2019, Uruguay (*n* = 12, 11.8%) between 1987 to 2018, Colombia (*n* = 8, 7.9%) between 1994 to 2019, Chile (*n* = 7, 6.9%) between 1994 to 2014, Peru (*n* = 5, 4.9%) between 2000 to 2018, Mexico (*n* = 4, 3.9%) between 1994 to 2015, Paraguay (*n* = 4, 3.9%) between 1993 to 2018, Costa Rica (*n* = 3, 2.9%) between 1995 to 2015, Cuba (*n* = 2, 1.9%) between 2007 to 2016, French Guiana (*n* = 1, 0.9%) between 2000 to 2010, Jamaica (*n* = 1, 0.9%) between 1995 to 1999, Panama (*n* = 1, 0.9%) from 2010 and 2011, Puerto Rico (*n* = 1, 0.9%) during 2001 only, and Trinidad and Tobago (*n* = 1, 0.9%) between 1997 to 2013. There were also four studies reporting on AMR in various LAC countries (including Argentina, Bolivia, Brazil, Chile, Colombia, Costa Rica, Dominican Republic, Ecuador, El Salvador, Guatemala, Mexico, Nicaragua, Panama, Paraguay, Peru and Uruguay) between 1993 to 2015.

These studies were published between 2001 and 2022, with 38.8% (*n* = 40) published between 2000 and 2009 and 50.5% (*n* = 52) between 2010 and 2019. Only 10.7% (*n* = 11) were published in the last 3 years (2020 to 2022). The inclusion period of participants was from 1987 to 2019, with 41.7% (*n* = 43) of the studies that included participants before 2000, 47.6% (*n* = 49), which included participants between 2000 and 2009, and 10.5% (*n* = 11) included participants from 2010 onwards. Only two (1%) studies did not report the inclusion period. The reported duration of the studies ranged from 12 to 299 months.

Of the 103 studies, 54 (52.4%) reported data on AMR from IPD, 23 (22.3%) from meningitis (including two studies from IPD that only reported AMR from meningitis cases), 22 (21.4%) from pneumonia, and four (3.9%) from bacteremia. A total of 49,660 positive samples of *S. pneumoniae* for AMR analysis were processed, with a range of samples evaluated between 6 to 11,377, including 40,889 samples from IPD (range 17–11,377), 4,743 samples from pneumonia (range 11–2,629), 3,633 samples from meningitis (range 6–854), and 395 samples from bacteremia (range 56–167). In one study, the total number of samples processed was not reported.

AMR was tested for three or more antibiotics in 54.4% (*n* = 56) of the studies, two antibiotics in 23 studies (22.3%), and in the remaining 24 studies (23.3%) only for penicillin. Studies evaluating several antimicrobial resistance in IPD included principally: penicillin (*n* = 53), ceftriaxone/cefotaxime (*n* = 44), trimethoprim-sulfamethoxazole (*n* = 44) and erythromycin (*n* = 44). The methods reported in studies analyzed were epsilometric (E-test) in 26.2% (*n* = 27), broth dilution in 19.4% (n = 20), disk diffusion in 5.8% (*n* = 6), agar dilution in 3.9% (*n* = 4), automatized systems in 2.9% (*n* = 3). Only in a single study (0.9%) molecular techniques (WGS-based assessment) were used to predict antibiotic resistance from genomic data by detecting resistance genes. Also, in 25.3% (*n* = 26), combined methods were used. The method used was not reported in 15.6% (*n* = 16) of the studies.

The references of the included studies are in Supplementary Appendix A.

### 3.3 Risk of bias assessment

For cross-sectional and cohort studies, 40 (65.6%) were assessed as being at moderate (fair) risk, 15 (24.6%) were assessed as low (good) risk, and only six (9.8%) as high (poor) risk. The most frequent domains that did not meet the evaluation objectives were related to sample size justification, the evaluation of exposures more than once or different levels of exposure, and the blinding of the evaluators. For case series studies, 29 (69%) were rated as low risk, 12 (28.6%) as moderate risk, and only one (2.4%) as high risk. The domains that did not meet the objectives more frequently were whether the cases were consecutive and the description of the statistical methods. The complete evaluation of risk bias assessment by study design is in Supplementary Tables 4, 5 in Appendix A.

### 3.4 Quality assessment of AMR studies

The results of the quality assessment of AMR studies are summarized in Supplementary Table 6 in Appendix A. The quality was scored as high in 87.4% (*n* = 90) of the studies and moderate in the remaining 12.6% (*n* = 13). The most frequently missed items were not specifying whether a reference/control strain was included when assessing antimicrobial susceptibility (64.7%, *n* = 66), including less than 100 isolates, or not reporting the number of isolates evaluated (43.7%, *n* = 45).

### 3.5 Results of AMR of *Streptococcus pneumoniae* of included studies

Antimicrobial susceptibility results were interpreted according to the Clinical and Laboratory Standards Institutes (CLSI), meningitis breakpoints were used for penicillin and ceftriaxone/cefotaxime (Supplementary Table 7 in Appendix A). The percentage of resistance to penicillin from IPD cases ranged from 0% (Panama; s80) to 51.7% (Mexico; s74). In 29/50 (58%) studies, penicillin resistance was less than 25%, in 16/50 (32%) it was from 25 to 50%; and in 5/50 (10%) was more than 50%. The percentage of resistance to ceftriaxone/cefotaxime from IPD cases ranged between 0% (seven studies reported 0% of resistance; s4, s41, s50, s63, s85, s89, s92) to 26.1% (Uruguay; s74). In 36 (95%) studies, out of 38 studies analyzed, the percentage of resistance was less than 25%; in 30 (79%) the resistance was less than 10%. Only two (5%) studies reported a percentage of resistance of more than 25% (s74, s78). For trimethoprim-sulfamethoxazole, the percentage of resistance ranged from 6.1% (Brazil; s32) to 69% (Peru; s85). In eight (25%) studies, out of 32, the percentage of resistance was less than 25%, in 13 (40.6%) the resistance ranged between 25 and 50%, and in 11 (34.4%) it was more than 50%. For erythromycin, the percentage of resistance ranged from 0% (Brazil and Colombia; s35, s63) to 50% (Cuba; s68). In 23 (79.3%) studies, out of 29, the resistance was less than 25%, and in six (20.7%) it was more than 25% (Supplementary Table 8 in Appendix A).

In 23 studies from meningitis cases, 22 reported resistance to penicillin, 16 to ceftriaxone/cefotaxime, eight to trimethoprim-sulfamethoxazole, and seven to erythromycin. The percentage of resistance to penicillin ranged from 0% (Costa Rica; s65) to 64.5% (Mexico; s79). In 14/22 studies (63.6%), the resistance reported was less than 25%; in seven (31.8%), between 25 and 50%, and in one study (4.6%) more than 50%. For ceftriaxone/cefotaxime, the resistance ranged from 0% (s21, s36, s43, s48, s71, s77, s79) to 16.7% (Argentina; s13). The percentage of resistance to trimethoprim-sulfamethoxazole ranged from 9.9% (s43) to 75% (both in Brazil; s33). In three (37.5%), the resistance reported was less than 25%; in two (25%), it was from 25 to 50%, and in the remaining three (37.5%) it was more than 50%. For erythromycin, the resistance ranged from 0% (Brazil; s43) to 41.9% (Cuba; s69). In six (85.7%) studies, the resistance reported was less than 25% (Supplementary Table 9 in Appendix A).

From pneumonia cases, 24 studies were analyzed; all of them reported resistance to penicillin, 14 to ceftriaxone/cefotaxime, five to trimethoprim-sulfamethoxazole, and seven to erythromycin. Penicillin resistance ranged from 0% (s11, s55, s56, s98, s99) to 62.7% (Uruguay; s97). In 14 (58.3%) studies, the resistance was less than 25%, in nine (37.5%) it was from 25 to 50%, and only one study reported a resistance of more than 50% (s97). Resistance to ceftriaxone/cefotaxime ranged from 0% (s9, s53, s54, s55, s73, s99) to 20.3% (Mexico; s73). Trimethoprim-sulfamethoxazole resistance ranged from 33.9% (Colombia; s73) to 67.3% (Argentina; s9). In 80% of the studies, resistance was more than 50%. Erythromycin resistance was less than 25%, ranging between 2.6% (Argentina; s73) to 24.5% (Mexico; s73; Supplementary Table 10 in Appendix A).

Only four studies reported antimicrobial susceptibility from bacteremia cases, with 395 isolates evaluated for penicillin, 163 for ceftriaxone/cefotaxime, and 56 for both trimethoprim-sulfamethoxazole and erythromycin. The percentage of resistance to penicillin ranged from 0% (Argentina; s17) to 20.6% (Argentina; s2). Two studies reported the resistance to ceftriaxone/cefotaxime, it was 0% in both (Argentina and Chile; s2, s51). One study reported the percentage of resistance to trimethoprim-sulfamethoxazole and erythromycin, with 17.9% of resistance each (s51; Supplementary Table 11 in Appendix A).

The full assessment of susceptibility of *S. pneumoniae* is in the Supplementary Tables 12–15 in Appendix B.

### 3.6 Serotypes associated with antimicrobial resistance

**Figure 2:**
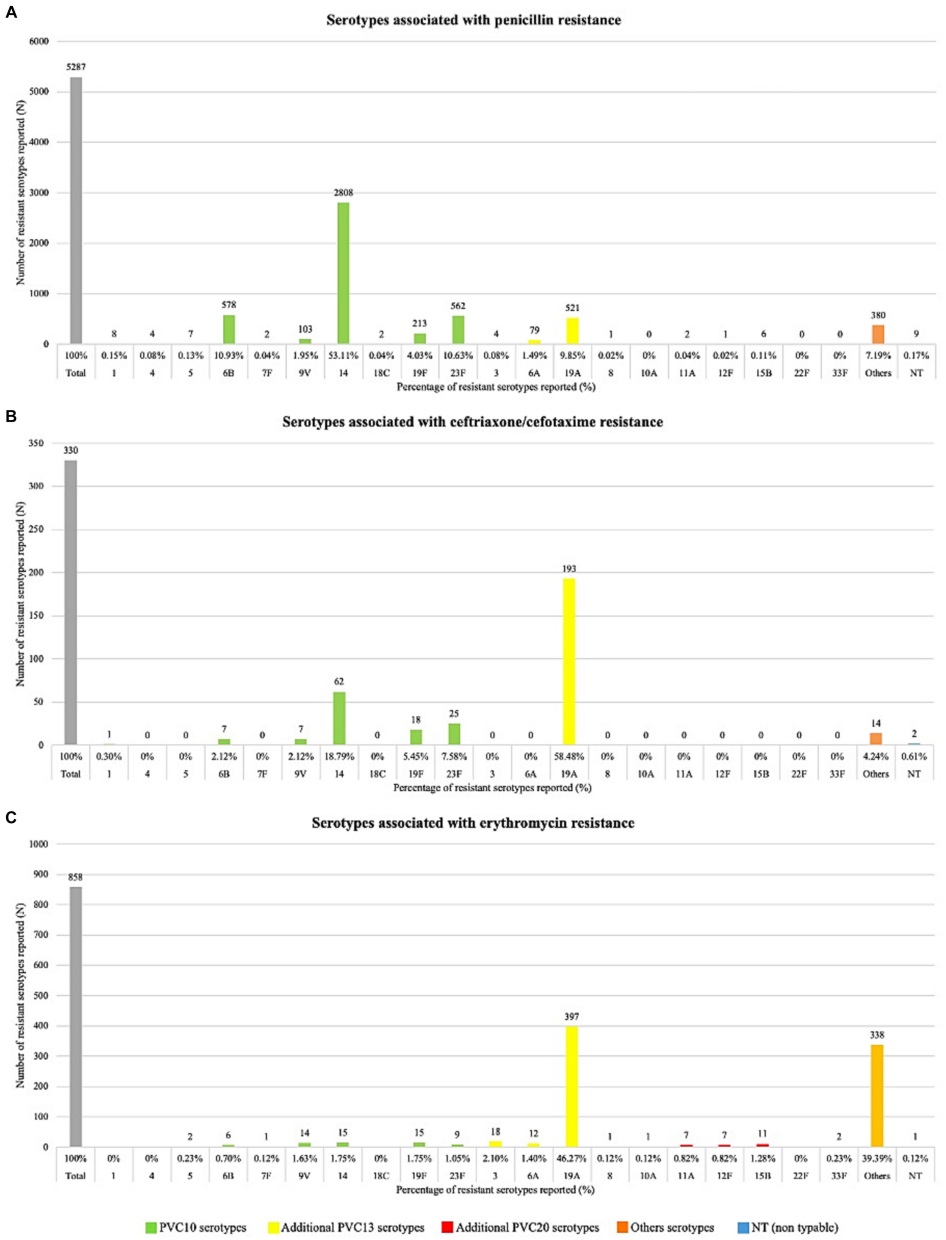
Serotypes associated with resistance. Serotypes associated with resistance to: (panel **A**) penicillin, (panel **B**) ceftriaxone/cefotaxime, (panel **C**) erythromycin.

Forty-two studies (40.7%) reported the serotypes associated with antimicrobial resistance. All studies analyzed penicillin resistance, in 11 ceftriaxone/cefotaxime and the remaining seven erythromycin resistance. The most frequent serotype associated with penicillin resistance was serotype 14 (53.11%, *n* = 2,808), followed by serotypes 6B (10.93%, *n* = 578), 23F (10.63%, *n* = 562), and 19A (9.85%, *n* = 521). 5/42 (12%) studies only include serotype 19A isolates for analysis. The majority of serotypes related to AMR found are included in 518 the PVC13 vaccine. Additional serotypes included in the PVC20 519 vaccine were found much less frequently, with only 10 resistant 520 isolates serotyped as 15B (0.11%), 11A (0.04%), 12F and 8 (0.02% 521 each), resistant serotypes 10A, 22F and 33F were not reported 522 (Figure 2A).

For ceftriaxone/cefotaxime and erythromycin, the most frequent serotypes associated with resistance was 19A (58.48%, *n* = 193 and 46.27%, *n* = 397 respectively). In the case of ceftriaxone/cefotaxime, another five serotypes were reported associated with resistance but with a lower frequency, all included in the PVC10 vaccine (Figure 2B). Regarding erythromycin, except for serotypes 1, 4, 18C, and 22F, all the other serotypes included in the PVC10, PVC13, 529 and PVC20 vaccines were associated with resistance, with a frequency less than 5% (Figure 2C).

The complete assessment of serotypes associated with resistance is in Supplementary Tables 16–18 are in Appendix B.

### 3.7 Proportion meta-analysis for resistance to penicillin and ceftriaxone/cefotaxime

A proportion meta-analysis for resistance to penicillin and ceftriaxone/cefotaxime was performed. Subgroup analyses were conducted by five-year calendar period, country, and age range (0–5 years, 6–64 years, and 65 or more years; Table 1).

**Table 1:** Summary of proportion meta-analyses results for resistance to penicillin and ceftriaxone/cefotaxime, by five-year calendar period, age categories and country.

	Resistance to penicillin			Resistance to ceftriaxone/cefotaxime		
	Studies (N)	Proportion (95%IC)	I^2^	Studies (N)	Proportion (95%IC)	I^2^
Overall	92	21.71% (18.70–25.07)	95.9%	47	4.74% (3.21–6.94)	96.1%
By 5-years period						
1990–1994	2	24.99% (16.55–35.89)	27.5%	–	–	–
1995–1999	22	28.44% (23.69–33.71)	93.4%	8	11.28% (6.29–19.39)	79.9%
2000–2004	14	35.82% (27.14–45.55)	93.4%	12	7.03% (2.16–20.53)	93.5%
2005–2009	6	8.09% (3.66–16.93)	70.2%	9	8.03% (2.85–20.60)	92.9%
2010–2014	4	5.96% (1.22–24.53)	94.8%	10	5.34% (3.01–9.29)	59.1%
2015–2019	–	–	–	4	6.52% (3.58–11.58)	76.0%
By age						
0–5 years	35	32.17% (28.23–36.40)	87.7%	20	9.78% (5.98–15.61)	96.9%
6–64 years	2	15.66% (12.91–18.87)	80.3%	1	6.20% (5.55–6.93)	NA
≥65 years	3	18.25% (9.75–31.56)	92.9%	2	5.13% (4.47–5.87)	0%
By country						
Argentina	18	19.54% (13.00–28.31)	94.0%	9	6.74% (3.30–13.29)	97.2%
Brazil	31	20.09% (16.30–24.50)	94.0%	12	2.48% (0.97–6.18)	95.8%
Chile	6	11.69% (4.06–29.27)	83.5%	2	5.76% (1.74–17.41)	68.7%
Colombia	9	19.72% (15.34–24.97)	94.7%	8	4.14% (1.75–9.49)	96.3%
Costa Rica	2	27.77% (6.90–66.62)	92.6%	1	3.03% (0.76–11.32)	NA
Cuba	1	44.76% (35.55–54.35)	NA	1	7.62% (3.86–14.50)	NA
French Guiana	1	35.71% (20.41–54.62)	NA	–	–	–
Mexico	6	45.56% (33.31–58.37)	88.1%	4	17.30% (9.50–29.42)	84.6%
Paraguay	4	16.34% (8.66–28.68)	89.3%	4	1.64% (0.41–6.39)	72.7%
Peru	3	18.17% (13.51–24.00)	0%	2	3.10% (1.56–6.08)	0%
Puerto Rico	1	49.72% (42.41–57.04)	NA	1	3.39% (1.53–7.34)	NA
Uruguay	10	22.75% (9.13–46.30)	92.8%	4	6.69% (1.92–20.79)	96.3%

NA, Not Applicable.

**Figure 3:**
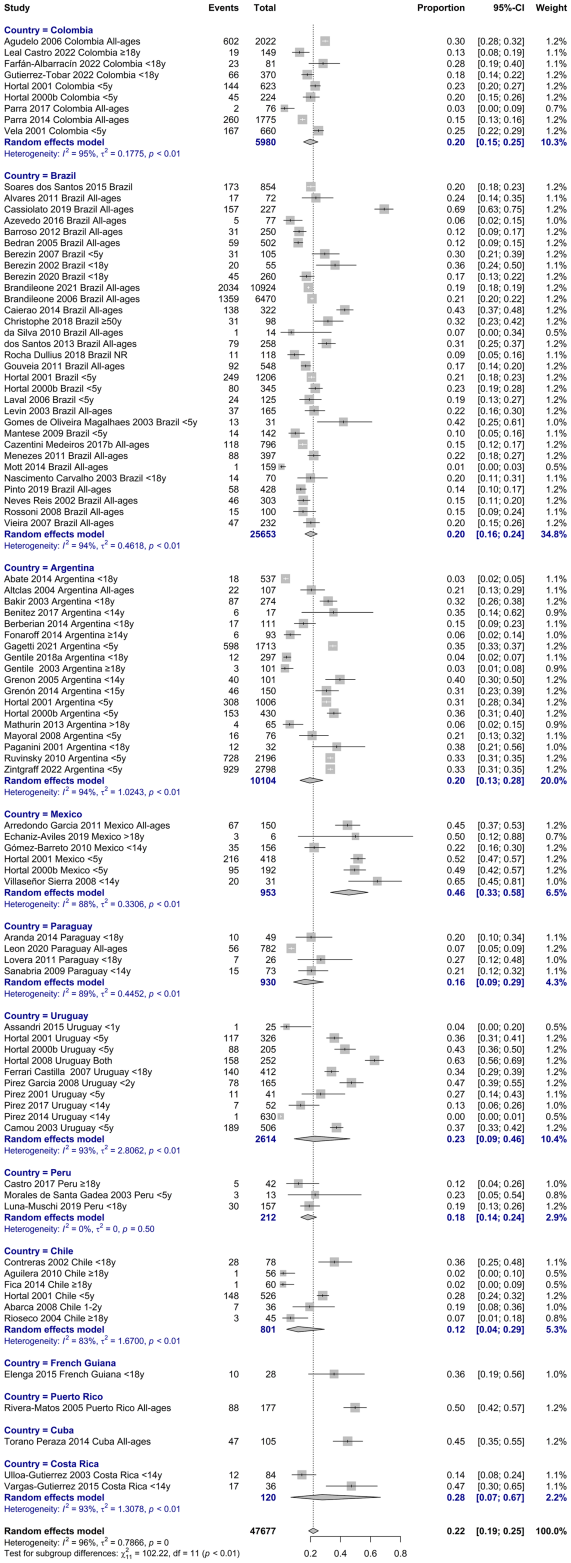
Proportion meta-analysis of resistance to penicillin by country.

The resistance to penicillin was 21.7% (95%IC 18.7–25.0, I^2^: 95.9; Supplementary Figure 1 in Appendix A). The highest resistance was observed between 2000 and 2004 with 35.8% (95%IC 27.1–45.5, I^2^: 93.4), and the lowest between 2010 and 2014 with 5.9% (95%IC 1.2–24.5, I^2^: 94.8; Supplementary Figure 2 in Appendix A). Regarding age, the highest resistance was in the age group of 0 to 5 years with 32.1% (95%IC 28.2–36.4, I^2^: 87.7; Supplementary Figure 3 in Appendix A). When we analyzed by country we observed the highest resistance in Puerto Rico: 49.7% (95%IC 42.4–57.0, I^2^: NA), Mexico: 45.5% (95%IC 33.3–58.3, I^2^: 88.1), and Cuba: 44.7% (95%IC 35.5–54.3, I^2^: NA), while in Chile the lowest resistance was observed with 11.6% (95%IC 4.0–29.2, I^2^: 83.5; Figure 3).

**Figure 4:**
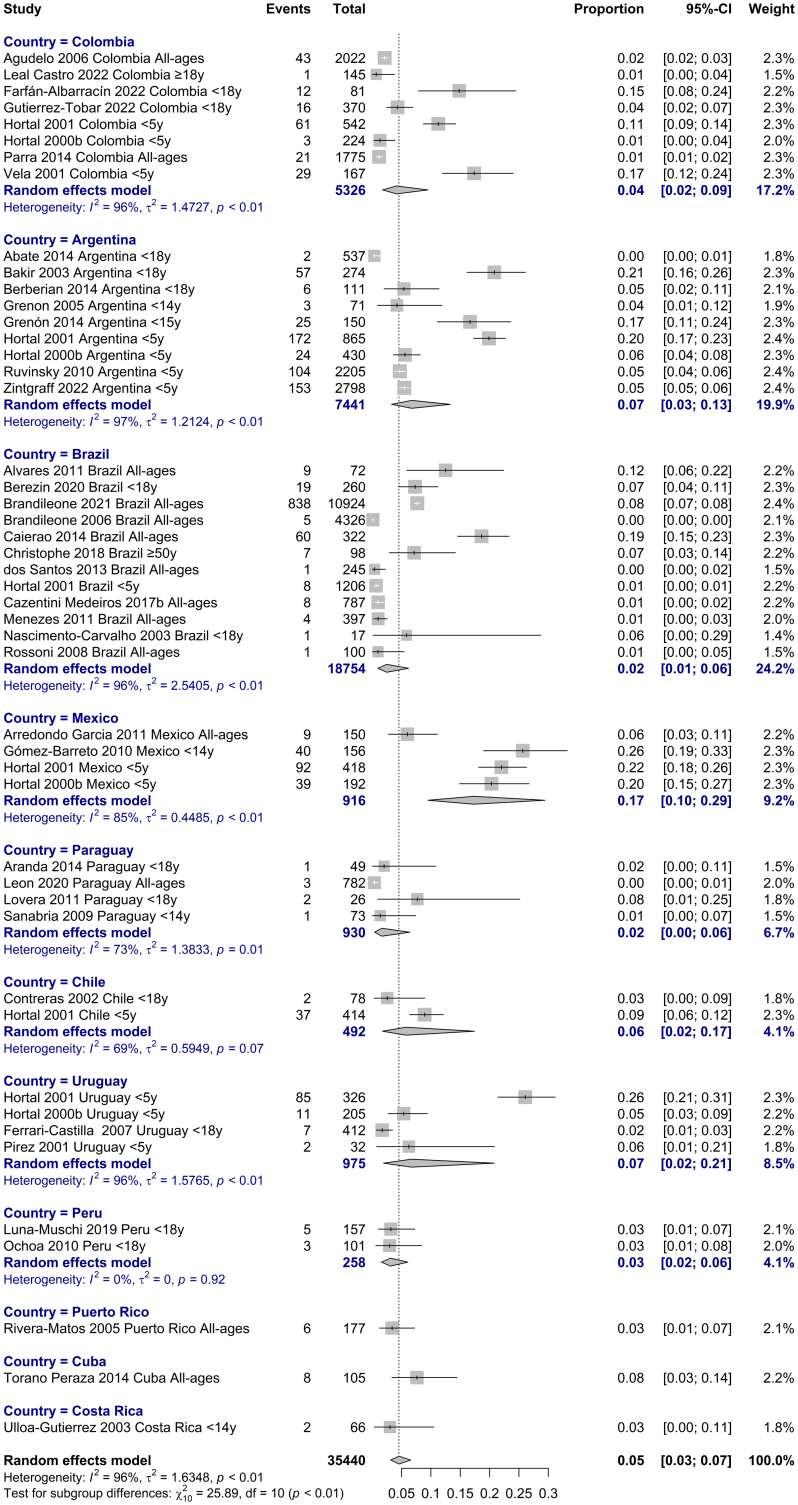
Proportion meta-analysis of resistance to ceftriaxone/cefotaxime by country.

The resistance to ceftriaxone/cefotaxime was 4.7% (95%IC 3.2–6.9, I^2^: 96.1; Supplementary Figure 4 in Appendix A). Between 1995 and 1999 we observed the highest resistance with 11.2% (95%IC 6.2–19.3, I^2^: 79.9; Supplementary Figure 5 in Appendix A). As with penicillin, the highest resistance was in the age group of 0 to 5 years with 9.7% (95%IC 5.9–15.6, I^2^: 96.9; Supplementary Figure 6 in Appendix A). Analyzed by country the resistance in all was less than 10%, except in Mexico where we observed the highest with 17.3% of resistance (95%IC 9.5–29.4, I^2^: 84.6; Figure 4).

### 3.8 Data analysis from regional laboratory surveillance database reports (SIREVA)

**Figure 5:**
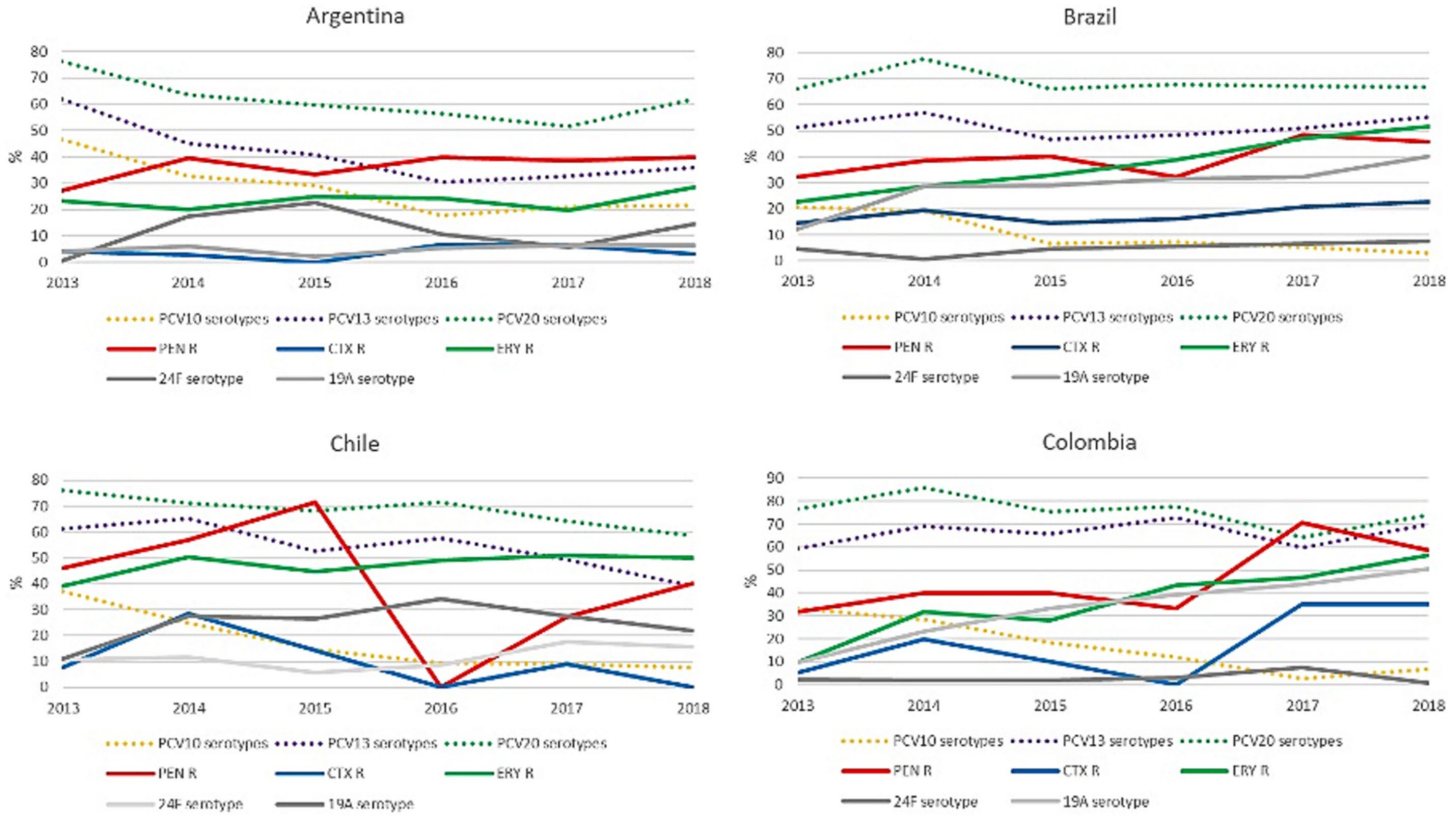
Variation over time of resistance to penicillin, ceftriaxone/cefotaxime and erythromycin, and the percentage of serotypes included in the different PCVs. Figure 4 shows the percentage of resistance to penicillin, ceftriaxone/cefotaxime and erythromycin, and the percentage of serotype 19A and 24F isolates between 2013 and 2018, as well as the percentage of serotypes included in PCV10, PCV13 and PCV20 (dotted lines). PEN: penicillin; CTX: ceftriaxone/cefotaxime; ERY: erythromycin; R: resistance. PCV10 was implemented in Brazil in March 2010 and in Chile in January 2011. PCV13 was implemented in Argentina in January 2012. In Colombia PCV13 was implemented in July 2011 and replaced by PCV10 in January 2012.

Among the countries of the region of the Americas most represented in SIREVA reports were Argentina, Brazil, Chile, and Colombia, which between 2013 and 2018 reported more than 600 results from children under 5 years old. Before 2013, non-PCV13 serotypes were not listed. Resistance to penicillin and cefotaxime did not present significant differences between 2013 and 2018 in any of the countries analyzed. Regarding erythromycin resistance, in Argentina, a significant increase (*p* < 0.05) was observed between 2013 and 2018. Analyzing the main serotypes related to antimicrobial resistance, when comparing 2013 with 2018, a significant increase in 19A was observed in Chile, as well as 24A in Argentina and Chile (Figure 5).

## 4 Discussion

This study analyzed mainly antimicrobial resistance and associated serotypes in IPD previous and post-introduction of PCVs in pediatric, adult, and mixed populations in healthcare facilities from 15 countries across the LAC region.

Antimicrobial resistance in *S. pneumoniae* changes over time depending on PCV implementation, serotype distribution, antimicrobial consumption, and other factors showed differences between countries (7, 20).

Since the introduction of PCVs in national immunization programs in LAC, a decrease in IPD and changes in serotype distribution and antimicrobial resistance patterns have been observed. We focus on antibiotics useful to treat pneumococcal diseases in children and adults (21, 22). In our study, we observed a global penicillin antimicrobial resistance rate was less than 50% in IPD in the LAC region; similar data was reported in other regions (23).

Over the years, penicillin resistance increased until it reached its highest level in 2000–2004, followed by a decline that, despite some fluctuations, coincided with the introduction of PCVs in the different countries of the region between 2008 and 2015 (6, 24). The highest rates of resistance were observed in children under 5 years, followed by adults older than 65. Resistance rates between 40 and 50% were reported in the majority before the introduction of PCVs in these countries. In countries from the region, penicillin rates higher than 50% were reported mainly in studies that include only 19A isolates. Despite the introduction of PCVs, serotype 19A remains among the most frequently associated with antimicrobial resistance and multidrug resistance (25). In the pre-PCV period, the studies that reported resistance to penicillin found it mainly associated with serotype 14.

Analysis of serotypes associated with penicillin resistance revealed a prevalence of serotypes 14, 23F, 6B, and other PCV10 serotypes in all studies conducted before the introduction of PCVs, and was consistent with the global pattern and previous reports from LAC countries (26).

In a study conducted in Brazil, the emergence of non-PCV10 serotypes 19A and 6A penicillin-resistant isolates was observed in the post-PCV period (20). As a consequence the study describes an increase of antimicrobial non-susceptibility in a long-term post-PCV10 introduction.

A similar scenario was observed for ceftriaxone/cefotaxime-resistant strains, which were associated mainly with serotypes 14, 23F, and 19F in pre-vaccination studies and associated with serotype 19A and other new serotypes in post-vaccination studies.

Interestingly, outcomes from our study showed an increase in erythromycin resistance during the study period mainly related to serotype 19A and other non-vaccine serotypes. No changes in penicillin resistance, increased resistance to erythromycin, tetracycline, and multidrug resistance in the post-PCVs period were observed. In line with our results, the proportion of pneumococci showing resistance to first-line antimicrobials has decreased after vaccination. However, higher rates of resistance to other antimicrobials, mainly macrolides, have been observed in several countries, despite overall reductions in IPD attributable to vaccines, frequently associated with non-vaccine serotypes (27–29). Serotype 24F appears as an emergent serotype related to multidrug resistance and is characterized by its high invasiveness and probably influenced by antibiotic consumption (30).

One of the primary limitations of the study was the risk of bias of the included studies, which was mainly due to low sample sizes, selection bias, and information bias in the outcome measurement. Although, the risk assessment for cross-sectional and cohort studies showed that the majority were classified as moderate risk, and case series studies were mostly rated as low risk. Most of the studies included were case series and cross-sectional, which did not provide the best disease estimations. We try to exclude, as far as possible, those articles whose data seemed to be published in more than one article, but it is possible that some AMR data found have been reported by several studies. In Latin American and Caribbean countries (excluding pneumococcal meningitis), it is not mandatory to report to laboratory-based systems like SIREVA, and passive surveillance may not accurately reflect the prevalence of diseases.

According to this study, the treatment of choice for pneumonia remains penicillin or ampicillin and cefotaxime or ceftriaxone for meningitis and bacteremia.

The study provides data on AMR and associated serotypes throughout the LAC counties during pre- and post-vaccine periods, including the late post-vaccination period, which is very important to assess the changes produced by incorporating PCVs in the region. Our results highlight continuous surveillance’s importance in assessing the dynamic of serotype distribution and antimicrobial resistance in pediatric and adult IPD from LAC.

## 5 Conclusion

The introduction of PCVs in LAC countries has led to changes in pneumococcal serotype distribution and antimicrobial resistance patterns in IPD. There was an overall decline in antibiotic resistance, particularly to penicillin, after PCV implementation. However, concerning trends of increased erythromycin resistance and the emergence of non-vaccine serotypes associated with antibiotic resistance highlight the need for ongoing surveillance. Continuous monitoring of serotype evolution and antimicrobial resistance is essential to evaluate PCV impact and guide treatment recommendations for pneumococcal disease in Latin America and the Caribbean.

## Data availability statement

The original contributions presented in the study are included in the article/Supplementary material, further inquiries can be directed to the corresponding author.

## Author contributions

MS: Data curation, Investigation, Writing – original draft, Writing – review & editing. SR: Conceptualization, Formal analysis, Investigation, Methodology, Resources, Supervision, Writing – original draft, Writing – review & editing. MP: Data curation, Investigation, Writing – original draft, Writing – review & editing. TA: Data curation, Investigation, Writing – original draft, Writing – review & editing. MB: Data curation, Investigation, Writing – original draft, Writing – review & editing. EW: Investigation, Writing – review & editing. JC: Formal analysis, Investigation, Writing – review & editing. AB: Conceptualization, Funding acquisition, Investigation, Methodology, Project administration, Resources, Supervision, Writing – original draft, Writing – review & editing. AC: Conceptualization, Formal analysis, Funding acquisition, Investigation, Methodology, Project administration, Resources, Supervision, Writing – original draft, Writing – review & editing. PG: Formal analysis, Investigation, Methodology, Supervision, Writing – original draft, Writing – review & editing.
