# Supplementary material for: Antimicrobial resistance of Streptococcus pneumoniae from invasive pneumococcal diseases in Latin American countries: a systematic review and meta-analysis
Source: Front Public Health. 2024 Jan 22;12:1337276. doi: 10.3389/fpubh.2024.1337276 (PMC10839967; doi:10.3389/fpubh.2024.1337276)
Supplement: Supplementary file 1 [file Data_Sheet_1.docx]

***Index Supplementary Material Appendix A***

# Supplementary Table 1. PRISMA checklist

| **Section and Topic** | **Item #** | **Checklist item** | **Location where item is reported** |
| --- | --- | --- | --- |
| **TITLE** | | |  |
| Title | 1 | Identify the report as a systematic review. | Title |
| **ABSTRACT** | | |  |
| Abstract | 2 | See the PRISMA 2020 for Abstracts checklist. | Abstract |
| **INTRODUCTION** | | |  |
| Rationale | 3 | Describe the rationale for the review in the context of existing knowledge. | Section 1 |
| Objectives | 4 | Provide an explicit statement of the objective(s) or question(s) the review addresses. | Section 1 |
| **METHODS** | | |  |
| Eligibility criteria | 5 | Specify the inclusion and exclusion criteria for the review and how studies were grouped for the syntheses. | Section 2.1 |
| Information sources | 6 | Specify all databases, registers, websites, organisations, reference lists and other sources searched or consulted to identify studies. Specify the date when each source was last searched or consulted. | Section 2.2 and Appendix A |
| Search strategy | 7 | Present the full search strategies for all databases, registers and websites, including any filters and limits used. | Appendix A |
| Selection process | 8 | Specify the methods used to decide whether a study met the inclusion criteria of the review, including how many reviewers screened each record and each report retrieved, whether they worked independently, and if applicable, details of automation tools used in the process. | Section 2.4 |
| Data collection process | 9 | Specify the methods used to collect data from reports, including how many reviewers collected data from each report, whether they worked independently, any processes for obtaining or confirming data from study investigators, and if applicable, details of automation tools used in the process. | Section 2.4 |
| Data items | 10a | List and define all outcomes for which data were sought. Specify whether all results that were compatible with each outcome domain in each study were sought (e.g. for all measures, time points, analyses), and if not, the methods used to decide which results to collect. | Section 2.3 and Section 2.4 |
|  | 10b | List and define all other variables for which data were sought (e.g. participant and intervention characteristics, funding sources). Describe any assumptions made about any missing or unclear information. | Section 2.4 |
| Study risk of bias assessment | 11 | Specify the methods used to assess risk of bias in the included studies, including details of the tool(s) used, how many reviewers assessed each study and whether they worked independently, and if applicable, details of automation tools used in the process. | Section 2.5 |
| Effect measures | 12 | Specify for each outcome the effect measure(s) (e.g. risk ratio, mean difference) used in the synthesis or presentation of results. | Section 2.4 |
| Synthesis methods | 13a | Describe the processes used to decide which studies were eligible for each synthesis (e.g. tabulating the study intervention characteristics and comparing against the planned groups for each synthesis (item #5)). | Not described |
|  | 13b | Describe any methods required to prepare the data for presentation or synthesis, such as handling of missing summary statistics, or data conversions. | Not described |
|  | 13c | Describe any methods used to tabulate or visually display results of individual studies and syntheses. | Not described |
|  | 13d | Describe any methods used to synthesize results and provide a rationale for the choice(s). If meta-analysis was performed, describe the model(s), method(s) to identify the presence and extent of statistical heterogeneity, and software package(s) used. | Section 2.7.1 |
|  | 13e | Describe any methods used to explore possible causes of heterogeneity among study results (e.g. subgroup analysis, meta-regression). | Section 2.7.2 |
|  | 13f | Describe any sensitivity analyses conducted to assess robustness of the synthesized results. | Not described |
| Reporting bias assessment | 14 | Describe any methods used to assess risk of bias due to missing results in a synthesis (arising from reporting biases). | Not described |
| Certainty assessment | 15 | Describe any methods used to assess certainty (or confidence) in the body of evidence for an outcome. | Not described |
| **RESULTS** | | |  |
| Study selection | 16a | Describe the results of the search and selection process, from the number of records identified in the search to the number of studies included in the review, ideally using a flow diagram. | Section 3.1 and Figure 1 |
|  | 16b | Cite studies that might appear to meet the inclusion criteria, but which were excluded, and explain why they were excluded. | Appendix A: Table 2 |
| Study characteristics | 17 | Cite each included study and present its characteristics. | Section 3.2 and Appendix A: Table 3 |
| Risk of bias in studies | 18 | Present assessments of risk of bias for each included study. | Section 3.3 and Appendix A: Table 4 and 5 |
| Results of individual studies | 19 | For all outcomes, present, for each study: (a) summary statistics for each group (where appropriate) and (b) an effect estimate and its precision (e.g. confidence/credible interval), ideally using structured tables or plots. | Appendix A: Table 8, 9, 10 and 11 |
| Results of syntheses | 20a | For each synthesis, briefly summarise the characteristics and risk of bias among contributing studies. | NA |
|  | 20b | Present results of all statistical syntheses conducted. If meta-analysis was done, present for each the summary estimate and its precision (e.g. confidence/credible interval) and measures of statistical heterogeneity. If comparing groups, describe the direction of the effect. | Table 1 |
|  | 20c | Present results of all investigations of possible causes of heterogeneity among study results. | Not described |
|  | 20d | Present results of all sensitivity analyses conducted to assess the robustness of the synthesized results. | Not described |
| Reporting biases | 21 | Present assessments of risk of bias due to missing results (arising from reporting biases) for each synthesis assessed. | NA |
| Certainty of evidence | 22 | Present assessments of certainty (or confidence) in the body of evidence for each outcome assessed. | Not described |
| **DISCUSSION** | | |  |
| Discussion | 23a | Provide a general interpretation of the results in the context of other evidence. | Section 4 |
|  | 23b | Discuss any limitations of the evidence included in the review. | Section 4 |
|  | 23c | Discuss any limitations of the review processes used. | Section 4 |
|  | 23d | Discuss implications of the results for practice, policy, and future research. | Section 4 |
| **OTHER INFORMATION** | | |  |
| Registration and protocol | 24a | Provide registration information for the review, including register name and registration number, or state that the review was not registered. | Section 2 |
|  | 24b | Indicate where the review protocol can be accessed, or state that a protocol was not prepared. | Section 2 |
|  | 24c | Describe and explain any amendments to information provided at registration or in the protocol. | NA |
| Support | 25 | Describe sources of financial or non-financial support for the review, and the role of the funders or sponsors in the review. | Funding |
| Competing interests | 26 | Declare any competing interests of review authors. | Declaration of competing interest |
| Availability of data, code and other materials | 27 | Report which of the following are publicly available and where they can be found: template data collection forms; data extracted from included studies; data used for all analyses; analytic code; any other materials used in the review. | Data availability |

# Search strategy

Name of the Database: Pubmed (MEDLINE)

Date of search: 27/12/2022

| Search | Query |
| --- | --- |
| #16 | #13 AND #14 Filters: from 2000/1/1 - 3000/12/12 |
| #15 | #13 AND #14 |
| #14 | (Americas[Majr] OR Latin America[Mesh] OR Latin America*[tiab] OR Latinamerica*[tiab] OR Latinoamerica*[tiab] OR Hispanoamerica*[tiab] OR Iberoamerica*[tiab] OR Ibero Americ*[tiab] OR Panamerican*[tiab] OR Central America[Mesh] OR Central America*[tiab] OR Centroamerica*[tiab] OR Mesoamerica*[tiab] OR Meso America*[tiab] OR Middle America*[tiab] OR South America[Mesh] OR South America*[tiab] OR Southamerica*[tiab] OR Sudamerica*[tiab] OR "America del Sur"[tiab] OR Caribbean Region[Mesh] OR Caribbean[tiab] OR Caribe*[tiab] OR West Indies[Mesh] OR West Indi*[tiab] OR Antill*[tiab] OR Indians, South American[Mesh] OR Indians, Central American[Mesh] OR Amerindian*[tiab] OR Indians[tiab] OR American Indian*[tiab] OR Native America*[tiab] OR Patagoni*[tiab] OR Andes[tiab] OR Andean*[tiab] OR Amazon*[tiab] OR Anguilla[ad] OR Anguill*[tiab] OR Anguilla[pl] OR "Antigua and Barbuda"[ad] OR "Antigua and Barbuda"[tiab] OR "Antigua and Barbuda"[pl] OR Argentin*[ad] OR Argentin*[tiab] OR Argentina[pl] OR Bahama*[ad] OR Baham*[tiab] OR Bahama*[pl] OR Bermud*[ad] OR Bermud*[tiab] OR Bermud*[pl] OR Bolivia*[ad] OR Bolivia*[tiab] OR Bolivia[pl] OR Brazil*[ad] OR Brasil*[ad] OR Brazil*[tiab] OR Brasil*[tiab] OR Brazil[pl] OR Cayman*[ad] OR Cayman*[tiab] OR Cayman*[pl] OR Curaçao[ad] OR Curaçao[tiab] OR Curaçao[pl] OR Colombia*[ad] OR Colombia*[tiab] OR Colombia[pl] OR Chile*[ad] OR Chile*[tiab] OR Chile[pl] OR Ecuador*[ad] OR Ecuator*[ad] OR Ecuador*[tiab] OR Ecuador[pl] OR Grenad*[ad] OR Grenad*[tiab] OR Grenad*[pl] OR Guadeloup*[ad] OR Guadeloup*[tiab] OR Guadeloup*[pl] OR Guiana*[ad] OR Guiana*[tiab] OR French Guiana[pl] OR Guyan*[ad] OR Guyan*[tiab] OR Guyana[pl] OR Paraguay*[ad] OR Paraguay*[tiab] OR Paraguay[pl] OR Peru*[ad] OR Peru*[tiab] OR Peru[pl] OR Surinam*[ad] OR Surinam*[tiab] OR Surinam*[pl] OR Uruguay*[ad] OR Uruguay*[tiab] OR Uruguay[pl] OR Venez*[ad] OR Venez*[tiab] OR Venezuela[pl] OR Belize*[ad] OR Belize*[tiab] OR Belize[pl] OR Costa Ric*[ad] OR Costarric*[ad] OR Costaric*[ad] OR Costa Ric*[tiab] OR Costarric*[tiab] OR Costaric*[tiab] OR Costa Rica[pl] OR Salvador*[ad] OR Salvador*[tiab] OR El Salvador[pl] OR Guatemal*[ad] OR Guatemal*[tiab] OR Guatemala[pl] OR Hondur*[ad] OR Hondur*[tiab] OR Honduras[pl] OR Martinique[ad] OR Martiniqu*[tiab] OR Martinique[pl] OR Nicaragu*[ad] OR Nicaragu*[tiab] OR Nicaragua[pl] OR Panam*[ad] OR Panam*[tiab] OR Panama[pl] OR Mexico[Mesh] OR Mexic*[ad] OR Mexic*[tiab] OR Mejic*[tiab] OR Mexico[pl] OR Montserrat[ad] OR Montserrat*[tiab] OR Montserrat[pl] OR Baham*[ad] OR Baham*[tiab] OR Bahamas[pl] OR Cuba*[ad] OR Cuba*[tiab] OR Cuba[pl] OR Dominic*[ad] OR Dominic*[tiab] OR Dominican Republic[pl] OR Haiti*[ad] OR Haiti*[tiab] OR Haiti[pl] OR Jamaic*[ad] OR Jamaic*[tiab] OR Jamaica[pl] OR Puerto Rico[Mesh] OR Puerto Ric*[tiab] OR Puertorric*[tiab] OR Puertoric*[tiab] OR Saint Kitts[ad] OR Saint Kitts[tiab] OR Saint Kitts[pl] OR "Trinidad and Tobago"[tiab] OR "Trinidad and Tobago"[ad] OR "Trinidad and Tobago"[pl]) |
| #13 | #1 OR #2 OR #3 OR #4 OR #5 OR #6 OR #7 OR #8 OR #9 OR #10 OR #11 OR #12 |
| #12 | PPV23[tiab] |
| #11 | PCV 15[tiab] |
| #10 | PCV 13[tiab] |
| #9 | PCV 10[tiab] |
| #8 | Pneumovax[tiab] |
| #7 | PnuImune Vaccine*[tiab] |
| #6 | Pnu-Imune Vaccine*[tiab] |
| #5 | Pneumococcal Vaccines[Mesh] |
| #4 | IPD[tiab] |
| #3 | Pneumococc*[tiab] |
| #2 | Streptococcus Pneumoniae[Mesh] |
| #1 | Pneumococcal Infections[Mesh] |

Name of Database: EMBase (OVID)

Date of search: 27/12/2022

Embase <1974 to 2022 December 23>

| # | Query |
| --- | --- |
| 1 | exp pneumococcal infection/ |
| 2 | exp Streptococcus pneumoniae/ |
| 3 | Pneumococc*.ti,ab. |
| 4 | IPD.ti,ab. |
| 5 | exp Pneumococcus vaccine/ |
| 6 | (Pnu-Imune adj3 Vaccine*).ti,ab. |
| 7 | (PnuImune adj3 Vaccine*).ti,ab. |
| 8 | Pneumovax.ti,ab. |
| 9 | PCV-10.ti,ab. |
| 10 | PCV-13.ti,ab. |
| 11 | PCV-15.ti,ab. |
| 12 | PPV23.ti,ab. |
| 13 | or/1-12 83268 |
| 14 | exp Latin America/ or exp South America/ or exp Central America/ or (Latin adj1 America*).ti,ab. or Latinamerica*.ti,ab. or Latinoamerica*.ti,ab. or Hispanoamerica.ti,ab. or Iberoamerica*.ti,ab. or (Ibero adj1 Americ*).ti,ab. or Panamerica*.ti,ab. or (South adj1 America*).ti,ab. or Southamerica*.ti,ab. or Sudamerica*.ti,ab. or (America adj1 Sur).ti,ab. or (Central adj1 America*).ti,ab. or Centroamerica*.ti,ab. or Mesoamerica*.ti,ab. or (Meso adj1 America*).ti,ab. or (Middle adj1 America*).ti,ab. or exp Caribbean Islands/ or Caribbean*.ti,ab. or Caribe*.ti,ab. or (West adj1 Indi*).ti,ab. or Antill*.ti,ab. or exp American indian/ or Amerindian*.ti,ab. or Indians.ti,ab. or (Native adj1 America*).ti,ab. or Patagoni*.ti,ab. or Andes.ti,ab. or Andean*.ti,ab. or Amazon*.ti,ab. or exp Argentina/ or Argentin*.ti,ab. or exp Bolivia/ or Bolivia*.ti,ab. or exp Brazil/ or Brazil*.ti,ab. or Brasil*.ti,ab. or exp Colombia/ or Colombia*.ti,ab. or exp Chile/ or Chile*.ti,ab. or exp Ecuador/ or Ecuador*.ti,ab. or exp French Guiana/ or Guiana*.ti,ab. or exp Guyana/ or Guyan*.ti,ab. or exp Paraguay/ or Paraguay*.ti,ab. or exp Peru/ or Peru*.ti,ab. or exp Suriname/ or Surinam*.ti,ab. or exp Uruguay/ or Uruguay*.ti,ab. or exp Venezuela/ or Venez*.ti,ab. or exp Belize/ or Beliz*.ti,ab. or exp Costa Rica/ or (Costa adj1 Rica).ti,ab. or Costarric*.ti,ab. or Costaric*.ti,ab. or exp El salvador/ or Salvador*.ti,ab. or exp Guatemala/ or Guatemal*.ti,ab. or exp Honduras/ or Hondur*.ti,ab. or exp Nicaragua/ or Nicaragu*.ti,ab. or exp Panama/ or Panam*.ti,ab. or exp Mexico/ or Mexic*.ti,ab. or exp Cuba/ or Cuba*.ti,ab. or exp Dominican Republic/ or Dominica*.ti,ab. or exp Haiti/ or Haiti*.ti,ab. or exp Jamaica/ or Jamaic*.ti,ab. or exp Puerto Rico/ or (Puerto adj1 Ric*).ti,ab. or Puertoric*.ti,ab. or Puertorric*.ti,ab. |
| 15 | 13 and 14 |
| 16 | limit 15 to yr="2000 -Current" |

Name of the Database: EconLIT (OVID)

Date of search: 27/12/2022

Econlit <1886 to December 15, 2022>

| # | Query |
| --- | --- |
| 1 | Pneumococc*.mp. |
| 2 | IPD.ti,ab. |
| 3 | (Pneumococ* adj3 Vaccin*).mp. |
| 4 | (Pnu-Imune adj3 Vaccine*).mp. |
| 5 | (PnuImune adj3 Vaccine*).mp. |
| 6 | Pneumovax.mp. |
| 7 | PCV-10.ti,ab. |
| 8 | PCV-13.ti,ab. |
| 9 | PCV-15.ti,ab. |
| 10 | PPV23.ti,ab. |
| 11 | or/1-10 97 |
| 12 | ((Latin adj1 America*) or Latinamerica* or Latinoamerica* or Latin* or Hispanic Americans or Iberoamerica* or (Ibero adj1 Americ*) or Panamerican* or (Central adj1 America*) or Centroamerica* or Mesoamerica* or (Meso adj1 America*) or (Middle adj1 America*) or (South adj1 America*) or Southamerica* or Sudamerica* or (America adj3 Sur) or Caribbean or Caribe* or (West adj1 Indi*) or Antill* or Amerindian* or Indians or (American adj1 Indian*) or (Native* adj1 America*) or Patagoni* or Andes or Andean* or Amazon* or Argentin* or Bolivia* or Brazil* or Brasil* Colombia* or Colombia* or Colombia or Chile* or Ecuador* or Guiana* or Guyan* or Guyan* or Paraguay* or Paraguay* or Peru* or Surinam* or Surinam* or Uruguay* or Venez* or Belize* or Costa Ric* or Costarric* or Costaric* or (Costa adj1 Ric*) or Costarric* or Salvador* or Salvador* or Salvador or Guatemal* or Guatemal* or Guatemala or Hondur* or Nicaragu* Panam* or Mexic* or Cuba* or Dominic* or Dominic* or Haiti* or Jamaic* or Puerto Ric* or Puertorric* or Puertoric*).ti,ab. |
| 13 | 11 and 12 |

Name of the Database: CINAHL (EBSCO)

Date of search: 27/12/2022

| # | Query |
| --- | --- |
| S18 | S14 AND S17 Limiters - Published Date: 20000101-20221231 |
| S17 | S15 OR S16 |
| S16 | AB (Latin N1 America*) OR Latinamerica* OR Latinoamerica* OR Latin* OR Hispanic Americans OR Iberoamerica* OR (Ibero N1 Americ*) OR Panamerican* OR (Central N1 America*) OR Centroamerica* OR Mesoamerica* OR (Meso N1 America*) OR (Middle N1 America*) OR (South N1 America*) OR Southamerica* OR Sudamerica* OR (America N1 Sur) OR Caribbean OR Caribe* OR (West N1 Indi*) OR Antill* OR Amerindian* OR Indians OR (American N1 Indian*) OR (Native N1 America*) OR Patagoni* OR Andes OR Andean* OR Amazon* OR Argentin* OR Bolivia* OR Brazil* OR Brasil* Colombia* OR Colombia* OR Colombia OR Chile* OR Ecuador* OR Guiana* OR Guyan* OR Guyan* OR Paraguay* OR Paraguay* OR Peru* OR Surinam* OR Surinam* OR Uruguay* OR Venez* OR Belize* OR (Costa N1 Ric*) OR Costarric* OR Costaric* OR Costa Ric* OR Costarric* OR Salvador* OR Salvador* OR Guatemal* OR Guatemal* OR Guatemala OR Hondur* OR Nicaragu* OR Panam* OR Mexic* OR Cuba* OR Dominic* OR Dominic* OR Haiti* OR Jamaic* OR (Puerto N1 Ric*) OR Puertorric* OR Puertoric* |
| S15 | TI (Latin N1 America*) OR Latinamerica* OR Latinoamerica* OR Latin* OR Hispanic Americans OR Iberoamerica* OR (Ibero N1 Americ*) OR Panamerican* OR (Central N1 America*) OR Centroamerica* OR Mesoamerica* OR (Meso N1 America*) OR (Middle N1 America*) OR (South N1 America*) OR Southamerica* OR Sudamerica* OR (America N1 Sur) OR Caribbean OR Caribe* OR (West N1 Indi*) OR Antill* OR Amerindian* OR Indians OR (American N1 Indian*) OR (Native N1 America*) OR Patagoni* OR Andes OR Andean* OR Amazon* OR Argentin* OR Bolivia* OR Brazil* OR Brasil* Colombia* OR Colombia* OR Colombia OR Chile* OR Ecuador* OR Guiana* OR Guyan* OR Guyan* OR Paraguay* OR Paraguay* OR Peru* OR Surinam* OR Surinam* OR Uruguay* OR Venez* OR Belize* OR (Costa N1 Ric*) OR Costarric* OR Costaric* OR Costa Ric* OR Costarric* OR Salvador* OR Salvador* OR Guatemal* OR Guatemal* OR Guatemala OR Hondur* OR Nicaragu* OR Panam* OR Mexic* OR Cuba* OR Dominic* OR Dominic* OR Haiti* OR Jamaic* OR (Puerto N1 Ric*) OR Puertorric* OR Puertoric* |
| S14 | S1 OR S2 OR S3 OR S4 OR S5 OR S6 OR S7 OR S8 OR S9 OR S10 OR S11 OR S12 OR S13 |
| S13 | TI PPV23 OR AB PPV23 |
| S12 | TI PCV-15 OR AB PCV-15 |
| S11 | TI PCV-13 OR AB PCV-13 |
| S10 | TI PCV-10 OR AB PCV-10 |
| S9 | TI Pneumovax OR AB Pneumovax |
| S8 | TI (PnuImune N1 Vaccin*) OR AB (PnuImune N1 Vaccin*) |
| S7 | TI (Pnu-Imune N1 Vaccin*) OR AB (Pnu-Imune N1 Vaccin*) |
| S6 | TI (Pneumococcal N1 Vaccin*) OR AB (Pneumococcal N1 Vaccin*) |
| S5 | (MH "Pneumococcal Vaccine") |
| S4 | TI IPD OR AB IPD |
| S3 | TI Pneumococc* OR AB Pneumococc* |
| S2 | (MH "Pneumonia, Bacterial+") |
| S1 | (MH "Pneumococcal Infections+") |

Name of the Database: Global Health (OVID)

Date of search: 27/12/2022

Global Health <1910 to 2022 Week 51>

| # | Query |
| --- | --- |
| 1 | exp Streptococcus pneumoniae/ |
| 2 | Pneumococc*.ti,ab. |
| 3 | IPD.ti,ab. |
| 4 | (Pnu-Imune adj3 Vaccine*).ti,ab. |
| 5 | (PnuImune adj3 Vaccine*).ti,ab. |
| 6 | Pneumovax.ti,ab. |
| 7 | PCV-10.ti,ab. |
| 8 | PCV-13.ti,ab. |
| 9 | PCV-15.ti,ab. |
| 10 | PPV23.ti,ab. |
| 11 | or/1-10 23259 |
| 12 | exp Latin America/ or exp South America/ or exp Central America/ or (Latin adj1 America*).ti,ab. or Latinamerica*.ti,ab. or Latinoamerica*.ti,ab. or Hispanoamerica.ti,ab. or Iberoamerica*.ti,ab. or (Ibero adj1 Americ*).ti,ab. or Panamerica*.ti,ab. or (South adj1 America*).ti,ab. or Southamerica*.ti,ab. or Sudamerica*.ti,ab. or (America adj1 Sur).ti,ab. or (Central adj1 America*).ti,ab. or Centroamerica*.ti,ab. or Mesoamerica*.ti,ab. or (Meso adj1 America*).ti,ab. or (Middle adj1 America*).ti,ab. or exp Caribbean Islands/ or Caribbean*.ti,ab. or Caribe*.ti,ab. or (West adj1 Indi*).ti,ab. or Antill*.ti,ab. or exp American indian/ or Amerindian*.ti,ab. or Indians.ti,ab. or (Native adj1 America*).ti,ab. or Patagoni*.ti,ab. or Andes.ti,ab. or Andean*.ti,ab. or Amazon*.ti,ab. or exp Argentina/ or Argentin*.ti,ab. or exp Bolivia/ or Bolivia*.ti,ab. or exp Brazil/ or Brazil*.ti,ab. or Brasil*.ti,ab. or exp Colombia/ or Colombia*.ti,ab. or exp Chile/ or Chile*.ti,ab. or exp Ecuador/ or Ecuador*.ti,ab. or exp French Guiana/ or Guiana*.ti,ab. or exp Guyana/ or Guyan*.ti,ab. or exp Paraguay/ or Paraguay*.ti,ab. or exp Peru/ or Peru*.ti,ab. or exp Suriname/ or Surinam*.ti,ab. or exp Uruguay/ or Uruguay*.ti,ab. or exp Venezuela/ or Venez*.ti,ab. or exp Belize/ or Beliz*.ti,ab. or exp Costa Rica/ or (Costa adj1 Rica).ti,ab. or Costarric*.ti,ab. or Costaric*.ti,ab. or exp El salvador/ or Salvador*.ti,ab. or exp Guatemala/ or Guatemal*.ti,ab. or exp Honduras/ or Hondur*.ti,ab. or exp Nicaragua/ or Nicaragu*.ti,ab. or exp Panama/ or Panam*.ti,ab. or exp Mexico/ or Mexic*.ti,ab. or exp Cuba/ or Cuba*.ti,ab. or exp Dominican Republic/ or Dominica*.ti,ab. or exp Haiti/ or Haiti*.ti,ab. or exp Jamaica/ or Jamaic*.ti,ab. or exp Puerto Rico/ or (Puerto adj1 Ric*).ti,ab. or Puertoric*.ti,ab. or Puertorric*.ti,ab. |
| 13 | 11 and 12 |

Name of the Database: LILACS (BVS Eng)

Date of search: 27/12/2022

| Database: | LILACS |
| --- | --- |
| Search on: | (MH Pneumococcal Infections OR MH Streptococcus Pneumoniae OR Pneumococ$ OR Neumococ$ OR IPD OR ENI OR MH Pneumococcal Vaccines O Pnu-Imune OR PnuImune OR Pneumovax OR PCV-10 OR PCV-13 OR PCV-15 OR PPV23) [Words] and 2000 OR 2001 OR 2002 OR 2003 OR 2004 OR 2005 OR 2006 OR 2007 OR 2008 OR 2009 OR 2010 OR 2011 OR 2012 OR 2013 OR 2014 OR 2015 OR 2016 OR 2017 OR 2018 OR 2019 OR 2020 OR 2021 OR 2022 [Country, year publication] |

Name of the Database: Web of Science

Date of search: 27/12/2022

| Web of Science Core Collection for: |
| --- |
| (TS=Pneumococcal Infections OR TS=Streptococcus Pneumoniae OR TI=Pneumococc* OR AB= Pneumococc* OR TI=IPD OR AB=IPD OR TS=Pneumococcal Vaccines OR TI=Pnu-Imune OR AB= Pnu-Imune OR TI=PnuImune OR AB=PnuImune OR TI=Pneumovax OR AB=Pneumovax OR TI=PCV-10 OR AB=PCV-10 OR TI=PCV-13 OR AB=PCV-13 OR TI=PCV 15 OR AB=PCV 15 OR TI=PPV23 OR AB=PPV23) AND (TS=Latin America OR TI=(Latin NEAR/1 America*) OR AB=(Latin NEAR/1 America*) OR ALL=Latinamerica* OR ALL=Latinoamerica* OR ALL=Hispanoamerica* OR ALL=Iberoamerica* OR TI=(Ibero NEAR/1 America*) OR AB=(Ibero NEAR/1 America*) OR ALL=Panamerican* OR TS=Central America OR TI=(Central NEAR/1 America*) OR AB=(Central NEAR/1 America*) OR ALL=Centroamerica* OR ALL=Mesoamerica* OR TI=(Meso NEAR/1 America*) OR AB=(Meso NEAR/1 America*) OR TI=(Middle NEAR/1 America*) OR AB=(Middle NEAR/1 America*) OR TS=South America OR TI=(South NEAR/1 America*) OR AB=(South NEAR/1 America*) OR ALL=Southamerica* OR ALL=Sudamerica* OR TI=(America NEAR/1 Sur) OR AB=(America NEAR/1 Sur) OR TS=Caribbean Region OR ALL=Caribbean OR ALL=Caribe* OR TS=West Indies OR TI=(West NEAR/1 Indi*) OR AB=(West NEAR/1 Indi*) OR ALL=Antill* OR TS=Indians, South American OR TS=Indians, Central American OR ALL=Amerindian* OR TI=(America* NEAR/3 Indian*) OR AB=(America* NEAR/3 Indian*) OR TI=(Native NEAR/1 America*) OR AB=(Native NEAR/1 America*) OR ALL=Patagoni* OR ALL=Andes OR ALL=Andean* OR ALL=Amazon* OR ALL=Anguill* OR TI=(Antigua NEAR/1 Barbuda) OR AB=(Antigua NEAR/1 Barbuda) OR ALL=Argentin* OR ALL=Baham* OR ALL=Bermud* OR ALL=Bolivia* OR ALL=Brazil* OR ALL=Brasil* OR ALL=Cayman* OR ALL=Curaçao OR ALL=Colombia* OR ALL=Chile* OR ALL= Ecuador* OR ALL=Grenad* OR ALL=Guadeloup* OR ALL=Guiana* OR ALL=Guyan* OR ALL=Paraguay* OR ALL=Peru* OR ALL=Surinam* OR ALL=Uruguay* OR ALL=Venez* OR ALL=Belize* OR TI=(Costa NEAR/1 Ric*) OR AB=(Costa NEAR/1 Ric*) OR ALL=Costarric* OR ALL=Costaric* OR ALL=Salvador* OR ALL=Guatemal* OR ALL=Hondur* OR ALL=Martiniqu* OR ALL=Nicaragu* OR ALL=Panam* OR TS=Mexico OR ALL=Mexic* OR ALL=Montserrat* OR ALL=Cuba* OR ALL=Dominic* OR ALL=Haiti* OR ALL=Jamaic* OR TS=Puerto Rico OR TI=(Puerto NEAR/1 Ric*) OR AB=(Puerto NEAR/1 Ric*) OR ALL=Puertorric* OR TI=(Saint NEAR/1 Kitts) OR AB=(Saint NEAR/1 Kitts) OR TI=(Trinidad NEAR/1 Tobago) OR AB=(Trinidad NEAR/1 Tobago)) and 2000 or 2001 or 2002 or 2003 or 2004 or 2005 or 2023 or 2022 or 2021 or 2020 or 2019 or 2018 or 2017 or 2016 or 2015 or 2014 or 2013 or 2012 (Publication Years) |

# Supplementary Table 2. List of excluded studies at full text screening stage

| **Author, year** | **Reason for exclusion** |
| --- | --- |
| Agudelo 2005 | Duplicate |
| Alves Cardozo 2014 | Duplicate |
| Bautista Marquez 2013 | Wrong patient population |
| Davalos 2016 | Duplicate |
| Di Fabio 2001 | Duplicate |
| dos Santos 2011 | Duplicate |
| Gagetti 2018 | Duplicate |
| Gomez Barreto 2000 | Duplicate |
| Grenon 2014 | Duplicate |
| Guevara Duncan 2008 | Wrong patient population |
| Hidalgo 2011 | Duplicate |
| Inostroza 2007 | Wrong outcome |
| Ko 2000 | Duplicate |
| Mantese 2003 | Duplicate |
| Moreno 2004 | Wrong outcome |
| Leal Castro 2019 | Duplicate |
| Lovera 2005 | Duplicate |
| Pérez 2013 | Duplicate |
| Pinheiro 2012 | Wrong patient population |
| Pinto 2016 | Duplicate |
| Pírez García 2011 | Duplicate |
| Soto Nogueron 2016 | Wrong patient population |
| Soto Nogueron 2018 | Wrong patient population |
| Torres Cardozo 2017 | Wrong patient population |
| Yoshioka 2012 | Duplicate |

# Supplementary Table 3. Characteristics of included studies

| **Author, year** | **Country** | **Study start date dd/mm/yyyy** | **Study ending date dd/mm/yyyy** | **Study design** | **Age range** | **Sample size** | **Type of IPD^+^** |
| --- | --- | --- | --- | --- | --- | --- | --- |
| Abate 2014* [(1)](https://paperpile.com/c/sdFrri/eLUh) | Argentina | 01/01/1993 | 31/12/2011 | Cross sectional | <18y | 537 | IPD |
| Altclas 2004* [(2)](https://paperpile.com/c/sdFrri/8vUP) | Argentina | 01/01/1993 | 30/06/1998 | Cross sectional | All ages | 107 | Bacteremia |
| Bakir 2003* [(3)](https://paperpile.com/c/sdFrri/Gt8N) | Argentina | 01/01/1993 | 31/12/1999 | Cross sectional | <18y | 274 | IPD |
| Benitez 2017* [(4)](https://paperpile.com/c/sdFrri/G7jP) | Argentina | 01/05/2013 | 30/04/2014 | Cross sectional | <14y | 23 | IPD |
| Berberian 2014* [(5)](https://paperpile.com/c/sdFrri/7hFe) | Argentina | 01/01/1999 | 31/12/2010 | Cross sectional | <18y | 111 | Meningitis |
| Fonaroff 2014* [(6)](https://paperpile.com/c/sdFrri/NKtC) | Argentina | 01/01/2004 | 31/12/2010 | Case series | ≥15y | 93 | Pneumonia |
| Gagetti 2017* [(7)](https://paperpile.com/c/sdFrri/JY3N) | Argentina | 01/01/1993 | 31/12/2014 | Cross sectional/Surveillance | <5y | 4391 | IPD |
| Gagetti 2021* [(8)](https://paperpile.com/c/sdFrri/fKg5) | Argentina | 01/01/1998 | 31/12/2013 | Cross sectional/Surveillance | <5y | 1713 | IPD |
| Gentile 2003* [(9)](https://paperpile.com/c/sdFrri/tzWi) | Argentina | 01/01/1995 | 30/12/2000 | Case series | ≥18y | 101 | Pneumonia |
| Gentile 2018a* [(10)](https://paperpile.com/c/sdFrri/lekQ) | Argentina | 01/01/2007 | 31/12/2014 | Cross sectional/Surveillance | <18y | 297 | Pneumonia |
| Gentile 2018b* [(11)](https://paperpile.com/c/sdFrri/94Yh) | Argentina | 01/01/2012 | 31/12/2017 | Cross sectional | <18y | 135 | Pneumonia |
| Grenón 2005* [(12)](https://paperpile.com/c/sdFrri/ZVxL) | Argentina | 01/06/1998 | 30/06/2001 | Cross sectional | <14y | 101 | IPD |
| Grenón 2014* [(13)](https://paperpile.com/c/sdFrri/kOSm) | Argentina | 01/01/1994 | 31/12/2009 | Case series | <14y | 167 | Meningitis |
| Mathurin 2008* [(14)](https://paperpile.com/c/sdFrri/Gx5y) | Argentina | 01/07/2004 | 31/12/2007 | Prospective cohort | ≥18y | 64 | Bacteriemia |
| Mayoral 2008* [(15)](https://paperpile.com/c/sdFrri/UofQ) | Argentina | 01/01/2003 | 31/12/2005 | Case series | <5y | 76 | IPD |
| Paganini 2001* [(16)](https://paperpile.com/c/sdFrri/kjnw) | Argentina | 01/01/1996 | 31/12/1998 | Cross sectional | <18y | 109 | Pneumonia |
| Pérez 2014* [(17)](https://paperpile.com/c/sdFrri/TMwc) | Argentina | 01/10/2008 | 30/09/2013 | Case series | <18t | 171 | Bacteremia |
| Ruvinsky 2010* [(18)](https://paperpile.com/c/sdFrri/9ctC) | Argentina | 01/01/1994 | 31/12/2007 | Cross sectional/Surveillance | <5y | 2205 | IPD |
| Zintgraff 2022* [(19)](https://paperpile.com/c/sdFrri/eMKK) | Argentina | 01/01/2006 | 31/12/2019 | Cross sectional/Surveillance | <5y | 2908 | IPD |
| Alvares 2011* [(20)](https://paperpile.com/c/sdFrri/U8Hu) | Brazil | 01/04/1999 | 30/04/2009 | Case series | All ages | 72 | Meningitis |
| Azevedo 2016* [(21)](https://paperpile.com/c/sdFrri/ElfU) | Brazil | 01/01/2008 | 31/12/2012 | Cross sectional | All ages | 148 | Meningitis |
| Barroso 2012* [(22)](https://paperpile.com/c/sdFrri/cMq2) | Brazil | 01/01/2000 | 31/12/2008 | Cross sectional/Surveillance | All ages | 1272 | Meningitis |
| Bedran 2005* [(23)](https://paperpile.com/c/sdFrri/HesK) | Brazil | 01/01/1997 | 31/12/2004 | Cross sectional | All ages | 502 | IPD |
| Berezin 2002* [(24)](https://paperpile.com/c/sdFrri/n8eS) | Brazil | 01/01/1994 | 31/12/1999 | Case series | <18y | 55 | Meningitis |
| Berezin 2007* [(25)](https://paperpile.com/c/sdFrri/eojm) | Brazil | 01/06/1997 | 31/05/2001 | Case series | <5y | 625 | IPD |
| Berezin 2020* [(26)](https://paperpile.com/c/sdFrri/hkT3) | Brazil | 01/01/2005 | 31/12/2015 | Case series | <18y | 260 | IPD |
| Brandileone 2006* [(27)](https://paperpile.com/c/sdFrri/Gjpx) | Brazil | 01/01/1993 | 31/12/2004 | Cross sectional/Surveillance | All ages | 6470 | IPD |
| Brandileone 2021* [(28)](https://paperpile.com/c/sdFrri/YLC4) | Brazil | 01/01/2007 | 31/12/2019 | Cross sectional/Surveillance | All ages | 11380 | IPD |
| Caierao 2014* [(29)](https://paperpile.com/c/sdFrri/Xaj2) | Brazil | 01/01/2007 | 31/12/2012 | Cross sectional | All ages | 325 | IPD |
| Cassiolato 2019*** [(30)](https://paperpile.com/c/sdFrri/nNa6) | Brazil | 01/01/2005 | 31/12/2017 | Cross sectional | All ages | 9854 | IPD |
| Cazentini Medeiros 2017* [(31)](https://paperpile.com/c/sdFrri/9OrS) | Brazil | 01/01/1998 | 31/12/2013 | Cross sectional/Surveillance | All ages | 796 | IPD |
| Christophe 2018* [(32)](https://paperpile.com/c/sdFrri/2zYg) | Brazil | 01/01/2013 | 01/06/2015 | Cross sectional | >50y | 102 | IPD |
| da Silva 2010* [(33)](https://paperpile.com/c/sdFrri/rr9C) | Brazil | 01/01/2005 | 31/12/2008 | Case series | All ages | 168 | Meningitis |
| dos Santos 2013* [(34)](https://paperpile.com/c/sdFrri/upvW) | Brazil | 01/01/2006 | 30/09/2012 | Case series | All ages | 259 | IPD |
| Gomes de Oliveira Magalhaes 2003* [(35)](https://paperpile.com/c/sdFrri/h45Z) | Brazil | 01/06/2000 | 30/05/2001 | Cross sectional/Surveillance | <5y | 31 | IPD |
| Gouveia 2011* [(36)](https://paperpile.com/c/sdFrri/Y73D) | Brazil | 01/12/1995 | 30/11/2005 | Case series | All ages | 548 | Meningitis |
| Laval 2006* [(37)](https://paperpile.com/c/sdFrri/Ihlr) | Brazil | 01/05/2000 | 31/08/2001 | Cross sectional/Surveillance | <5y | 773 | IPD |
| Levin 2003* [(38)](https://paperpile.com/c/sdFrri/NDoO) | Brazil | 01/07/1991 | 31/12/1994 | Cross sectional | All ages | 165 | IPD |
| Mantese 2009* [(39)](https://paperpile.com/c/sdFrri/9azR) | Brazil | 01/04/1999 | 31/12/2008 | Case series | <5y | 142 | IPD |
| Menezes 2011* [(40)](https://paperpile.com/c/sdFrri/iXfi) | Brazil | 01/01/2000 | 31/12/2007 | Cross sectional/Surveillance | All ages | 421 | Meningitis |
| Mott 2014* [(41)](https://paperpile.com/c/sdFrri/KoBe) | Brazil | 01/01/2010 | 30/04/2012 | Cross sectional/Surveillance | All ages | 159 | IPD |
| Nascimento-Carvalho 2003* [(42)](https://paperpile.com/c/sdFrri/CG71) | Brazil | 01/09/1997 | 31/05/2002 | Cross sectional/Surveillance | <18y | 70 | IPD |
| Neves Reis 2002* [(43)](https://paperpile.com/c/sdFrri/vjNv) | Brazil | 01/12/1995 | 30/11/1999 | Case series | All ages | 305 | Meningitis |
| Pinto 2019* [(44)](https://paperpile.com/c/sdFrri/FVou) | Brazil | 01/01/1990 | 31/12/2014 | Cross sectional | All ages | 783 | IPD |
| Rocha Dullius 2018* [(45)](https://paperpile.com/c/sdFrri/RYx2) | Brazil | 01/01/2005 | 31/12/2016 | Cross sectional | NR | 118 | IPD |
| Rossoni 2008* [(46)](https://paperpile.com/c/sdFrri/7KDd) | Brazil | 01/04/2001 | 30/08/2002 | Cross sectional | All ages | 436 | Meningitis |
| Soares dos Santos 2022* [(47)](https://paperpile.com/c/sdFrri/aRUw) | Brazil | 01/01/1996 | 31/12/2012 | Cross sectional/Surveillance | NR | 917 | Meningitis |
| Vieira 2007* [(48)](https://paperpile.com/c/sdFrri/LHye) | Brazil | 01/01/1995 | 31/12/2004 | Case series | All ages | 232 | Meningitis |
| Yoshioka 2011* [(49)](https://paperpile.com/c/sdFrri/C2P5) | Brazil | 01/01/2003 | 30/10/2008 | Cross sectional | <18y | 107 | Pneumonia |
| Abarca 2008* [(50)](https://paperpile.com/c/sdFrri/MGha) | Chile | 01/05/2001 | 30/04/2002 | Cross sectional/Surveillance | <2y | 4369 | IPD |
| Aguilera 2010* [(51)](https://paperpile.com/c/sdFrri/6k4n) | Chile | 01/01/2005 | 30/08/2006 | Case series | ≥18y | 56 | Bacteriemia |
| Contreras 2002* [(52)](https://paperpile.com/c/sdFrri/Ovfi) | Chile | 01/04/1994 | 30/05/1999 | Cross sectional | <18y | 78 | IPD |
| Fica 2014* [(53)](https://paperpile.com/c/sdFrri/9TIL) | Chile | 01/01/2005 | 31/12/2010 | Case series | ≥18y | 59 | Pneumonia |
| Rioseco 2004* [(54)](https://paperpile.com/c/sdFrri/WHcR) | Chile | 01/01/1997 | 31/08/2002 | Case series | ≥18y | 45 | Pneumonia |
| Rioseco 2018* [(55)](https://paperpile.com/c/sdFrri/akuk) | Chile | 01/01/2010 | 31/12/2014 | Case series | ≥18y | 70 | Pneumonia |
| Saldías 2011* [(56)](https://paperpile.com/c/sdFrri/rzbx) | Chile | 01/01/2002 | 31/12/2005 | Case series | ≥18y | 151 | Pneumonia |
| Agudelo 2006* [(57)](https://paperpile.com/c/sdFrri/pgXM) | Colombia | 01/01/1994 | 31/12/2004 | Cross sectional/Surveillance | All ages | 2022 | IPD |
| Camacho Moreno 2020* [(58)](https://paperpile.com/c/sdFrri/aLvN) | Colombia | 01/01/2008 | 31/12/2017 | Case series | <18y | 463 | IPD |
| Farfán-Albarracín 2022* [(59)](https://paperpile.com/c/sdFrri/I8ys) | Colombia | 01/01/2008 | 31/12/2019 | Case series | <18y | 81 | Meningitis |
| Gutierrez-Tobar 2022* [(60)](https://paperpile.com/c/sdFrri/nfFh) | Colombia | 01/01/2008 | 31/12/2019 | Cross sectional/Surveillance | <18y | 566 | Pneumonia |
| Leal Castro 2022* [(61)](https://paperpile.com/c/sdFrri/lbs2) | Colombia | 01/01/2011 | 31/12/2017 | Case series | ≥18y | 169 | IPD |
| Parra 2014* [(62)](https://paperpile.com/c/sdFrri/XHNB) | Colombia | 01/01/2005 | 31/12/2010 | Cross sectional/Surveillance | All ages | 1775 | IPD |
| Parra 2017* [(63)](https://paperpile.com/c/sdFrri/e0qM) | Colombia | 01/01/1994 | 31/12/2013 | Cross sectional/Surveillance | All ages | 4991 | IPD |
| Vela 2001* [(64)](https://paperpile.com/c/sdFrri/xhhk) | Colombia | 01/02/1994 | 31/12/1999 | Cross sectional/Surveillance | <5y | 660 | IPD |
| Barboza 2018** [(65)](https://paperpile.com/c/sdFrri/AGZP) | Costa Rica | 01/01/2009 | 31/12/2015 | Cross sectional | <2y | 76 | Meningitis |
| Ulloa-Gutierrez 2003* [(66)](https://paperpile.com/c/sdFrri/1qjo) | Costa Rica | 01/01/1995 | 31/12/2001 | Case series | <14y | 132 | IPD |
| Vargas-Gutierrez 2015** [(67)](https://paperpile.com/c/sdFrri/IG1I) | Costa Rica | 01/01/2006 | 31/01/2014 | Case series | <14y | 121 | Pneumonia |
| Fonseca Hernández 2017* [(68)](https://paperpile.com/c/sdFrri/Isga) | Cuba | 01/01/2014 | 31/03/2016 | Case series | <5y | 37 | IPD |
| Toraño-Peraza 2014* [(69)](https://paperpile.com/c/sdFrri/89UW) | Cuba | 01/01/2007 | 31/12/2012 | Case series | All ages | 237 | Meningitis |
| Elenga 2015* [(70)](https://paperpile.com/c/sdFrri/eFrI) | French Guiana | 01/01/2000 | 31/12/2010 | Case series | <18y | 60 | Meningitis |
| Trotman 2009* [(71)](https://paperpile.com/c/sdFrri/vogd) | Jamaica | 01/01/1995 | 31/12/1999 | Case series | <5y | 25 | Meningitis |
| Alves Cardozo 2008* [(72)](https://paperpile.com/c/sdFrri/D9UG) | LAC | 01/07/1998 | 31/12/2002 | Cross sectional | <5y | 240 | Pneumonia |
| Hortal 2000b* [(73)](https://paperpile.com/c/sdFrri/PyRD) | LAC | 01/01/1993 | 20/09/1999 | Cross sectional/Surveillance | <5y | 3393 | Pneumonia |
| Hortal 2001* [(74)](https://paperpile.com/c/sdFrri/66kj) | LAC | 01/01/1993 | 30/06/1999 | Cross sectional/Surveillance | <5y | 4105 | IPD |
| Moreno 2020* [(75)](https://paperpile.com/c/sdFrri/j7Oi) | LAC | 01/01/2000 | 31/12/2015 | Cross sectional/Surveillance | All ages | 185 | IPD |
| Arredondo-García 2011* [(76)](https://paperpile.com/c/sdFrri/frXo) | Mexico | 01/02/2002 | 31/12/2005 | Cross sectional | <18y | 150 | IPD |
| Echaniz-Aviles 2019* [(77)](https://paperpile.com/c/sdFrri/GrrN) | Mexico | 01/01/2000 | 30/09/2015 | Case series | ≥18y | 96 | Meningitis |
| Gómez-Barreto 2010* [(78)](https://paperpile.com/c/sdFrri/EeOB) | Mexico | 01/01/1997 | 31/08/2014 | Case series | <14y | 156 | IPD |
| Villaseñor-Sierra 2008* [(79)](https://paperpile.com/c/sdFrri/lBQm) | Mexico | 01/03/2000 | 30/05/2005 | Cross sectional | <18y | 98 | Meningitis |
| De León 2011* [(80)](https://paperpile.com/c/sdFrri/chOl) | Panama | 01/01/2010 | 30/06/2011 | Case series | <18y | 23 | IPD |
| Aranda 2014* [(81)](https://paperpile.com/c/sdFrri/3kbU) | Paraguay | 01/01/1993 | 30/06/2006 | Cross sectional | <18y | 394 | Meningitis |
| León 2020* [(82)](https://paperpile.com/c/sdFrri/Jc6k) | Paraguay | 01/01/2010 | 31/12/2018 | Cross sectional/Surveillance | All ages | 793 | IPD |
| Lovera 2011* [(83)](https://paperpile.com/c/sdFrri/hzFG) | Paraguay | 01/01/2000 | 30/04/2010 | Case series | <18y | 46 | Meningitis |
| Sanabria 2009* [(84)](https://paperpile.com/c/sdFrri/15Ol) | Paraguay | 01/01/2002 | 30/08/2007 | Cross sectional | <18y | 78 | IPD |
| Castro 2017* [(85)](https://paperpile.com/c/sdFrri/OFeg) | Peru | 01/06/2009 | 30/06/2011 | Case series | ≥18y | 43 | IPD |
| Castillo-Tokumori 2018** [(86)](https://paperpile.com/c/sdFrri/NgkE) | Peru | 01/11/2016 | 28/0272018 | Case series | <5y | 45 | IPD |
| Hawkins 2017* [(87)](https://paperpile.com/c/sdFrri/aTYC) | Peru | 01/01/2006 | 31/12/2011 | Cross sectional/Surveillance | NR | 212 | IPD |
| Luna-Muschi 2019* [(88)](https://paperpile.com/c/sdFrri/pHCt) | Peru | 01/01/2006 | 31/12/2011 | Cross sectional/Surveillance | <18y | 159 | IPD |
| Morales de Santa Gadea 2003* [(89)](https://paperpile.com/c/sdFrri/zvqc) | Peru | 01/10/2000 | 31/12/2001 | Cross sectional | <5y | 1283 | IPD |
| Rivera-Matos 2005* [(90)](https://paperpile.com/c/sdFrri/raOq) | Puerto Rico | 01/01/2001 | 31/12/2001 | Cross sectional/Surveillance | All ages | 192 | IPD |
| Nurse Lucas 2016* [(91)](https://paperpile.com/c/sdFrri/Iijy) | Trinidad and Tobago | 01/01/1997 | 31/12/2013 | Cross sectional/Surveillance | All ages | 83 | IPD |
| Assandri 2015* [(92)](https://paperpile.com/c/sdFrri/o0Uc) | Uruguay | 01/01/2001 | 31/12/2013 | Case series | <1y | 25 | IPD |
| Camou 2003* [(93)](https://paperpile.com/c/sdFrri/EmIJ) | Uruguay | 01/01/1994 | 31/12/2001 | Cross sectional/Surveillance | <5y | 506 | IPD |
| Ferrari Castilla 2007* [(94)](https://paperpile.com/c/sdFrri/c1OH) | Uruguay | 01/01/1998 | 31/12/2004 | Cross sectional/Surveillance | <18y | 512 | Pneumonia |
| Hortal 2000a* [(95)](https://paperpile.com/c/sdFrri/JM4M) | Uruguay | 01/01/1987 | 31/12/1997 | Cross sectional/Surveillance | >5y | 228 | IPD |
| Hortal 2007* [(96)](https://paperpile.com/c/sdFrri/0NoP) | Uruguay | 01/01/2001 | 31/05/2004 | Cross sectional/Surveillance | <5y | 2034 | Pneumonia |
| Hortal 2008* [(97)](https://paperpile.com/c/sdFrri/XtOj) | Uruguay | 01/06/2000 | 31/12/2004 | Case series | <14y | 410 | Pneumonia |
| Machado 2014* [(98)](https://paperpile.com/c/sdFrri/2DrY) | Uruguay | 01/01/2010 | 31/12/2010 | Case series | <18y | 43 | Pneumonia |
| Machado 2020* [(99)](https://paperpile.com/c/sdFrri/3som) | Uruguay | 01/01/2009 | 31/12/2018 | Case series | <14y | 197 | Pneumonia |
| Pirez 2001* [(100)](https://paperpile.com/c/sdFrri/VEoM) | Uruguay | 01/09/1997 | 31/08/1998 | Case series | <5y | 1082 | Pneumonia |
| Pirez 2014* [(101)](https://paperpile.com/c/sdFrri/YFik) | Uruguay | 01/01/2003 | 31/12/2012 | Non-comparative cohort | <18y | 630 | Pneumonia |
| Pirez 2017* [(102)](https://paperpile.com/c/sdFrri/pNN0) | Uruguay | 01/01/2005 | 31/12/2014 | Case series | <18y | 171015 | Meningitis |
| Pirez Garcia 2008* [(103)](https://paperpile.com/c/sdFrri/dVUs) | Uruguay | 01/01/1998 | 31/12/2005 | Case series | <2y | 192 | Pneumonia |

NR: Not Reported

**^#^**The references of the included studies are in the Annex A, Supplementary material

*Full text; **Abstract/Poster; ***Thesis

^+^IPD: Invasive Pneumococcal disease

# Risk of bias assessment

## Supplementary Table 4. Risk of bias assessment for cohort and cross sectional studies

| **Author, year** | **Evaluation *** | | | | | | | | | | | | | | |
| --- | --- | --- | --- | --- | --- | --- | --- | --- | --- | --- | --- | --- | --- | --- | --- |
|  | **1** | **2** | **3** | **4** | **5** | **6** | **7** | **8** | **9** | **10** | **11** | **12** | **13** | **14** | **Final** |
| Abarca 2008 [(50)](https://paperpile.com/c/sdFrri/MGha) | Yes | Yes | CD | Yes | No | NA | NA | No | Yes | No | Yes | NA | NA | NA | FAIR |
| Abate 2014 [(1)](https://paperpile.com/c/sdFrri/eLUh) | Yes | Yes | Yes | Yes | No | NA | NA | No | Yes | No | Yes | No | NA | No | FAIR |
| Agudelo 2006 [(57)](https://paperpile.com/c/sdFrri/pgXM) | Yes | Yes | Yes | Yes | No | NA | NA | No | Yes | No | Yes | NA | NA | NA | GOOD |
| Alves Cardozo 2008 [(72)](https://paperpile.com/c/sdFrri/D9UG) | Yes | Yes | CD | Yes | Yes | NA | NA | Yes | Yes | NA | Yes | No | NA | Yes | FAIR |
| Aranda 2014 [(81)](https://paperpile.com/c/sdFrri/3kbU) | Yes | Yes | Yes | Yes | No | NA | NA | NA | Yes | NA | Yes | No | NA | No | FAIR |
| Arredondo-García 2011 [(76)](https://paperpile.com/c/sdFrri/frXo) | Yes | Yes | CD | Yes | No | NA | NA | No | Yes | No | Yes | No | NA | NA | FAIR |
| Altclas 2004 [(2)](https://paperpile.com/c/sdFrri/8vUP) | Yes | Yes | CD | Yes | No | NA | NA | No | Yes | No | Yes | No | NA | No | POOR |
| Azevedo 2016 [(21)](https://paperpile.com/c/sdFrri/ElfU) | Yes | Yes | CD | Yes | No | Yes | Yes | No | Yes | NA | Yes | No | NA | NA | FAIR |
| Bakir 2003 [(3)](https://paperpile.com/c/sdFrri/Gt8N) | Yes | Yes | CD | Yes | No | NA | NA | No | Yes | NA | Yes | No | NA | No | POOR |
| Barboza 2018 [(65)](https://paperpile.com/c/sdFrri/AGZP) | Yes | Yes | No | Yes | No | NA | NA | No | Yes | No | Yes | NA | NA | NA | FAIR |
| Barroso 2012 [(22)](https://paperpile.com/c/sdFrri/cMq2) | Yes | Yes | No | Yes | No | No | NA | NA | Yes | No | Yes | NA | NA | NA | FAIR |
| Bedran 2005 [(23)](https://paperpile.com/c/sdFrri/HesK) | Yes | Yes | CD | Yes | No | NA | NA | No | Yes | No | Yes | No | NA | NA | FAIR |
| Benitez 2017 [(4)](https://paperpile.com/c/sdFrri/G7jP) | Yes | Yes | Yes | Yes | No | NA | NA | No | Yes | No | Yes | No | NA | NA | GOOD |
| Berberian 2014 [(5)](https://paperpile.com/c/sdFrri/7hFe) | Yes | Yes | No | Yes | No | Yes | Yes | No | Yes | No | Yes | No | No | No | GOOD |
| Brandileone 2006 [(27)](https://paperpile.com/c/sdFrri/Gjpx) | Yes | Yes | CD | Yes | No | NA | NA | No | Yes | No | Yes | NA | NA | NA | FAIR |
| Brandileone 2021 [(28)](https://paperpile.com/c/sdFrri/YLC4) | Yes | Yes | Yes | CD | No | No | NA | No | Yes | No | Yes | No | NA | NA | GOOD |
| Caierao 2014 [(29)](https://paperpile.com/c/sdFrri/Xaj2) | Yes | Yes | CD | Yes | No | NA | NA | No | Yes | No | Yes | No | NA | NA | FAIR |
| Camou 2003 [(93)](https://paperpile.com/c/sdFrri/EmIJ) | Yes | Yes | Yes | Yes | No | No | NA | No | Yes | No | Yes | No | NA | NA | GOOD |
| Cassiolato 2019 [(30)](https://paperpile.com/c/sdFrri/nNa6) | Yes | Yes | Yes | Yes | No | NA | NA | No | Yes | No | Yes | No | NA | NA | GOOD |
| Cazentini Medeiros 2017 [(31)](https://paperpile.com/c/sdFrri/9OrS) | Yes | Yes | Yes | Yes | No | No | NA | No | No | NA | No | No | NA | No | FAIR |
| Christophe 2018 [(32)](https://paperpile.com/c/sdFrri/2zYg) | Yes | Yes | CD | Yes | No | NA | NA | No | Yes | No | Yes | No | NA | NA | FAIR |
| Contreras 2002 [(52)](https://paperpile.com/c/sdFrri/Ovfi) | Yes | Yes | No | Yes | No | Yes | Yes | No | Yes | No | Yes | No | No | No | POOR |
| Ferrari Castilla 2007 [(94)](https://paperpile.com/c/sdFrri/c1OH) | Yes | Yes | CD | Yes | No | NA | NA | No | Yes | NA | Yes | NA | NA | NA | FAIR |
| Gagetti 2017 [(7)](https://paperpile.com/c/sdFrri/JY3N) | Yes | Yes | Yes | Yes | No | NA | NA | NA | Yes | NA | Yes | NA | NA | NA | GOOD |
| Gagetti 2021 [(8)](https://paperpile.com/c/sdFrri/fKg5) | Yes | Yes | Yes | Yes | Yes | NA | NA | NA | Yes | No | Yes | NA | NA | NA | FAIR |
| Gentile 2018a [(10)](https://paperpile.com/c/sdFrri/lekQ) | Yes | Yes | Yes | Yes | No | No | NA | Yes | Yes | NA | Yes | No | NA | No | FAIR |
| Gentile 2018b [(11)](https://paperpile.com/c/sdFrri/94Yh) | Yes | Yes | No | Yes | No | No | No | No | Yes | No | Yes | No | No | No | POOR |
| Gomes de Oliveira Magalhães 2003 [(35)](https://paperpile.com/c/sdFrri/h45Z) | Yes | Yes | CD | Yes | No | No | NA | Yes | Yes | NA | Yes | No | NA | No | FAIR |
| Grenon 2005 [(12)](https://paperpile.com/c/sdFrri/ZVxL) | Yes | Yes | CD | Yes | No | NA | NA | Yes | Yes | No | Yes | No | NA | NA | FAIR |
| Gutierrez-Tobar 2022 [(60)](https://paperpile.com/c/sdFrri/nfFh) | Yes | Yes | NA | Yes | No | NA | NA | NA | Yes | No | Yes | NA | NA | NA | GOOD |
| Hawkins 2017 [(87)](https://paperpile.com/c/sdFrri/aTYC) | Yes | No | Yes | Yes | No | NA | NA | No | Yes | No | Yes | No | NA | NA | FAIR |
| Hortal 2000a [(95)](https://paperpile.com/c/sdFrri/JM4M) | Yes | Yes | Yes | Yes | No | NA | NA | No | Yes | No | Yes | NA | NA | NA | FAIR |
| Hortal 2000b [(73)](https://paperpile.com/c/sdFrri/PyRD) | Yes | Yes | Yes | Yes | No | NA | NA | Yes | Yes | No | Yes | No | NA | NA | GOOD |
| Hortal 2001 [(74)](https://paperpile.com/c/sdFrri/66kj) | Yes | Yes | CD | Yes | No | NA | NA | No | Yes | No | Yes | NA | NA | NA | FAIR |
| Hortal 2007 [(96)](https://paperpile.com/c/sdFrri/0NoP) | Yes | Yes | Yes | Yes | No | NA | NA | Yes | Yes | No | Yes | No | NA | NA | GOOD |
| Laval 2006 [(37)](https://paperpile.com/c/sdFrri/Ihlr) | Yes | Yes | CD | Yes | Yes | NA | NA | No | Yes | No | Yes | NA | NA | NA | FAIR |
| León 2020 [(82)](https://paperpile.com/c/sdFrri/Jc6k) | Yes | Yes | CD | Yes | No | No | NA | No | Yes | NA | Yes | No | NA | No | FAIR |
| Levin 2003 [(38)](https://paperpile.com/c/sdFrri/NDoO) | Yes | Yes | CD | Yes | No | Yes | NA | No | Yes | NA | Yes | No | NA | No | FAIR |
| Luna-Muschi 2019 [(88)](https://paperpile.com/c/sdFrri/pHCt) | Yes | Yes | Yes | Yes | No | NA | NA | No | Yes | No | Yes | NA | NA | NA | GOOD |
| Mathurin 2008 [(14)](https://paperpile.com/c/sdFrri/Gx5y) | Yes | Yes | CD | Yes | No | Yes | Yes | No | Yes | No | Yes | No | No | No | POOR |
| Menezes 2011 [(40)](https://paperpile.com/c/sdFrri/iXfi) | Yes | Yes | Yes | Yes | No | No | NA | No | Yes | NA | Yes | NA | NA | NA | FAIR |
| Morales de Santa Gadea 2003 [(89)](https://paperpile.com/c/sdFrri/zvqc) | Yes | Yes | Yes | Yes | No | NA | NA | Yes | Yes | No | Yes | No | NA | NA | GOOD |
| Moreno 2020 [(75)](https://paperpile.com/c/sdFrri/j7Oi) | Yes | Yes | CD | Yes | No | NA | NA | No | Yes | No | Yes | NA | NA | NA | FAIR |
| Mott 2014 [(41)](https://paperpile.com/c/sdFrri/KoBe) | Yes | Yes | CD | Yes | No | NA | NA | No | Yes | No | Yes | NA | NA | NA | FAIR |
| Nascimento-Carvalho 2003 [(42)](https://paperpile.com/c/sdFrri/CG71) | Yes | Yes | CD | Yes | Yes | Yes | NA | No | Yes | No | Yes | No | NA | NA | FAIR |
| Nurse-Lucas 2016 [(91)](https://paperpile.com/c/sdFrri/Iijy) | Yes | Yes | CD | CD | No | No | NA | No | No | No | Yes | No | NA | NA | FAIR |
| Paganini 2001 [(16)](https://paperpile.com/c/sdFrri/kjnw) | Yes | Yes | CD | Yes | No | No | NA | Yes | Yes | NA | Yes | No | NA | No | FAIR |
| Parra 2014 [(62)](https://paperpile.com/c/sdFrri/XHNB) | Yes | Yes | CD | Yes | No | NA | NA | No | Yes | No | Yes | NA | NA | NA | FAIR |
| Parra 2017 [(63)](https://paperpile.com/c/sdFrri/e0qM) | Yes | Yes | CD | Yes | No | NA | NA | No | Yes | No | Yes | NA | NA | NA | FAIR |
| Pinto 2019 [(44)](https://paperpile.com/c/sdFrri/FVou) | Yes | Yes | CD | Yes | No | No | NA | No | Yes | NA | Yes | No | NA | No | FAIR |
| Pirez 2014 [(101)](https://paperpile.com/c/sdFrri/YFik) | Yes | Yes | CD | Yes | No | Yes | Yes | Yes | Yes | No | Yes | No | CD | NA | FAIR |
| Rivera-Matos 2005 [(90)](https://paperpile.com/c/sdFrri/raOq) | Yes | Yes | CD | Yes | No | NA | NA | No | Yes | No | Yes | NA | NA | NA | FAIR |
| Rocha Dullius 2018 [(45)](https://paperpile.com/c/sdFrri/RYx2) | Yes | Yes | CD | Yes | No | NA | NA | NA | Yes | No | Yes | No | NA | NA | FAIR |
| Rossoni 2008 [(46)](https://paperpile.com/c/sdFrri/7KDd) | Yes | Yes | CD | Yes | No | NA | NA | Yes | Yes | No | Yes | No | NA | NA | FAIR |
| Ruvinsky 2010 [(18)](https://paperpile.com/c/sdFrri/9ctC) | Yes | Yes | Yes | Yes | No | NA | NA | No | Yes | No | Yes | NA | NA | NA | GOOD |
| Sanabria 2009 [(84)](https://paperpile.com/c/sdFrri/15Ol) | Yes | Yes | CD | Yes | No | Yes | NA | No | Yes | NA | Yes | No | NA | No | POOR |
| Soares dos Santos 2022 [(47)](https://paperpile.com/c/sdFrri/aRUw) | Yes | Yes | Yes | Yes | No | NA | NA | No | Yes | No | Yes | NA | NA | NA | GOOD |
| Vela 2001 [(64)](https://paperpile.com/c/sdFrri/xhhk) | Yes | Yes | Yes | Yes | No | NA | NA | No | Yes | No | Yes | NA | NA | NA | GOOD |
| Villaseñor-Sierra 2008 [(79)](https://paperpile.com/c/sdFrri/lBQm) | Yes | Yes | CD | Yes | No | NA | NA | No | Yes | No | Yes | No | NA | NA | FAIR |
| Yoshioka 2011 [(49)](https://paperpile.com/c/sdFrri/C2P5) | Yes | Yes | CD | Yes | No | Yes | NA | Yes | Yes | NA | Yes | No | NA | No | FAIR |
| Zintgraff 2022 [(19)](https://paperpile.com/c/sdFrri/eMKK) | Yes | Yes | CD | Yes | No | Yes | No | Yes | Yes | No | Yes | No | CD | No | FAIR |

* **NA**: Not applicable, **CD**: Cannot be determined

1. Was the question or research objective clearly stated in this study?
2. Was the study population clearly specified and defined?
3. Was the participation of eligible people at least 50%?
4. Were all subjects selected or recruited from the same population or from similar populations (including the same time period)? Were the inclusion and exclusion criteria for participation in the study uniformly pre-specified and applied to all participants?
5. Was a sample size justification, power description, or variance and effect estimates provided?
6. For the analysis in this study, were the exposure(s) of interest measured before the outcome(s) were measured?
7. Was the follow-up period sufficient for one to reasonably expect to see an association between exposure and outcome if it existed?
8. For exposures that may vary in quantity or level, did the study examine different levels of exposure in relation to the outcome (e.g., exposure categories or exposure measured as a continuous variable)?
9. Were exposure measures (independent variables) clearly defined, valid, reliable, and consistently implemented across all study participants?
10. Were exposures evaluated more than once over time?
11. Were outcome measures (dependent variables) clearly defined, valid, reliable, and consistently implemented across all study participants?
12. Were outcome evaluators blinded to participants' exposure status?
13. Was the loss of follow-up after study initiation 20% or less?
14. Were potential key confounding variables measured and adjusted statistically for their impact on the relationship between exposure(s) and outcome(s)?

## Supplementary Table 5. Risk of bias assessment for case series studies

| **Author, year** | **Evaluation *** | | | | | | | | | |
| --- | --- | --- | --- | --- | --- | --- | --- | --- | --- | --- |
|  | **1** | **2** | **3** | **4** | **5** | **6** | **7** | **8** | **9** | **Final** |
| Aguilera 2010 [(51)](https://paperpile.com/c/sdFrri/6k4n) | Yes | Yes | CD | Yes | Yes | Yes | Yes | No | Yes | GOOD |
| Alvares 2011 [(20)](https://paperpile.com/c/sdFrri/U8Hu) | Yes | No | CD | Yes | Yes | Yes | Yes | No | Yes | FAIR |
| Assandri 2015 [(92)](https://paperpile.com/c/sdFrri/o0Uc) | Yes | Yes | Yes | Yes | Yes | Yes | Yes | No | Yes | GOOD |
| Berezin 2002 [(24)](https://paperpile.com/c/sdFrri/n8eS) | Yes | Yes | CD | Yes | Yes | Yes | Yes | No | Yes | GOOD |
| Berezin 2007 [(25)](https://paperpile.com/c/sdFrri/eojm) | Yes | No | CD | Yes | Yes | Yes | Yes | No | Yes | FAIR |
| Berezin 2020 [(26)](https://paperpile.com/c/sdFrri/hkT3) | Yes | Yes | CD | Yes | Yes | Yes | Yes | No | Yes | GOOD |
| Camacho Moreno 2020 [(58)](https://paperpile.com/c/sdFrri/aLvN) | Yes | Yes | CD | Yes | Yes | Yes | Yes | No | Yes | GOOD |
| Castillo-Tokumori 2018 [(86)](https://paperpile.com/c/sdFrri/NgkE) | Yes | No | CD | Yes | Yes | Yes | Yes | No | Yes | FAIR |
| Castro 2017 [(85)](https://paperpile.com/c/sdFrri/OFeg) | Yes | Yes | CD | Yes | Yes | Yes | Yes | Yes | Yes | GOOD |
| da Silva 2010 [(33)](https://paperpile.com/c/sdFrri/rr9C) | Yes | Yes | CD | Yes | Yes | Yes | Yes | No | Yes | GOOD |
| De León 2011 [(80)](https://paperpile.com/c/sdFrri/chOl) | Yes | No | Yes | Yes | Yes | Yes | Yes | No | No | FAIR |
| dos Santos 2013 [(34)](https://paperpile.com/c/sdFrri/upvW) | Yes | No | CD | Yes | Yes | Yes | Yes | No | Yes | FAIR |
| Echaniz-Aviles 2019 [(77)](https://paperpile.com/c/sdFrri/GrrN) | Yes | Yes | CD | Yes | Yes | Yes | Yes | Yes | Yes | GOOD |
| Elenga 2015 [(70)](https://paperpile.com/c/sdFrri/eFrI) | Yes | Yes | CD | Yes | Yes | Yes | Yes | No | Yes | FAIR |
| Farfán-Albarracín 2022 [(59)](https://paperpile.com/c/sdFrri/I8ys) | Yes | Yes | CD | Yes | Yes | Yes | Yes | Yes | Yes | GOOD |
| Fica 2014 [(53)](https://paperpile.com/c/sdFrri/9TIL) | Yes | Yes | Yes | Yes | Yes | Yes | Yes | Yes | Yes | GOOD |
| Fonaroff 2014 [(6)](https://paperpile.com/c/sdFrri/NKtC) | Yes | Yes | CD | Yes | Yes | Yes | Yes | Yes | Yes | GOOD |
| Fonseca Hernández 2017 [(68)](https://paperpile.com/c/sdFrri/Isga) | Yes | No | CD | Yes | Yes | Yes | Yes | No | Yes | FAIR |
| Gentile 2003 [(9)](https://paperpile.com/c/sdFrri/tzWi) | Yes | Yes | CD | Yes | Yes | Yes | Yes | Yes | Yes | GOOD |
| Gómez-Barreto 2010 [(78)](https://paperpile.com/c/sdFrri/EeOB) | Yes | Yes | CD | Yes | Yes | Yes | Yes | Yes | Yes | GOOD |
| Gouveia 2011 [(36)](https://paperpile.com/c/sdFrri/Y73D) | Yes | No | Yes | Yes | Yes | Yes | Yes | Yes | Yes | FAIR |
| Grenon 2014 [(13)](https://paperpile.com/c/sdFrri/kOSm) | Yes | Yes | Yes | Yes | Yes | Yes | Yes | Yes | Yes | GOOD |
| Hortal 2008 [(97)](https://paperpile.com/c/sdFrri/XtOj) | Yes | Yes | Yes | Yes | Yes | Yes | Yes | No | Yes | GOOD |
| Leal Castro 2022 [(61)](https://paperpile.com/c/sdFrri/lbs2) | Yes | Yes | CD | Yes | Yes | Yes | Yes | Yes | Yes | GOOD |
| Lovera 2011 [(83)](https://paperpile.com/c/sdFrri/hzFG) | Yes | Yes | Yes | Yes | Yes | Yes | Yes | Yes | Yes | GOOD |
| Machado 2014 [(98)](https://paperpile.com/c/sdFrri/2DrY) | Yes | Yes | CD | Yes | Yes | Yes | Yes | No | Yes | GOOD |
| Machado 2020 [(99)](https://paperpile.com/c/sdFrri/3som) | Yes | Yes | CD | Yes | Yes | Yes | Yes | Yes | Yes | GOOD |
| Mantese 2009 [(39)](https://paperpile.com/c/sdFrri/9azR) | Yes | Yes | CD | Yes | Yes | Yes | Yes | CD | Yes | GOOD |
| Mayoral 2008 [(15)](https://paperpile.com/c/sdFrri/UofQ) | Yes | Yes | CD | Yes | Yes | Yes | NA | Yes | Yes | GOOD |
| Neves Reis 2002 [(43)](https://paperpile.com/c/sdFrri/vjNv) | No | No | Yes | CD | Yes | Yes | Yes | No | Yes | POOR |
| Pérez 2014 [(17)](https://paperpile.com/c/sdFrri/TMwc) | Yes | Yes | CD | Yes | Yes | Yes | Yes | No | Yes | GOOD |
| Pírez 2001 [(100)](https://paperpile.com/c/sdFrri/VEoM) | Yes | Yes | CD | Yes | Yes | Yes | Yes | Yes | Yes | GOOD |
| Pírez 2017 [(102)](https://paperpile.com/c/sdFrri/pNN0) | Yes | Yes | Yes | Yes | Yes | NA | Yes | Yes | Yes | FAIR |
| Pírez García 2008 [(103)](https://paperpile.com/c/sdFrri/dVUs) | Yes | Yes | CD | Yes | Yes | Yes | Yes | Yes | Yes | GOOD |
| Rioseco 2004 [(54)](https://paperpile.com/c/sdFrri/WHcR) | Yes | Yes | CD | Yes | Yes | Yes | Yes | Yes | Yes | GOOD |
| Rioseco 2018 [(55)](https://paperpile.com/c/sdFrri/akuk) | Yes | Yes | CD | Yes | Yes | Yes | Yes | Yes | Yes | GOOD |
| Saldías 2011 [(56)](https://paperpile.com/c/sdFrri/rzbx) | Yes | Yes | CD | Yes | Yes | Yes | Yes | Yes | Yes | GOOD |
| Toraño-Peraza 2014 [(69)](https://paperpile.com/c/sdFrri/89UW) | Yes | Yes | CD | Yes | Yes | Yes | Yes | No | Yes | FAIR |
| Trotman 2009 [(71)](https://paperpile.com/c/sdFrri/vogd) | Yes | Yes | Yes | CD | Yes | Yes | Yes | Yes | Yes | GOOD |
| Ulloa-Gutierrez 2003 [(66)](https://paperpile.com/c/sdFrri/1qjo) | Yes | Yes | Yes | Yes | Yes | Yes | Yes | No | Yes | GOOD |
| Vargas-Gutierrez 2015 [(67)](https://paperpile.com/c/sdFrri/IG1I) | No | Yes | CD | Yes | Yes | Yes | Yes | No | Yes | FAIR |
| Vieira 2007 [(48)](https://paperpile.com/c/sdFrri/LHye) | Yes | Yes | CD | Yes | Yes | Yes | NA | No | Yes | FAIR |

* **NA**: Not applicable, **CD**: Cannot be determined

1. Was the study question or objective clearly specified?
2. Was the study population clearly and fully described, including case definition?
3. Were cases consecutive?
4. Were subjects comparable?
5. Was exposure clearly described?
6. Were measures of results clearly defined, valid, reliable and consistently implemented for all study participants?
7. Was the length of follow-up appropriate?
8. Were statistical methods properly described?
9. Were results properly described?

# Supplementary Table 6. Quality Assessment

| **Author, year** | **Location^1^** | **Collection period^2^** | **Population^3^** | **Identification method^4^** | **≥100 isolates^5^** | **Control strains^6^** | **MIC breackpoints^7^** | **Score^8^** |
| --- | --- | --- | --- | --- | --- | --- | --- | --- |
| Abarca 2008 [(50)](https://paperpile.com/c/sdFrri/MGha) | Chile | 2001-2002 | Yes | Yes | 36 | No | Yes | 5 |
| Abate 2014 [(1)](https://paperpile.com/c/sdFrri/eLUh) | Argentina | 1993-2011 | Yes | No | 537 | Yes | Yes | 6 |
| Agudelo 2006 [(57)](https://paperpile.com/c/sdFrri/pgXM) | Colombia | 1994-2004 | Yes | Yes | 2022 | Yes | Yes | 7 |
| Aguilera 2010 [(51)](https://paperpile.com/c/sdFrri/6k4n) | Chile | 2005-2006 | Yes | Yes | 56 | Yes | Yes | 6 |
| Altclas 2004 [(2)](https://paperpile.com/c/sdFrri/8vUP) | Argentina | 1993-1998 | Yes | Yes | 107 | No | Yes | 6 |
| Alvares 2011 [(20)](https://paperpile.com/c/sdFrri/U8Hu) | Brazil | 1999-2009 | Yes | Yes | 72 | Yes | Yes | 6 |
| Alves Cardozo 2008 [(72)](https://paperpile.com/c/sdFrri/D9UG) | LAC | 1998-2002 | Yes | Yes | 240 | Yes | Yes | 7 |
| Aranda 2014 [(81)](https://paperpile.com/c/sdFrri/3kbU) | Paraguay | 1993-2006 | Yes | Yes | 49 | No | Yes | 5 |
| Arredondo-García 2011 [(76)](https://paperpile.com/c/sdFrri/frXo) | Mexico | 2002-2005 | Yes | Yes | 150 | No | Yes | 6 |
| Assandri 2015 [(92)](https://paperpile.com/c/sdFrri/o0Uc) | Uruguay | 2001-2013 | Yes | No | 25 | No | Yes | 4 |
| Azevedo 2016 [(21)](https://paperpile.com/c/sdFrri/ElfU) | Brazil | 2008-2012 | Yes | Yes | 77 | Yes | Yes | 6 |
| Bakir 2003 [(3)](https://paperpile.com/c/sdFrri/Gt8N) | Argentina | 1993-1999 | Yes | Yes | 274 | No | Yes | 6 |
| Barboza 2018 [(65)](https://paperpile.com/c/sdFrri/AGZP) | Costa Rica | 2009-2015 | Yes | No | 21 | No | No | 3 |
| Barroso 2012 [(22)](https://paperpile.com/c/sdFrri/cMq2) | Brazil | 2000-2008 | Yes | Yes | 250 | Yes | Yes | 7 |
| Bedran 2005 [(23)](https://paperpile.com/c/sdFrri/HesK) | Brazil | 1997-2004 | Yes | Yes | 502 | No | Yes | 6 |
| Benitez 2017 [(4)](https://paperpile.com/c/sdFrri/G7jP) | Argentina | 2013-2014 | Yes | Yes | 17 | No | Yes | 5 |
| Berberian 2014 [(5)](https://paperpile.com/c/sdFrri/7hFe) | Argentina | 1999-2010 | Yes | Yes | 111 | No | Yes | 6 |
| Berezin 2002 [(24)](https://paperpile.com/c/sdFrri/n8eS) | Brazil | 1994-1999 | Yes | Yes | 55 | No | Yes | 5 |
| Berezin 2007 [(25)](https://paperpile.com/c/sdFrri/eojm) | Brazil | 1997-2001 | Yes | Yes | 105 | No | Yes | 6 |
| Berezin 2020 [(26)](https://paperpile.com/c/sdFrri/hkT3) | Brazil | 2005-2015 | Yes | Yes | 260 | No | No | 5 |
| Brandileone 2006 [(27)](https://paperpile.com/c/sdFrri/Gjpx) | Brazil | 1993-2004 | Yes | Yes | 6470 | Yes | Yes | 7 |
| Brandileone 2021 [(28)](https://paperpile.com/c/sdFrri/YLC4) | Brazil | 2007-2019 | Yes | Yes | 11380 | Yes | Yes | 7 |
| Caierao 2014 [(29)](https://paperpile.com/c/sdFrri/Xaj2) | Brazil | 2007-2012 | Yes | Yes | 322 | Yes | Yes | 7 |
| Camacho Moreno 2020 [(58)](https://paperpile.com/c/sdFrri/aLvN) | Colombia | 2008-2017 | Yes | Yes | 56 | No | Yes | 5 |
| Camou 2003 [(93)](https://paperpile.com/c/sdFrri/EmIJ) | Uruguay | 1994-2001 | Yes | Yes | 506 | Yes | Yes | 7 |
| Cassiolato 2019 [(30)](https://paperpile.com/c/sdFrri/nNa6) | Brazil | 2005-2017 | Yes | Yes | 227 | Yes | Yes | 7 |
| Castro 2017 [(85)](https://paperpile.com/c/sdFrri/OFeg) | Peru | 2009-2011 | Yes | Yes | 42 | No | Yes | 5 |
| Castillo-Tokumori 2018 [(86)](https://paperpile.com/c/sdFrri/NgkE) | Peru | 2016-2018 | Yes | No | 22 | No | Yes | 4 |
| Cazentini Medeiros 2017 [(31)](https://paperpile.com/c/sdFrri/9OrS) | Brazil | 1998-2013 | Yes | Yes | 796 | Yes | Yes | 7 |
| Christophe 2018 [(32)](https://paperpile.com/c/sdFrri/2zYg) | Brazil | 2013-2015 | Yes | Yes | 98 | Yes | Yes | 6 |
| Contreras 2002 [(52)](https://paperpile.com/c/sdFrri/Ovfi) | Chile | 1994-1999 | Yes | No | 78 | Yes | Yes | 5 |
| da Silva 2010 [(33)](https://paperpile.com/c/sdFrri/rr9C) | Brazil | 2005-2008 | Yes | Yes | 41 | No | Yes | 5 |
| De León 2011 [(80)](https://paperpile.com/c/sdFrri/chOl) | Panama | 2010-2011 | Yes | Yes | 23 | No | No | 4 |
| dos Santos 2013 [(34)](https://paperpile.com/c/sdFrri/upvW) | Brazil | 2006-2012 | Yes | Yes | 259 | No | Yes | 6 |
| Echaniz-Aviles 2019 [(77)](https://paperpile.com/c/sdFrri/GrrN) | Mexico | 2000-2015 | Yes | Yes | 6 | Yes | Yes | 6 |
| Elenga 2015 [(70)](https://paperpile.com/c/sdFrri/eFrI) | French Guiana | 2000-2010 | Yes | Yes | 28 | No | No | 4 |
| Farfán-Albarracín 2022 [(59)](https://paperpile.com/c/sdFrri/I8ys) | Colombia | 2008-2019 | Yes | Yes | 81 | No | Yes | 5 |
| Ferrari Castilla 2007 [(94)](https://paperpile.com/c/sdFrri/c1OH) | Uruguay | 1998-2004 | Yes | Yes | 412 | No | Yes | 6 |
| Fica 2014 [(53)](https://paperpile.com/c/sdFrri/9TIL) | Chile | 2005-2010 | Yes | Yes | 60 | No | Yes | 5 |
| Fonaroff 2014 [(6)](https://paperpile.com/c/sdFrri/NKtC) | Argentina | 2004-2010 | Yes | Yes | 93 | No | Yes | 5 |
| Fonseca Hernández 2017 [(68)](https://paperpile.com/c/sdFrri/Isga) | Cuba | 2014-2016 | Yes | Yes | 37 | No | No | 4 |
| Gagetti 2017 [(7)](https://paperpile.com/c/sdFrri/JY3N) | Argentina | 1993-2014 | Yes | No | 176 | Yes | Yes | 6 |
| Gagetti 2021 [(8)](https://paperpile.com/c/sdFrri/fKg5) | Argentina | 1998-2013 | Yes | No | 1713 | Yes | Yes | 6 |
| Gentile 2003 [(9)](https://paperpile.com/c/sdFrri/tzWi) | Argentina | 1995-2000 | Yes | Yes | 101 | No | Yes | 7 |
| Gentile 2018a [(10)](https://paperpile.com/c/sdFrri/lekQ) | Argentina | 2007-2014 | Yes | No | 297 | Yes | Yes | 6 |
| Gentile 2018b [(11)](https://paperpile.com/c/sdFrri/94Yh) | Argentina | 2012-2017 | Yes | No | 61 | No | No | 3 |
| Gomes de Oliveira Magalhães 2003 [(35)](https://paperpile.com/c/sdFrri/h45Z) | Brazil | 2000-2001 | Yes | Yes | 31 | Yes | Yes | 6 |
| Gómez-Barreto 2010 [(78)](https://paperpile.com/c/sdFrri/EeOB) | Mexico | 1997-2014 | Yes | Yes | 156 | Yes | Yes | 7 |
| Gouveia 2011 [(36)](https://paperpile.com/c/sdFrri/Y73D) | Brazil | 1995-2005 | Yes | Yes | 548 | No | Yes | 6 |
| Grenon 2005 [(12)](https://paperpile.com/c/sdFrri/ZVxL) | Argentina | 1998-2001 | Yes | Yes | 101 | Yes | Yes | 7 |
| Grenón 2014 [(13)](https://paperpile.com/c/sdFrri/kOSm) | Argentina | 1994-2009 | Yes | Yes | 150 | No | Yes | 6 |
| Gutiérrez-Tobar 2022 [(60)](https://paperpile.com/c/sdFrri/nfFh) | Colombia | 2008-2019 | Yes | Yes | 370 | No | Yes | 6 |
| Hawkins 2017 [(87)](https://paperpile.com/c/sdFrri/aTYC) | Peru | 2006-2011 | Yes | No | 212 | NA^#^ | NA^#^ | 6 |
| Hortal 2000a [(95)](https://paperpile.com/c/sdFrri/JM4M) | Uruguay | 1987-1997 | Yes | Yes | 228 | No | Yes | 6 |
| Hortal 2000b [(73)](https://paperpile.com/c/sdFrri/PyRD) | LAC | 1993-1999 | Yes | Yes | 1396 | No | Yes | 6 |
| Hortal 2001 [(74)](https://paperpile.com/c/sdFrri/66kj) | LAC | 1993-1999 | Yes | Yes | 4105 | Yes | Yes | 7 |
| Hortal 2007 [(96)](https://paperpile.com/c/sdFrri/0NoP) | Uruguay | 2001-2004 | Yes | Yes | 11 | No | No | 4 |
| Hortal 2008 [(97)](https://paperpile.com/c/sdFrri/XtOj) | Uruguay | 2000-2004 | Yes | Yes | 252 | No | No | 5 |
| Laval 2006 [(37)](https://paperpile.com/c/sdFrri/Ihlr) | Brazil | 2000-2001 | Yes | Yes | 125 | No | Yes | 6 |
| Leal Castro 2022 [(61)](https://paperpile.com/c/sdFrri/lbs2) | Colombia | 2011-2017 | Yes | No | 149 | No | Yes | 5 |
| León 2020 [(82)](https://paperpile.com/c/sdFrri/Jc6k) | Paraguay | 2010-2018 | Yes | Yes | 782 | No | Yes | 6 |
| Levin 2003 [(38)](https://paperpile.com/c/sdFrri/NDoO) | Brazil | 1991-1994 | Yes | Yes | 165 | No | Yes | 6 |
| Lovera 2011 [(83)](https://paperpile.com/c/sdFrri/hzFG) | Paraguay | 2000-2010 | Yes | Yes | 26 | No | Yes | 5 |
| Luna-Muschi 2019 [(88)](https://paperpile.com/c/sdFrri/pHCt) | Peru | 2006-2011 | Yes | Yes | 157 | No | Yes | 6 |
| Machado 2014 [(98)](https://paperpile.com/c/sdFrri/2DrY) | Uruguay | 2010 | Yes | Yes | 43 | No | No | 4 |
| Machado 2020 [(99)](https://paperpile.com/c/sdFrri/3som) | Uruguay | 2009-2018 | Yes | Yes | 46 | No | Yes | 5 |
| Mantese 2009 [(39)](https://paperpile.com/c/sdFrri/9azR) | Brazil | 1999-2008 | Yes | Yes | 142 | Yes | Yes | 7 |
| Mathurin 2008 [(14)](https://paperpile.com/c/sdFrri/Gx5y) | Argentina | 2004-2007 | Yes | Yes | 65 | No | Yes | 5 |
| Mayoral 2008 [(15)](https://paperpile.com/c/sdFrri/UofQ) | Argentina | 2003-2005 | Yes | Yes | 76 | Yes | Yes | 6 |
| Menezes 2011 [(40)](https://paperpile.com/c/sdFrri/iXfi) | Brazil | 2000-2007 | Yes | Yes | 397 | Yes | Yes | 7 |
| Morales de Santa Gadea 2003 [(89)](https://paperpile.com/c/sdFrri/zvqc) | Peru | 2000-2001 | Yes | Yes | 13 | No | Yes | 5 |
| Moreno 2020 [(75)](https://paperpile.com/c/sdFrri/j7Oi) | LAC | 2000-2015 | Yes | No | 185 | Yes | Yes | 6 |
| Mott 2014 [(41)](https://paperpile.com/c/sdFrri/KoBe) | Brazil | 2010-2012 | Yes | Yes | 159 | Yes | Yes | 7 |
| Nascimento-Carvalho 2003 [(42)](https://paperpile.com/c/sdFrri/CG71) | Brazil | 1997-2002 | Yes | Yes | 70 | Yes | Yes | 6 |
| Neves Reis 2002 [(43)](https://paperpile.com/c/sdFrri/vjNv) | Brazil | 1995-1999 | Yes | Yes | 303 | No | Yes | 6 |
| Nurse-Lucas 2016 [(91)](https://paperpile.com/c/sdFrri/Iijy) | Trinidad and Tobago | 1997-2013 | Yes | No | NR | No | No | 3 |
| Paganini 2001 [(16)](https://paperpile.com/c/sdFrri/kjnw) | Argentina | 1996-1998 | Yes | Yes | 32 | No | Yes | 5 |
| Parra 2014 [(62)](https://paperpile.com/c/sdFrri/XHNB) | Colombia | 2005-2010 | Yes | Yes | 1775 | No | Yes | 6 |
| Parra 2017 [(63)](https://paperpile.com/c/sdFrri/e0qM) | Colombia | 1994-2013 | Yes | Yes | 76 | No | Yes | 5 |
| Pérez 2014 [(17)](https://paperpile.com/c/sdFrri/TMwc) | Argentina | 2008-2013 | Yes | Yes | 167 | No | Yes | 6 |
| Pinto 2019 [(44)](https://paperpile.com/c/sdFrri/FVou) | Brazil | 1990-2014 | Yes | Yes | 428 | No | Yes | 6 |
| Pírez 2001 [(100)](https://paperpile.com/c/sdFrri/VEoM) | Uruguay | 1997-1998 | Yes | Yes | 41 | No | Yes | 5 |
| Pírez 2014 [(101)](https://paperpile.com/c/sdFrri/YFik) | Uruguay | 2003-2012 | Yes | Yes | 630 | No | No | 5 |
| Pírez 2017 [(102)](https://paperpile.com/c/sdFrri/pNN0) | Uruguay | 2005-2014 | Yes | No | 52 | No | No | 3 |
| Pírez García 2008 [(103)](https://paperpile.com/c/sdFrri/dVUs) | Uruguay | 1998-2005 | Yes | No | 165 | No | Yes | 5 |
| Rioseco 2004 [(54)](https://paperpile.com/c/sdFrri/WHcR) | Chile | 1997-2002 | Yes | Yes | 45 | No | Yes | 5 |
| Rioseco 2018 [(55)](https://paperpile.com/c/sdFrri/akuk) | Chile | 2010-2014 | Yes | Yes | 70 | No | Yes | 5 |
| Rivera-Matos 2005 [(90)](https://paperpile.com/c/sdFrri/raOq) | Puerto Rico | 2001 | Yes | Yes | 177 | Yes | Yes | 7 |
| Rocha Dullius 2018 [(45)](https://paperpile.com/c/sdFrri/RYx2) | Brazil | 2005-2016 | Yes | Yes | 118 | No | Yes | 6 |
| Rossoni 2008 [(46)](https://paperpile.com/c/sdFrri/7KDd) | Brazil | 2001-2002 | Yes | Yes | 100 | No | Yes | 6 |
| Ruvinsky 2010 [(18)](https://paperpile.com/c/sdFrri/9ctC) | Argentina | 1994-2007 | Yes | No | 2205 | Yes | Yes | 6 |
| Saldías 2011 [(56)](https://paperpile.com/c/sdFrri/rzbx) | Chile | 2002-2005 | Yes | Yes | 77 | Yes | Yes | 6 |
| Sanabria 2009 [(84)](https://paperpile.com/c/sdFrri/15Ol) | Paraguay | 2002-2007 | Yes | Yes | 73 | No | Yes | 5 |
| Soares dos Santos 2022 [(47)](https://paperpile.com/c/sdFrri/aRUw) | Brazil | 1996-2012 | Yes | Yes | 854 | Yes | Yes | 7 |
| Toraño-Peraza 2014 [(69)](https://paperpile.com/c/sdFrri/89UW) | Cuba | 2007-2012 | Yes | Yes | 105 | No | Yes | 6 |
| Trotman 2009 [(71)](https://paperpile.com/c/sdFrri/vogd) | Jamaica | 1995-1999 | Yes | Yes | 44 | No | No | 4 |
| Ulloa-Gutierrez 2003 [(66)](https://paperpile.com/c/sdFrri/1qjo) | Costa Rica | 1995-2001 | Yes | Yes | 84 | No | Yes | 5 |
| Vargas-Gutierrez 2015 [(67)](https://paperpile.com/c/sdFrri/IG1I) | Costa Rica | 2006-2014 | Yes | Yes | 35 | No | No | 4 |
| Vela 2001 [(64)](https://paperpile.com/c/sdFrri/xhhk) | Colombia | 1994-1999 | Yes | No | 660 | No | Yes | 5 |
| Vieira 2007 [(48)](https://paperpile.com/c/sdFrri/LHye) | Brazil | 1995-2004 | Yes | Yes | 232 | Yes | Yes | 7 |
| Villaseñor-Sierra 2008 [(79)](https://paperpile.com/c/sdFrri/lBQm) | Mexico | 2000-2005 | Yes | Yes | 31 | Yes | Yes | 6 |
| Yoshioka 2011 [(49)](https://paperpile.com/c/sdFrri/C2P5) | Brazil | 2003-2008 | Yes | Yes | 107 | No | Yes | 6 |
| Zintgraff 2022 [(19)](https://paperpile.com/c/sdFrri/eMKK) | Argentina | 2006-2019 | Yes | Yes | 2908 | Yes | Yes | 7 |

#NA: not applicable. This study used molecular techniques for the detection of genes associated with resistance.

1. Whether the study specified the location where *S. pneumoniae* isolates were collected.
2. Whether the study specified the collection period of the isolates.
3. Whether the study described the population from which *S. pneumoniae* isolates were obtained.
4. Whether the study described the method of identifying *S. pneumoniae* isolates.
5. Whether the study included at least 100 tested *S. pneumoniae* isolates.
6. Whether the study utilized control strains recommended by WHO in determining MICs or implied it.
7. Whether the study described the method for determining the antimicrobial susceptibility of isolates or the MICs values for susceptibility, reduced susceptibility and resistance isolates.
8. SCORE: Each of the seven criteria were rated one point if a study satisfied it. Studies that scored five or higher were considered “high quality”, three or four as “moderate quality”, and two or less as “low quality”.

# Supplementary Table 7. S. pneumoniae breakpoints (CLSI M100-Ed 33) used for analysis

| **Antimicrobial Agents** | **Disk content** | **Interpretive Categories and Zone Diameter Breakpoints (mm)** | | | **Interpretive Categories and MIC Breakpoints (µg/mL)** | | |  |
| --- | --- | --- | --- | --- | --- | --- | --- | --- |
|  |  | **S*** | **I*** | **R*** | **S*** | **I*** | **R*** | **Comments** |
| Oxacillin | 1 µg | ≥ 20 | - | - | - | - | - | Isolates with oxacillin zone sizes ≥ 20 mm are susceptible (MIC ≤0.06 µg/ml) to penicillin |
| Penicillin (meningitis) | - | - | - | - | ≤ 0.06 | - | ≥ 0.12 |  |
| Ceftriaxone/cefotaxime  (meningitis) | - | - | - | - | ≤ 0.5 | 1 | ≥ 2 |  |
| Amoxicillin | - | - | - | - | ≤ 2 | 4 | ≥ 8 |  |
| Cefuroxime | - | - | - | - | ≤ 0.5 | 1 | ≥ 2 |  |
| Meropenem | - | - | - | - | ≤ 0.25 | 0.5 | ≥ 1 |  |
| Erythromycin | 15 µg | ≥ 21 | 16-20 | ≤ 15 | ≤ 0.25 | 0.5 | ≥ 1 |  |
| Azithromycin | 15 µg | ≥ 18 | 14-17 | ≤ 13 | ≤ 0.5 | 1 | ≥ 2 |  |
| Clindamycin | 2 µg | ≥ 19 | 16-18 | ≤ 15 | ≤ 0.25 | 0.5 | ≥ 1 |  |
| Tetracycline | 30 µg | ≥ 28 | 25-27 | ≤ 24 | ≤ 1 | 2 | ≥ 4 |  |
| Doxycycline | 30 µg | ≥ 28 | 25-27 | ≤ 24 | ≤ 0.25 | 0.5 | ≥ 1 |  |
| Trimetoprim-sulfametoxazol | 1.25/23.75 µg | ≥ 19 | 16-18 | ≤ 15 | ≤ 0.5/9.5 | 1/19-2/38 | ≥ 4/76 |  |
| Levofloxacin | 5 µg | ≥ 17 | 14-16 | ≤ 13 | ≤ 2 | 4 | ≥ 8 |  |
| Ofloxacin | 5 µg | ≥ 16 | 13-15 | ≤ 12 | ≤ 2 | 4 | ≥ 8 |  |
| Vancomycin | 30 µg | ≥ 17 | - | - | ≤ 1 | - | - |  |
| Rifampicin | 5 µg | ≥ 19 | 17-18 | ≤ 16 | ≤ 1 | 2 | ≥ 4 |  |
| Chloramphenicol | 30 µg | ≥ 21 | - | ≤ 20 | ≤ 4 | - | ≥ 8 |  |
| Linezolid | 30 µg | ≥ 21 | - | - | ≤ 2 | - | - |  |

*S: susceptible, I: Intermediate, R: Resistant

# Resistance to penicillin, ceftriaxone/cefotaxime, trimethoprim-sulfamethoxazole and erythromycin by type of IPD

## Supplementary Table 8. Resistance to penicillin, ceftriaxone/cefotaxime, trimethoprim-sulfamethoxazole and erythromycin from IPD cases

|  | | | **PEN*** | | **CRO/CTX*** | | **TMS*** | | **ERY*** | |
| --- | --- | --- | --- | --- | --- | --- | --- | --- | --- | --- |
|  |  |  | **Number**  **of isolates evaluated (N)** | **R** % (N)** | **Number**  **of isolates evaluated (N)** | **R** % (N)** | **Number**  **of isolates evaluated (N)** | **R** % (N)** | **Number**  **of isolates evaluated (N)** | **R** % (N)** |
| **Author, year** | **Country** | **Period** |  |  |  |  |  |  |  |  |
| Benitez 2017 [(4)](https://paperpile.com/c/sdFrri/G7jP) | Argentina | 1993-1994 | 16 | 37.5% (6) | 17 | 0% (0) | 14 | 42.9% (6) | 15 | 13.3% (2) |
| Bakir 2003 [(3)](https://paperpile.com/c/sdFrri/Gt8N) | Argentina | 1993-1999 | 274 | 31.8% (87) | 274 | 20.8% (57) | - | - | - | - |
| Hortal 2001 [(74)](https://paperpile.com/c/sdFrri/66kj) | Argentina | 1993-1999 | 1006 | 30.6% (308) | 865 | 19.9% (172) | 1006 | 54.2% (545) | 1006 | 2.1% (21) |
| Abate 2014 [(1)](https://paperpile.com/c/sdFrri/eLUh) | Argentina | 1993-2011 | 537 | 3.4% (18) | 537 | 0.4% (2) | - | - | - | - |
| Gagetti 2017# [(7)](https://paperpile.com/c/sdFrri/JY3N) | Argentina | 1993-2014 | 176 | 65.9% (116) | 176 | 2.3% (4) | 176 | 16.5% (29) | 176 | 15.9% (28) |
| Ruvinsky 2010 [(18)](https://paperpile.com/c/sdFrri/9ctC) | Argentina | 1994-2007 | 2196 | 33.2% (728) | 2205 | 4.7% (104) | 2196 | 53.1% (1166) | 2196 | 8.7% (191) |
| Grenon 2005 [(12)](https://paperpile.com/c/sdFrri/ZVxL) | Argentina | 1998-2001 | 101 | 39.6% (40) | 71 | 4.2% (3) | 101 | 47.5% (48) | 101 | 6.9% (7) |
| Gagetti 2021 [(8)](https://paperpile.com/c/sdFrri/fKg5) | Argentina | 1998-2013 | 1713 | 34.9% (598) | - | - | 1713 | 45% (770) | 1713 | 21.5% (368) |
| Mayoral 2008 [(15)](https://paperpile.com/c/sdFrri/UofQ) | Argentina | 2003-2005 | 76 | 21.1% (16) | - | - | - | - | - | - |
| Zintgraff 2022 [(19)](https://paperpile.com/c/sdFrri/eMKK) | Argentina | 2006-2019 | 2798 | 33.2% (929) | 2798 | 5.5% (153) | 2798 | 43.4% (1213) | 2798 | 26.5% (742) |
| Pinto 2019 [(44)](https://paperpile.com/c/sdFrri/FVou) | Brazil | 1990-2014 | 428 | 13.6% (58) | - | - | - | - | - | - |
| Levin 2003 [(38)](https://paperpile.com/c/sdFrri/NDoO) | Brazil | 1991-1994 | 165 | 22.4% (37) | - | - | - | - | - | - |
| Hortal 2001 [(74)](https://paperpile.com/c/sdFrri/66kj) | Brazil | 1993-1999 | 1206 | 20.6% (249) | 1206 | 0.7% (8) | - | - | - | - |
| Brandileone 2006 [(27)](https://paperpile.com/c/sdFrri/Gjpx) | Brazil | 1993-2004 | 6470 | 21% (1359) | 4326 | 0.1% (5) | 3817 | 65% (2481) | 3817 | 6.2% (237) |
| Berezin 2007 [(25)](https://paperpile.com/c/sdFrri/eojm) | Brazil | 1997-2001 | 105 | 29.5% (31) | - | - | - | - | - | - |
| Nascimento Carvalho  2003 [(42)](https://paperpile.com/c/sdFrri/CG71) | Brazil | 1997-2002 | 70 | 20% (14) | 17 | 5.9% (1) | 70 | 65.7% (46) | 70 | 5.7% (4) |
| Bedran 2005 [(23)](https://paperpile.com/c/sdFrri/HesK) | Brazil | 1997-2004 | 502 | 11.8% (59) | - | - | - | - | - | - |
| Cazentini Medeiros  2017 [(31)](https://paperpile.com/c/sdFrri/9OrS) | Brazil | 1998-2003 | 796 | 14.8% (118) | 787 | 1% (8) | 708 | 49.4% (350) | 701 | 5.1% (36) |
| Mantese 2009 [(39)](https://paperpile.com/c/sdFrri/9azR) | Brazil | 1999-2008 | 142 | 9.9% (14) | - | - | - | - | - | - |
| Laval 2006 [(37)](https://paperpile.com/c/sdFrri/Ihlr) | Brazil | 2000-2001 | 125 | 19.2% (24) | - | - | - | - | - | - |
| Gomes de Oliveira  Magalhães 2003 [(35)](https://paperpile.com/c/sdFrri/h45Z) | Brazil | 2000-2001 | 31 | 41.9% (13) | - | - | 31 | 16.1% (5) | 31 | 0% (0) |
| Cassiolato 2019# [(30)](https://paperpile.com/c/sdFrri/nNa6) | Brazil | 2005-2014 | 227 | 69.2% (157) | 227 | 13.2% (30) | 227 | 69.2% (157) | 227 | 39.6% (90) |
| Berezin 2020 [(26)](https://paperpile.com/c/sdFrri/hkT3) | Brazil | 2005-2015 | 260 | 17.3% (45) | 260 | 7.3% (19) | - | - | - | - |
| Rocha Dullius 2018 [(45)](https://paperpile.com/c/sdFrri/RYx2) | Brazil | 2005-2016 | 118 | 9.3% (11) | - | - | 118 | 37.3% (44) | 118 | 17.8% (21) |
| dos Santos 2013 [(34)](https://paperpile.com/c/sdFrri/upvW) | Brazil | 2006-2012 | 258 | 30.6% (79) | 245 | 0.4% (1) | 259 | 55.6% (144) | - | - |
| Caierao 2014 [(29)](https://paperpile.com/c/sdFrri/Xaj2) | Brazil | 2007-2012 | 322 | 42.9% (138) | 322 | 18.6% (80) | - | - | 288 | 5.6% (16) |
| Brandileone 2021 [(28)](https://paperpile.com/c/sdFrri/YLC4) | Brazil | 2007-2019 | 10924 | 18.6% (2034) | 10924 | 7.7% (838) | 11324 | 13.2% (1496) | 11337 | 17.8% (2022) |
| Mott 2014 [(41)](https://paperpile.com/c/sdFrri/KoBe) | Brazil | 2010-2012 | 159 | 0.6% (1) | 159 | 0% (0) | 159 | 20.1% (32) | 159 | 8.2% (13) |
| Christophe 2018 [(32)](https://paperpile.com/c/sdFrri/2zYg) | Brazil | 2013-2015 | 98 | 31.6% (31) | 98 | 7.1% (7) | 98 | 6.1% (6) | 97 | 16.5% (16) |
| Contreras 2002 [(52)](https://paperpile.com/c/sdFrri/Ovfi) | Chile | 1994-1999 | 78 | 35.9% (28) | 78 | 2.6% (2) | - | - | - | - |
| Hortal 2001 [(74)](https://paperpile.com/c/sdFrri/66kj) | Chile | 1994-1999 | 526 | 28.1% (148) | 414 | 8.9% (37) | 526 | 34.4% (181) | 526 | 8.4% (44) |
| Abarca 2008 [(50)](https://paperpile.com/c/sdFrri/MGha) | Chile | 2001-2002 | 36 | 19.4% (7) | 36 | 0% (0) | - | - | - | - |
| Hortal 2001 [(74)](https://paperpile.com/c/sdFrri/66kj) | Colombia | 1994-1999 | 623 | 23.1% (144) | 542 | 11.3% (61) | 623 | 35% (218) | 623 | 4.7% (29) |
| Vela 2001 [(64)](https://paperpile.com/c/sdFrri/xhhk) | Colombia | 1994-1999 | 660 | 25.3% (167) | 167 | 17.4% (29) | 167 | 64.7% (108) | - | - |
| Parra 2017 [(63)](https://paperpile.com/c/sdFrri/e0qM) | Colombia | 1994-2003 | 76 | 2.6% (2) | 61 | 0% (0) | 76 | 13.2% (10) | 61 | 0% (0) |
| Agudelo 2006 [(57)](https://paperpile.com/c/sdFrri/pgXM) | Colombia | 1994-2004 | 2022 | 29.8% (602) | 2022 | 2.1% (43) | 2022 | 29.8% (602) | 2022 | 2.9% (59) |
| Parra 2014 [(62)](https://paperpile.com/c/sdFrri/XHNB) | Colombia | 2005-2010 | 1775 | 14.6% (260) | 1775 | 1.2% (21) | 1775 | 34% (604) | 1775 | 6.6% (118) |
| Camacho Moreno 2020# [(58)](https://paperpile.com/c/sdFrri/aLvN) | Colombia | 2008-2017 | 56 | 39.3% (22) | - | - | - | - | - | - |
| Leal Castro 2022 [(61)](https://paperpile.com/c/sdFrri/lbs2) | Colombia | 2011-2017 | 149 | 12.8% (19) | 145 | 0.7% (1) | - | - | - | - |
| Ulloa-Gutierrez 2003 [(66)](https://paperpile.com/c/sdFrri/1qjo) | Costa Rica | 1995-2001 | 84 | 14.3% (12) | 66 | 3% (2) | - | - | - | - |
| Fonseca Hernández  2017 [(68)](https://paperpile.com/c/sdFrri/Isga) | Cuba | 2014-2016 | - | - | - | - | 34 | 55.9% (19) | 6 | 50% (3) |
| Moreno 2020# [(75)](https://paperpile.com/c/sdFrri/j7Oi) | LAC | 2000-2015 | 185 | 68.6% (127) | - | - | 185 | 66.7% (116) | 185 | 43.2% (80) |
| Hortal 2001 [(74)](https://paperpile.com/c/sdFrri/66kj) | Mexico | 1994-1999 | 418 | 51.7% (216) | 418 | 22% (92) | 418 | 53.3% (223) | 418 | 22.2% (93) |
| Gómez-Barreto 2010 [(78)](https://paperpile.com/c/sdFrri/EeOB) | Mexico | 1997-2014 | 156 | 22.4% (35) | 156 | 25.6% (40) | - | - | - | - |
| Arredondo García 2011 [(76)](https://paperpile.com/c/sdFrri/frXo) | Mexico | 2002-2005 | 150 | 44.7% (67) | 150 | 6% (9) | 150 | 38.7% (58) | 150 | 28.7% (43) |
| De León 2011 [(80)](https://paperpile.com/c/sdFrri/chOl) | Panama | 2010-2011 | 23 | 0% (0) | - | - | 23 | 21.7% (5) | - | - |
| Sanabria 2009 [(84)](https://paperpile.com/c/sdFrri/15Ol) | Paraguay | 2002-2007 | 73 | 20.5% (15) | 73 | 1.4% (1) | 73 | 58.9% (43) | 73 | 9.6% (7) |
| León 2020 [(82)](https://paperpile.com/c/sdFrri/Jc6k) | Paraguay | 2010-2018 | 782 | 7.2% (56) | 782 | 0.4% (3) | - | - | - | - |
| Morales de Santa Gadea 2003 [(89)](https://paperpile.com/c/sdFrri/zvqc) | Peru | 2000-2001 | 13 | 23.1% (3) | 13 | 0% (0) | 13 | 23.1% (3) | 13 | 7.7% (1) |
| Hawkins 2017# [(87)](https://paperpile.com/c/sdFrri/aTYC) | Peru | 2006-2011 | 212 | 49.5% (105) | 46 | 50% (23) | 212 | 71.4% (157) | 212 | 35.8% (76) |
| Luna-Muschi 2019 [(88)](https://paperpile.com/c/sdFrri/pHCt) | Peru | 2006-2011 | 157 | 19.1% (30) | 157 | 3.2% (5) | 101 | 63.4% (64) | 157 | 29.3% (46) |
| Castro 2017 [(85)](https://paperpile.com/c/sdFrri/OFeg) | Peru | 2009-2011 | 42 | 11.9% (5) | 34 | 0% (0) | 42 | 69% (29) | 42 | 35.7% (15) |
| Castillo-Tokumori 2018# [(86)](https://paperpile.com/c/sdFrri/NgkE) | Peru | 2016-2018 | 22 | 22.7% (5) | - | - | - | - | - | - |
| Rivera-Matos 2005 [(90)](https://paperpile.com/c/sdFrri/raOq) | Puerto Rico | 2001 | 177 | 49.7% (88) | 177 | 3.4% (6) | 177 | 31.6% (56) | 177 | 25.4% (45) |
| Nurse-Lucas 2016# [(91)](https://paperpile.com/c/sdFrri/Iijy) | Trinidad and Tobago | 1997-2013 | NR | 12 | - | - | NR | 4 | NR | 14 |
| Hortal 2001 [(74)](https://paperpile.com/c/sdFrri/66kj) | Uruguay | 1993-1999 | 326 | 35.9% (117) | 326 | 26.1% (85) | 326 | 47.2% (154) | 326 | 3.7% (12) |
| Hortal 2000a [(95)](https://paperpile.com/c/sdFrri/JM4M) | Uruguay | 1987-1997 | - | - | - | - | 228 | 12.3% (28) | - | - |
| Camou 2003 [(93)](https://paperpile.com/c/sdFrri/EmIJ) | Uruguay | 1994-2001 | 506 | 37.4% (189) | - | - | - | - | - | - |
| Assandri 2015 [(92)](https://paperpile.com/c/sdFrri/o0Uc) | Uruguay | 2001-2013 | 25 | 1% (4) | 25 | 0% (0) | - | - | - | - |

*PEN: penicillin; CRO/CTX: ceftriaxone/cefotaxime; TMS: trimethoprim-sulfamethoxazole; ERY: erythromycin

**R: resistant

NR: Not reported

#: This studies were not included in the final analysis of antimicrobial susceptibility

## Supplementary Table 9. Resistance to penicillin, ceftriaxone/cefotaxime, trimethoprim-sulfamethoxazole, and erythromycin from meningitis cases

|  | | | **PEN*** | | **CRO/CTX*** | | **TMS*** | | **ERY*** | |
| --- | --- | --- | --- | --- | --- | --- | --- | --- | --- | --- |
|  |  |  | **Number**  **of isolates evaluated (N)** | **R** % (N)** | **Number of isolates evaluated (N)** | **R** % (N)** | **Number**  **of isolates evaluated (N)** | **R** % (N)** | **Number**  **of isolates evaluated (N)** | **R** % (N)** |
| **Author, year** | **Country** | **Period** |  |  |  |  |  |  |  |  |
| Grenón 2014 [(13)](https://paperpile.com/c/sdFrri/kOSm) | Argentina | 1994-2009 | 150 | 30.7% (46) | 150 | 16.7% (25) | - | - | - | - |
| Berberian 2014 [(5)](https://paperpile.com/c/sdFrri/7hFe) | Argentina | 1999-2010 | 111 | 15.3% (17) | 111 | 5.4% (6) | - | - | - | - |
| Berezin 2002 [(24)](https://paperpile.com/c/sdFrri/n8eS) | Brazil | 1994-1999 | 55 | 36.4% (20) | - | - | - | - | - | - |
| Neves Reis 2002 [(43)](https://paperpile.com/c/sdFrri/vjNv) | Brazil | 1995-1999 | 303 | 15.2% (46) | 303 | 0% (0) | 303 | 9.9% (30) | 303 | 0% (0) |
| Vieira 2007 [(48)](https://paperpile.com/c/sdFrri/LHye) | Brazil | 1995-2004 | 232 | 20.3% (47) | 232 | 0% (0) | - | - | - | - |
| Gouveia 2011 [(36)](https://paperpile.com/c/sdFrri/Y73D) | Brazil | 1995-2005 | 548 | 16.8% (92) | 548 | 0% (0) | - | - | - | - |
| Soares dos Santos 2015 [(47)](https://paperpile.com/c/sdFrri/aRUw) | Brazil | 1996-2012 | 854 | 20.3% (173) | - | - | 854 | 48% (410) | - | - |
| Alvares 2011 [(20)](https://paperpile.com/c/sdFrri/U8Hu) | Brazil | 1999-2009 | 72 | 23.6% (17) | 72 | 12.5% (9) | - | - | - | - |
| Menezes 2011 [(40)](https://paperpile.com/c/sdFrri/iXfi) | Brazil | 2000-2007 | 397 | 22.2% (88) | 397 | 1% (4) | 397 | 56.2% (223) | 397 | 0.8% (3) |
| Barroso 2012 [(22)](https://paperpile.com/c/sdFrri/cMq2) | Brazil | 2000-2008 | 250 | 12.4% (31) | - | - | 250 | 22.4 (56%) | 250 | 4.8% (12) |
| Rossoni 2008 [(46)](https://paperpile.com/c/sdFrri/7KDd) | Brazil | 2001-2002 | 100 | 15% (15) | 100 | 1% (1) | - | - | - | - |
| da Silva 2010 [(33)](https://paperpile.com/c/sdFrri/rr9C) | Brazil | 2005-2008 | 14 | 7.1% (1) | - | - | 12 | 75% (9) | 19 | 15.8% (3) |
| Azevedo 2016 [(21)](https://paperpile.com/c/sdFrri/ElfU) | Brazil | 2008-2012 | 77 | 6.5% (5) | 77 | 0% (0) | 77 | 36.4% (28) | 77 | 1.3% (1) |
| Farfán-Albarracín 2022 [(59)](https://paperpile.com/c/sdFrri/I8ys) | Colombia | 2008-2019 | 81 | 28.4% (23) | 81 | 14.8% (12) | - | - | - | - |
| Barboza 2018 [(65)](https://paperpile.com/c/sdFrri/AGZP) | Costa Rica | 2009-2015 | 21 | 0% (0) | - | - | - | - | - | - |
| Toraño-Peraza 2014 [(69)](https://paperpile.com/c/sdFrri/89UW) | Cuba | 2007-2012 | 105 | 44.8% (47) | 105 | 7.6% (8) | 105 | 24.4% (26) | 105 | 41.9% (44) |
| Elenga 2015 [(70)](https://paperpile.com/c/sdFrri/eFrI) | French Guiana | 2000-2010 | 28 | 35.7% (10) | - | - | - | - | - | - |
| Trotman 2009 [(71)](https://paperpile.com/c/sdFrri/vogd) | Jamaica | 1995-1999 | - | - | 44 | 0% (0) | - | - | - | - |
| Villaseñor-Sierra  2008 [(79)](https://paperpile.com/c/sdFrri/lBQm) | Mexico | 2000-2005 | 31 | 64.5% (20) | 31 | 0% (0) | 31 | 58.1% (18) | 31 | 22.6% (7) |
| Echaniz-Aviles 2019 [(77)](https://paperpile.com/c/sdFrri/GrrN) | Mexico | 2000-2015 | 6 | 50% (3) | 6 | 0% (0) | - | - | - | - |
| Aranda 2014 [(81)](https://paperpile.com/c/sdFrri/3kbU) | Paraguay | 1993-2006 | 49 | 20.4% (10) | 49 | 2% (1) | - | - | - | - |
| Lovera 2011 [(83)](https://paperpile.com/c/sdFrri/hzFG) | Paraguay | 2000-2010 | 26 | 26.9% (7) | 26 | 7.7% (2) | - | - | - | - |
| Pírez 2017 [(102)](https://paperpile.com/c/sdFrri/pNN0) | Uruguay | 2005-2014 | 52 | 13.5% (7) | - | - | - | - | - | - |

*PEN: penicillin; CRO/CTX: ceftriaxone/cefotaxime; TMS: trimethoprim-sulfamethoxazole; ERY: erythromycin

**R: resistant

NR: Not reported

## Supplementary Table 10. Resistance to penicillin, ceftriaxone/cefotaxime, trimethoprim-sulfamethoxazole and erythromycin from pneumonia cases

|  | | | **PEN*** | | **CRO/CTX*** | | **TMS*** | | **ERY*** | |
| --- | --- | --- | --- | --- | --- | --- | --- | --- | --- | --- |
|  |  |  | **Number**  **of isolates evaluated (N)** | **R** % (N)** | **Number**  **of isolates evaluated (N)** | **R** % (N)** | **Number**  **of isolates evaluated (N)** | **R** % (N)** | **Number**  **of isolates evaluated (N)** | **R** % (N)** |
| **Author, year** | **Country** | **Period** |  |  |  |  |  |  |  |  |
| Hortal 2000b [(73)](https://paperpile.com/c/sdFrri/PyRD) | Argentina | 1993-1999 | 430 | 35.6% (153) | 430 | 5.6% (24) | 430 | 61.9% (266) | 430 | 2.6% (11) |
| Gentile 2003 [(9)](https://paperpile.com/c/sdFrri/tzWi) | Argentina | 1995-2000 | 101 | 3% (3) | 101 | 0% (0) | 101 | 67.3% (68) | 101 | 7.9% (8) |
| Paganini 2001 [(16)](https://paperpile.com/c/sdFrri/kjnw) | Argentina | 1996-1998 | 32 | 37.5% (12) | - | - | - | - | - | - |
| Fonaroff 2014 [(6)](https://paperpile.com/c/sdFrri/NKtC) | Argentina | 2004-2010 | 93 | 6.5% (6) | - | - | - | - | - | - |
| Gentile 2018a [(10)](https://paperpile.com/c/sdFrri/lekQ) | Argentina | 2012-2014 | 297 | 4% (12) | - | - | - | - | - | - |
| Gentile 2018b [(11)](https://paperpile.com/c/sdFrri/94Yh) | Argentina | 2012-2017 | 61 | 0% (0) | - | - | - | - | - | - |
| Hortal 2000b [(73)](https://paperpile.com/c/sdFrri/PyRD) | Brazil | 1993-1999 | 345 | 23.2% (80) | 345 | 0% (0) | - | - | - | - |
| Yoshioka 2011# [(49)](https://paperpile.com/c/sdFrri/C2P5) | Brazil | 2003-2008 | 107 | NR | 80 | NR | 105 | NR | 106 | 95.3% (101) |
| Rioseco 2004 [(54)](https://paperpile.com/c/sdFrri/WHcR) | Chile | 1997-2002 | 45 | 6.7% (3) | 45 | 0% (0) | - | - | - | - |
| Saldías 2011 [(56)](https://paperpile.com/c/sdFrri/rzbx) | Chile | 2002-2005 | 77 | 0% (0) | - | - | - | - | - | - |
| Fica 2014 [(53)](https://paperpile.com/c/sdFrri/9TIL) | Chile | 2005-2010 | 60 | 1.7% (1) | 59 | 0% (0) | - | - | - | - |
| Rioseco 2018 [(55)](https://paperpile.com/c/sdFrri/akuk) | Chile | 2010-2014 | 70 | 0% (0) | 70 | 0% (0) | - | - | - | - |
| Hortal 2000b [(73)](https://paperpile.com/c/sdFrri/PyRD) | Colombia | 1994-1999 | 224 | 20.1% (45) | 224 | 1.3% (3) | 224 | 33.9% (76) | 224 | 6.3% (14) |
| Gutierrez-Tobar 2022 [(60)](https://paperpile.com/c/sdFrri/nfFh) | Colombia | 2008-2019 | 370 | 17.8% (66) | 370 | 4.3% (16) | - | - | 370 | 18.4% (68) |
| Vargas-Gutierrez 2015 [(67)](https://paperpile.com/c/sdFrri/IG1I) | Costa Rica | 2006-2014 | 35 | 48.6% (17) | - | - | - | - | - | - |
| Alves Cardozo 2008 [(72)](https://paperpile.com/c/sdFrri/D9UG) | LAC | 1998-2002 | 240 | 50% (240) | - | - | - | - | - | - |
| Hortal 2000b [(73)](https://paperpile.com/c/sdFrri/PyRD) | Mexico | 1994-1999 | 192 | 49.5% (95) | 192 | 20.3% (39) | 192 | 58.9% (113) | 192 | 24.5% (47) |
| Hortal 2000b [(73)](https://paperpile.com/c/sdFrri/PyRD) | Uruguay | 1993-1999 | 205 | 42.9% (88) | 205 | 5.4% (11) | 205 | 53.2% (109) | 205 | 3.9% (8) |
| Pírez 2001 [(100)](https://paperpile.com/c/sdFrri/VEoM) | Uruguay | 1997-1998 | 41 | 26.8% (11) | 32 | 6.3% (2) | - | - | - | - |
| Ferrari Castilla 2007 [(94)](https://paperpile.com/c/sdFrri/c1OH) | Uruguay | 1998-2004 | 412 | 34% (140) | 412 | 1.7% (7) | - | - | - | - |
| Pírez García 2008 [(103)](https://paperpile.com/c/sdFrri/dVUs) | Uruguay | 1998-2005 | 165 | 47.3% (78) | - | - | - | - | - | - |
| Hortal 2008 [(97)](https://paperpile.com/c/sdFrri/XtOj) | Uruguay | 2000-2008 | 252 | 62.7% (158) | - | - | - | - | - | - |
| Hortal 2007# [(96)](https://paperpile.com/c/sdFrri/0NoP) | Uruguay | 2001-2004 | 11 | 81.8% (9) | - | - | 11 | 81.8% (9) | - | - |
| Pírez 2014 [(101)](https://paperpile.com/c/sdFrri/YFik) | Uruguay | 2003-2012 | 630 | 0.2% (1) | - | - | - | - | - | - |
| Machado 2020 [(99)](https://paperpile.com/c/sdFrri/3som) | Uruguay | 2009-2018 | 46 | 0% (0) | 46 | 0% (0) | - | - | - | - |
| Machado 2014 [(98)](https://paperpile.com/c/sdFrri/2DrY) | Uruguay | 2010 | 43 | 0% (0) | - | - | - | - | - | - |

*PEN: penicillin; CRO/CTX: ceftriaxone/cefotaxime; TMS: trimethoprim-sulfamethoxazole; ERY: erythromycin

**R: resistant

NR: Not reported

#: This studies were not included in the final analysis of antimicrobial susceptibility

## Supplementary Table 11. Resistance to penicillin, ceftriaxone/cefotaxime, trimethoprim-sulfamethoxazole and erythromycin from bacteremia cases

|  | | | **PEN*** | | **CRO/CTX*** | | **TMS*** | | **ERY*** | |
| --- | --- | --- | --- | --- | --- | --- | --- | --- | --- | --- |
|  |  |  | **Number**  **of isolates evaluated (N)** | **R** % (N)** | **Number**  **of isolates evaluated (N)** | **R** % (N)** | **Number**  **of isolates evaluated (N)** | **R** % (N)** | **Number**  **of isolates evaluated (N)** | **R** % (N)** |
| **Author, year** | **Country** | **Period** |  |  |  |  |  |  |  |  |
| Altclas 2004 [(2)](https://paperpile.com/c/sdFrri/8vUP) | Argentina | 1993-1998 | 107 | 20.6% (22) | 107 | 0% (0) | - | - | - | - |
| Mathurin 2008 [(14)](https://paperpile.com/c/sdFrri/Gx5y) | Argentina | 2004-2007 | 65 | 6.2% (4) | - | - | - | - | - | - |
| Pérez 2014 [(17)](https://paperpile.com/c/sdFrri/TMwc) | Argentina | 2008-2013 | 167 | 0% (0) | - | - | - | - | - | - |
| Aguilera 2010 [(51)](https://paperpile.com/c/sdFrri/6k4n) | Chile | 2005-2006 | 56 | 1.8% (1) | 56 | 0% (0) | 56 | 17.9% (10) | 56 | 17.9% (10) |

*PEN: penicillin; CRO/CTX: ceftriaxone/cefotaxime; TMS: trimethoprim-sulfamethoxazole; ERY: erythromycin

**R: resistant

NR: Not reported

# Proportion meta-analysis

## Supplementary Figure 1. Proportion meta-analysis of resistance to penicillin


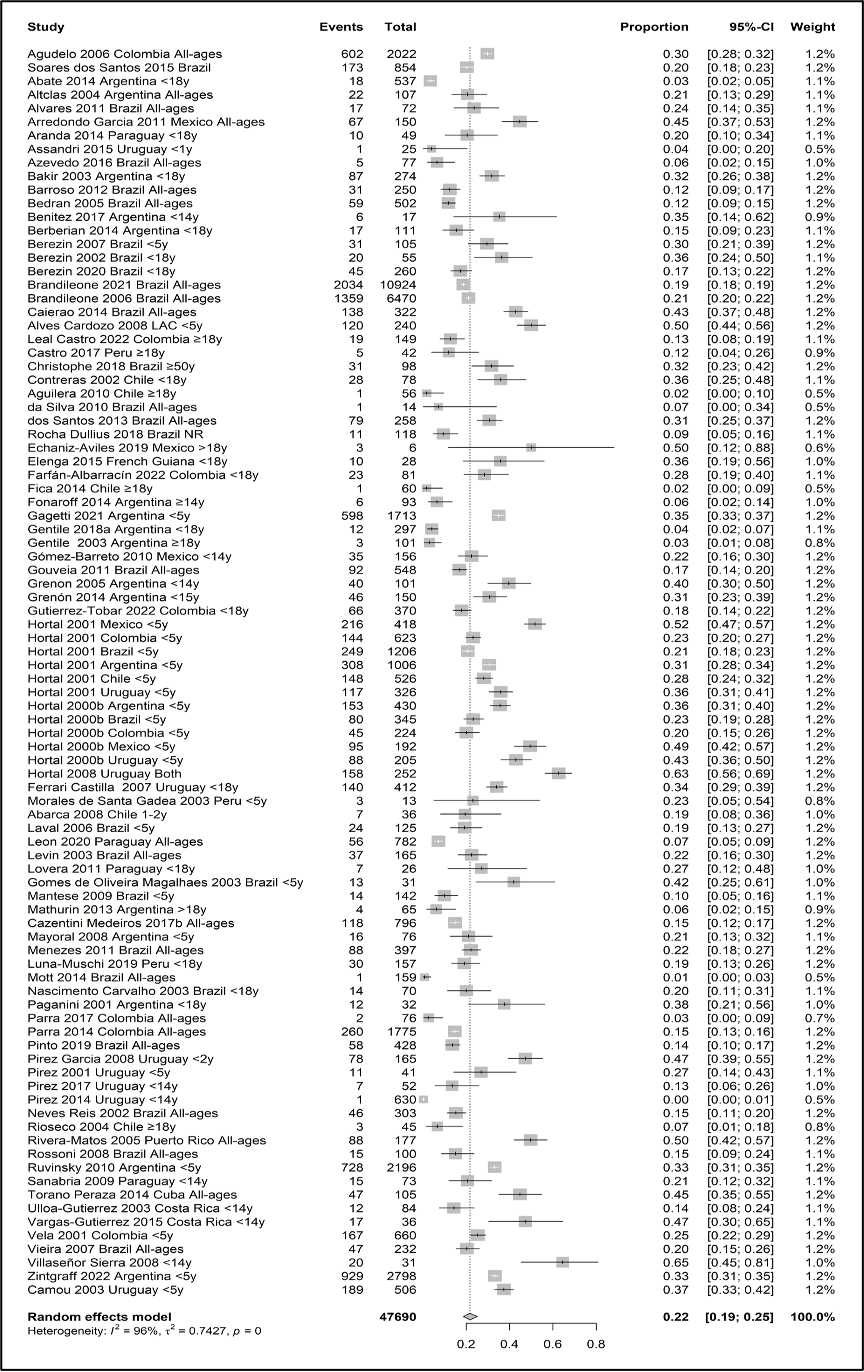


## Supplementary Figure 2. Proportion meta-analysis of resistance to penicillin by five-year calendar period


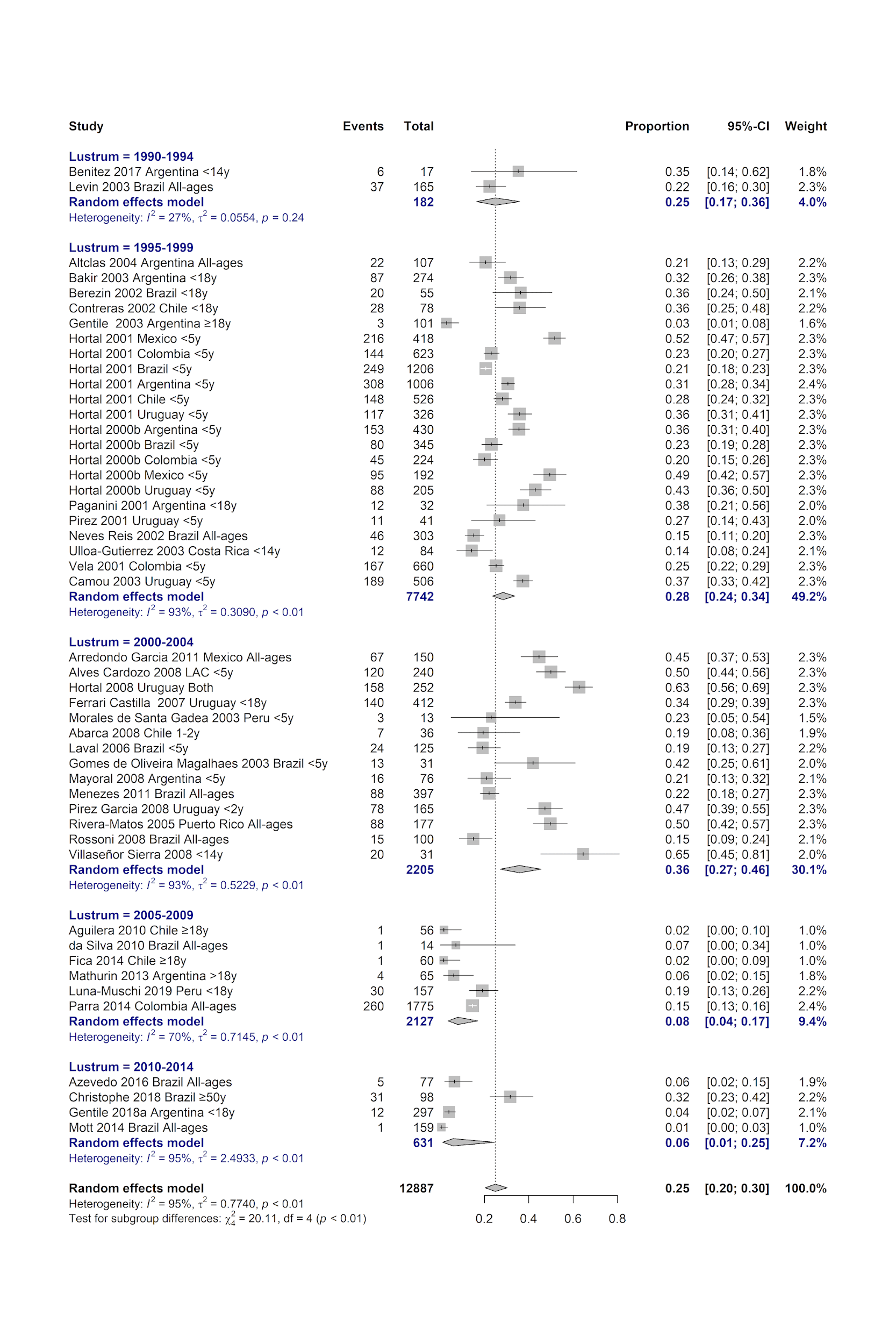


## Supplementary Figure 3. Proportion meta-analysis of resistance to penicillin by age


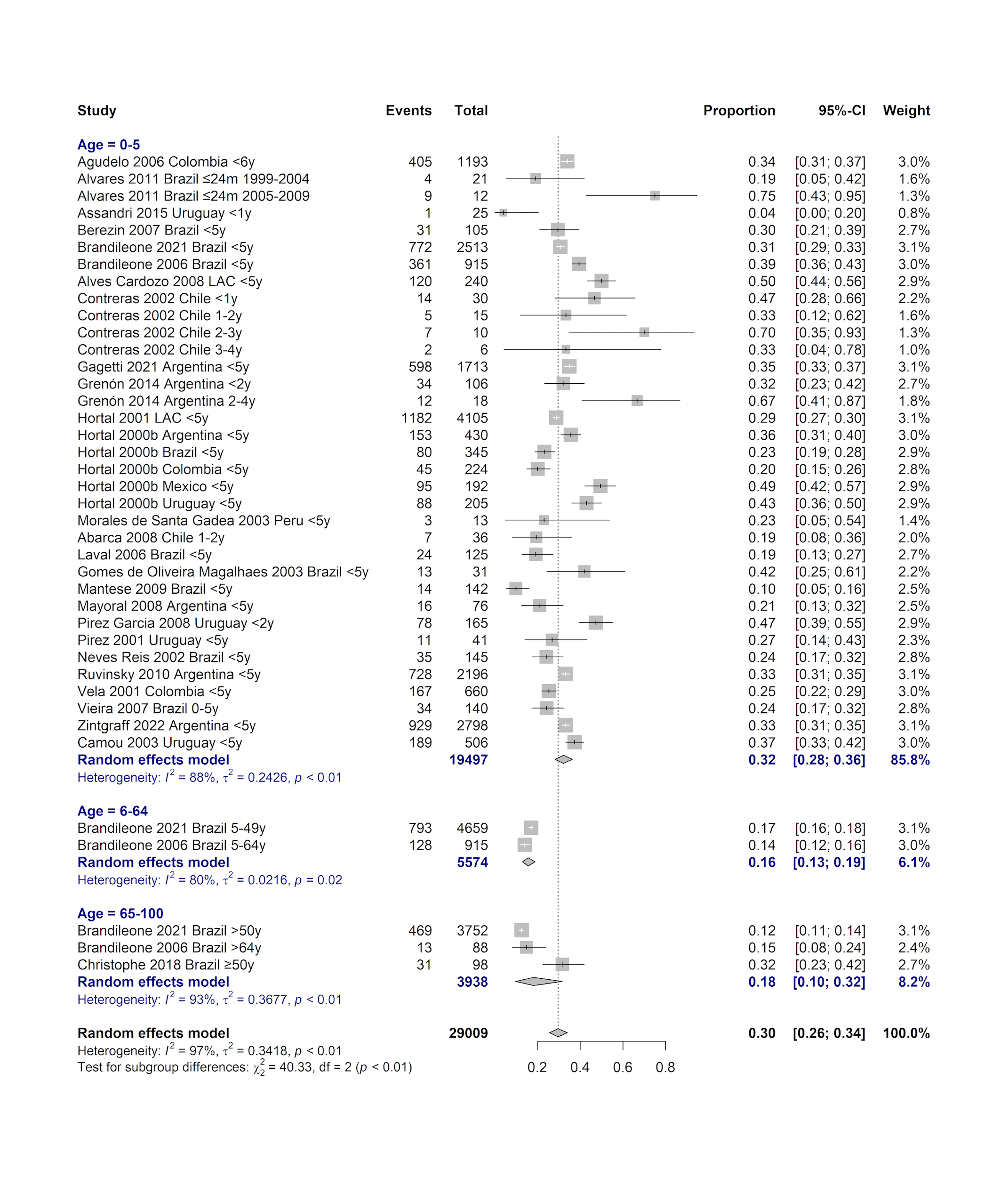


## Supplementary Figure 4. Proportion meta-analysis of resistance to ceftriaxone/cefotaxime


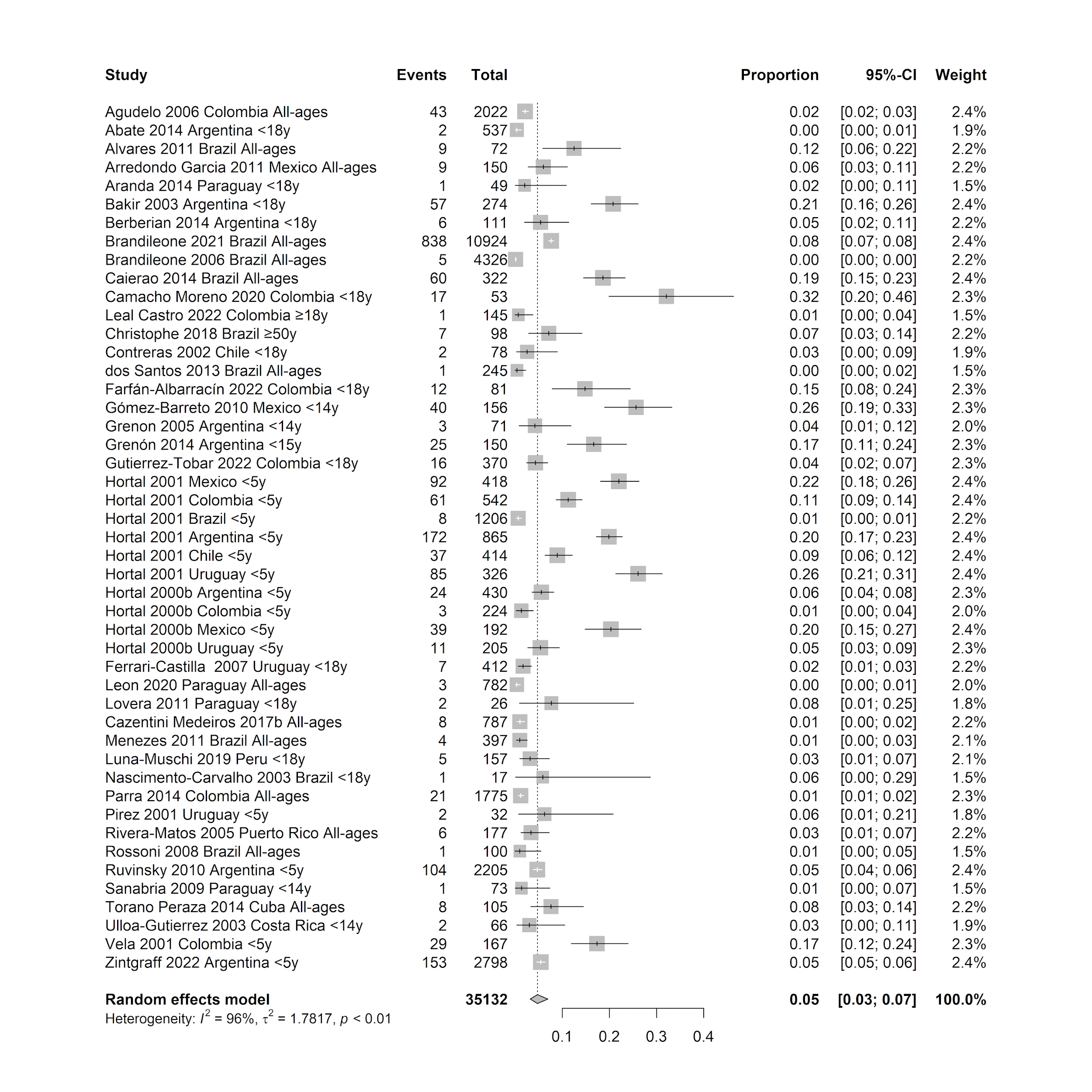


## Supplementary Figure 5. Proportion meta-analysis of resistance to ceftriaxone/cefotaxime by five-year calendar period


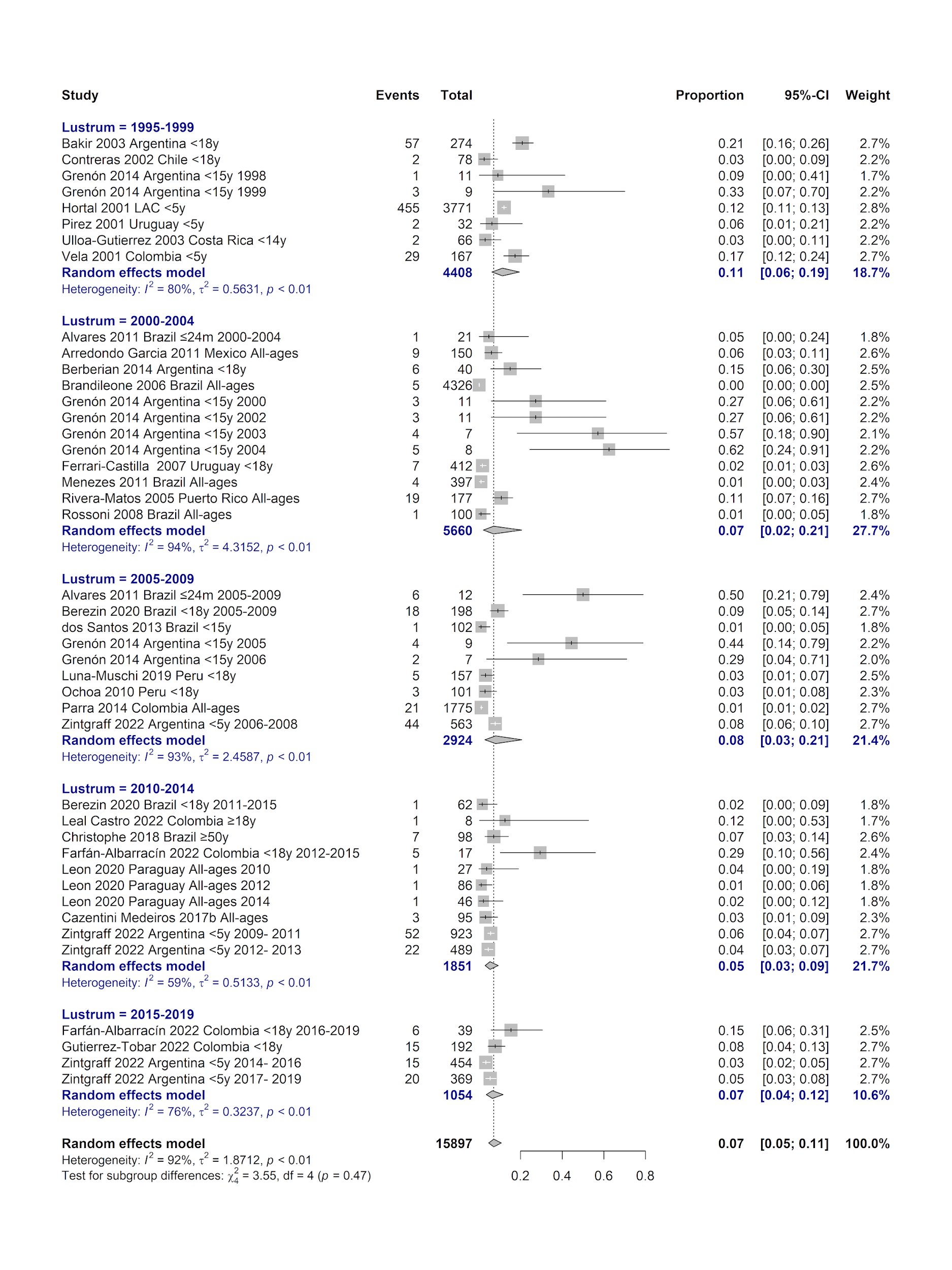


## Supplementary Figure 6. Proportion meta-analysis of resistance to ceftriaxone/cefotaxime by age


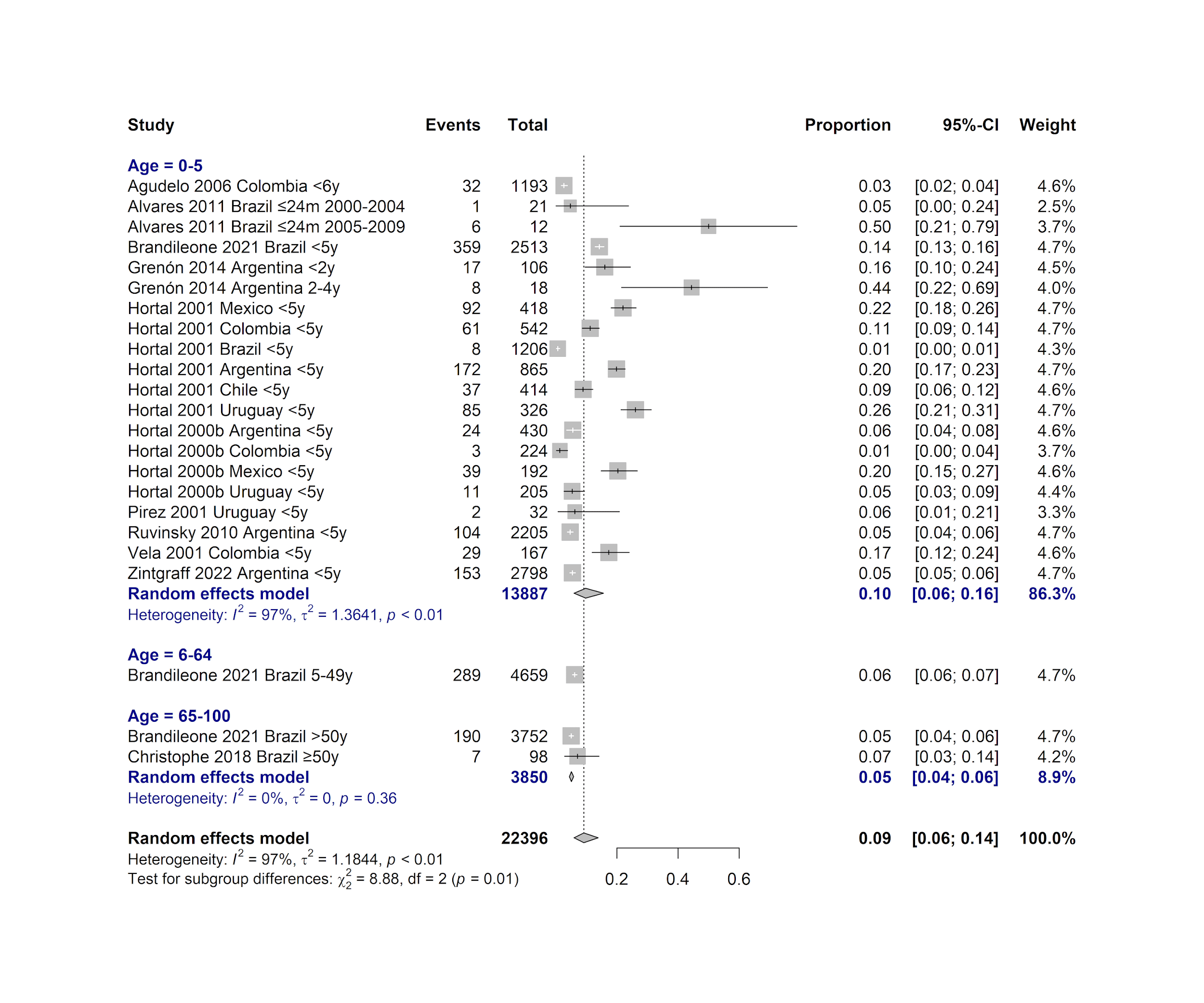


# References of the supplementary material

1. [Abate HJ, Falaschi A, Balbi L, García B. Diecinueve años de vigilancia de enfermedad invasiva neumocócica en un hospital pediátrico de Mendoza, Argentina. Arch Argent Pediatr [Internet]. 2014 Aug 1;112(4). Available from:](http://paperpile.com/b/sdFrri/eLUh) <http://www.sap.org.ar/docs/publicaciones/archivosarg/2014/v112n4a11.pdf>

2. [Altclas J, Salgueira C, Di Martino A. Pneumococcal bacteremia in a single center in Argentina. Int J Infect Dis [Internet]. 2004 Jan;8(1):53–8. Available from:](http://paperpile.com/b/sdFrri/8vUP) <http://dx.doi.org/10.1016/j.ijid.2003.04.002>

3. [Bakir J, de Gentile AS, López H G, Procopio A, Vázquez M. Perfil epidemiológico de las infecciones invasivas por Streptococcus pneumoniae. Rev Chil Pediatr [Internet]. 2003 [cited 2023 Sep 17];74(1):105–13. Available from:](http://paperpile.com/b/sdFrri/Gt8N) <https://www.scielo.cl/scielo.php?pid=S0370-41062003000100015&script=sci_arttext&tlng=pt>

4. [Benitez JD, Martínez ME, Von Specht MH, Gerlach É, Gónzalez CA, Grenón SL. Epidemiología y factores de riesgo de enfermedad invasiva neumocócica en pediatría: Estudio descriptivo, postvacunal. Rev cienc tecnol [Internet]. 2017 [cited 2023 Sep 17];(27):4–10. Available from:](http://paperpile.com/b/sdFrri/G7jP) <http://www.scielo.org.ar/scielo.php?script=sci_abstract&pid=S1851-75872017000100001&lng=es&nrm=iso&tlng=en>

5. [Berberian G, Pérez MG, Epelbaum C, Ceinos MDC, Lopardo H, Rosanova MT. Meningitis neumocócica: experiencia de 12 años en un hospital pediátrico, previa a la inmunización universal con vacuna conjugada. Arch Argent Pediatr [Internet]. 2014 Aug 1;112(4). Available from:](http://paperpile.com/b/sdFrri/7hFe) <http://www.sap.org.ar/docs/publicaciones/archivosarg/2014/v112n4a07.pdf>

6. [Fonaroff EG. Neumonía Neumocócica Bacteriémica en el adulto. Rev panam infectol [Internet]. 2014 [cited 2023 Sep 17];79–85. Available from:](http://paperpile.com/b/sdFrri/NKtC) <http://www.revistaapi.com/wp-content/uploads/2014/09/API-vol-16-n.2_mat-A_p.79-85.pdf>

7. [Gagetti P, Faccone D, Reijtman V, Fossati S, Rodriguez M, Veliz O, et al. Characterization of Streptococcus pneumoniae invasive serotype 19A isolates from Argentina (1993-2014). Vaccine [Internet]. 2017 Aug 16;35(35 Pt B):4548–53. Available from:](http://paperpile.com/b/sdFrri/JY3N) <http://dx.doi.org/10.1016/j.vaccine.2017.07.030>

8. [Gagetti P, Lo SW, Hawkins PA, Gladstone RA, Regueira M, Faccone D, et al. Population genetic structure, serotype distribution and antibiotic resistance of Streptococcus pneumoniae causing invasive disease in children in Argentina. Microb Genom [Internet]. 2021 Sep;7(9). Available from:](http://paperpile.com/b/sdFrri/fKg5) <http://dx.doi.org/10.1099/mgen.0.000636>

9. [Gentile JH, Sparo, Mercapide ME, Luna CM. Adult bacteremic pneumococcal pneumonia acquired in the community. A prospective study on 101 patients. Medicina [Internet]. 2003 [cited 2023 Sep 17];63(1). Available from:](http://paperpile.com/b/sdFrri/tzWi) <https://pubmed.ncbi.nlm.nih.gov/12673954/>

10. [Gentile A, Bakir J, Firpo V, Casanueva EV, Ensinck G, Lopez Papucci S, et al. PCV13 vaccination impact: A multicenter study of pneumonia in 10 pediatric hospitals in Argentina. PLoS One [Internet]. 2018 Jul 18;13(7):e0199989. Available from:](http://paperpile.com/b/sdFrri/lekQ) <http://dx.doi.org/10.1371/journal.pone.0199989>

11. [Gentile A, Bakir J, Lucion MF, Juarez MDV, Rapaport S, Areso MS. Community-Acquired Bacteremic Pneumonia in Post-pneumococcal Vaccination Era in a Pediatric Hospital. Open Forum Infect Dis [Internet]. 2018 Nov 26 [cited 2023 Sep 17];5(suppl_1):S451–2. Available from:](http://paperpile.com/b/sdFrri/94Yh) <https://academic.oup.com/ofid/article/5/suppl_1/S451/5206585>

12. [Grenon S, Von Specht M, Corso A, Pace J, Regueira M. Distribución de serotipos y perfiles de sensibilidad a los antimicrobianos de cepas de Streptococcus pneumoniae aisladas en niños en Misiones, Argentina. Enfermedades Infecciosas y Microbiología Clínica [Internet]. 2005 Jan 1;23(1):10–4. Available from:](http://paperpile.com/b/sdFrri/ZVxL) <https://www.sciencedirect.com/science/article/pii/S0213005X0574893X>

13. [Grenón SL, Salvi Grabulosa MC, Regueira MM, Fossati MS, von Specht MH. Meningitis neumocócica en niños menores de 15 años. Dieciséis años de vigilancia epidemiológica en Misiones, Argentina. Revista Argentina de Microbiología [Internet]. 2014 Jan 1;46(1):14–23. Available from:](http://paperpile.com/b/sdFrri/kOSm) <https://www.sciencedirect.com/science/article/pii/S0325754114700422>

14. [Mathurin S, Jaimet C, Agüero A, Moro J, del Pino A, Arosio A, et al. Estudio prospectivo observacional de bacteriemia neumocóccica en adultos. Aspectos clínicos y factores pronósticos [Internet]. 2008 [cited 2023 Sep 17]. Available from:](http://paperpile.com/b/sdFrri/Gx5y) <http://www.cimero.org.ar/Upload/Directos/Revista/fe2ca2Mathurin%20et%20al.pdf>

15. [Mayoral C, Baroni MR, Giani R, Virgolini S, Zurbriggen L, Regueira M. Serotype distribution of Streptococcus pneumoniae isolated from invasive infections at the Hospital de Niños of Santa Fe. Rev Argent Microbiol [Internet]. 2008 Jan 1 [cited 2023 Sep 18];40(1):13–6. Available from:](http://paperpile.com/b/sdFrri/UofQ) <https://europepmc.org/article/med/18669047?client=bot>

16. [Paganini H, Guiñazú JR, Hernández C, Lopardo H, Gonzalez F, Berberian G. Comparative analysis of outcome and clinical features in children with pleural empyema caused by penicillin-nonsusceptible and penicillin-susceptible Streptococcus pneumoniae. Int J Infect Dis [Internet]. 2001;5(2):86–8. Available from:](http://paperpile.com/b/sdFrri/kjnw) <http://dx.doi.org/10.1016/s1201-9712(01)90031-1>

17. [Pérez G, Mastroianni A, Parra A, Casimir L, Reijtman V, Lopardo H, et al. Infecciones invasivas con bacteriemia por Streptococcus pneumoniae en niños: ¿qué pasó en los últimos 5 años? Med Infant [Internet]. 2014 [cited 2023 Sep 18];318–23. Available from:](http://paperpile.com/b/sdFrri/TMwc) <http://www.medicinainfantil.org.ar/images/stories/volumen/2014/xxi_4_318.pdf>

18. [Ruvinsky R, Regueira M, Fossati M, Gagetti P, Pace J, Rodríguez M, et al. Surveillance of invasive in Streptococcus pneumoniae in Argentina 1994–2007: Changes in serotype distribution, serotype coverage of pneumococcal conjugate vaccines and antibiotic resistance. Pediatr Infect Dis J [Internet]. 2015 Jul 28;05(03):263–9. Available from:](http://paperpile.com/b/sdFrri/9ctC) <https://content.iospress.com/articles/journal-of-pediatric-infectious-diseases/jpi00261>

19. [Zintgraff J, Gagetti P, Napoli D, Sanchez Eluchans N, Irazu L, Moscoloni M, et al. Invasive Streptococcus pneumoniae isolates from pediatric population in Argentina for the period 2006-2019. Temporal progression of serotypes distribution and antibiotic resistance. Vaccine [Internet]. 2022 Jan 24;40(3):459–70. Available from:](http://paperpile.com/b/sdFrri/eMKK) <http://dx.doi.org/10.1016/j.vaccine.2021.12.008>

20. [Alvares JR, Mantese OC, Paula A de, Wolkers PCB, Almeida VVP, Almeida SCG, et al. Prevalence of pneumococcal serotypes and resistance to antimicrobial agents in patients with meningitis: ten-year analysis. Braz J Infect Dis [Internet]. 2011 Jan-Feb;15(1):22–7. Available from:](http://paperpile.com/b/sdFrri/U8Hu) <https://www.ncbi.nlm.nih.gov/pubmed/21412585>

21. [Azevedo J, Dos Anjos ES, Cordeiro SM, Dos Santos MS, Escobar EC, Lobo PR, et al. Genetic profiles and antimicrobial resistance of Streptococcus pneumoniae non-PCV10 serotype isolates recovered from meningitis cases in Salvador, Brazil. J Med Microbiol [Internet]. 2016 Oct;65(10):1164–70. Available from:](http://paperpile.com/b/sdFrri/ElfU) <http://dx.doi.org/10.1099/jmm.0.000346>

22. [Barroso DE, Godoy D, Castiñeiras TMPP, Tulenko MM, Rebelo MC, Harrison LH. β-Lactam resistance, serotype distribution, and genotypes of meningitis-causing Streptococcus pneumoniae, Rio de Janeiro, Brazil. Pediatr Infect Dis J [Internet]. 2012 Jan;31(1):30–6. Available from:](http://paperpile.com/b/sdFrri/cMq2) <http://dx.doi.org/10.1097/INF.0b013e31822f8a92>

23. [Bedran MBM, Camargos PAM, Leocádio Filho G, Bedran RM, Najar HC. Susceptibility of Streptococcus pneumoniae to penicillin in the state of Minas Gerais, Brazil from 1997-2004. Braz J Infect Dis [Internet]. 2005 Oct;9(5):390–7. Available from:](http://paperpile.com/b/sdFrri/HesK) <http://dx.doi.org/10.1590/s1413-86702005000500006>

24. [Berezin EN, Falleiros-Carvalho LH, Lopes CR, Sanajotta AT, Brandileone MC, Menegatti S, et al. Pneumococcal meningitis in children: clinical findings, most frequent serotypes and outcome. J Pediatr [Internet]. 2002 Jan [cited 2023 Sep 18];78(1). Available from:](http://paperpile.com/b/sdFrri/n8eS) <https://pubmed.ncbi.nlm.nih.gov/14647807/>

25. [Berezin EN, Cardenuto MD, Ferreira LL, Otsuka M, Guerra ML, Brandileone MCC. Distribution of Streptococcus pneumoniae serotypes in nasopharyngeal carriage and in invasive pneumococcal disease in Sao Paulo, Brazil. Pediatr Infect Dis J [Internet]. 2007 Jul;26(7):643–5. Available from:](http://paperpile.com/b/sdFrri/eojm) <http://dx.doi.org/10.1097/INF.0b013e3180616d0f>

26. [Berezin EN, Jarovsky D, Cardoso MRA, Mantese OC. Invasive pneumococcal disease among hospitalized children in Brazil before and after the introduction of a pneumococcal conjugate vaccine. Vaccine [Internet]. 2020 Feb 11;38(7):1740–5. Available from:](http://paperpile.com/b/sdFrri/hkT3) <http://dx.doi.org/10.1016/j.vaccine.2019.12.038>

27. [Brandileone MCC, Casagrande ST, Guerra MLLS, Zanella RC, Andrade ALSS, Fabio JLD. Increase in numbers of beta-lactam-resistant invasive Streptococcus pneumoniae in Brazil and the impact of conjugate vaccine coverage. J Med Microbiol [Internet]. 2006 May;55(Pt 5):567–74. Available from:](http://paperpile.com/b/sdFrri/Gjpx) <http://dx.doi.org/10.1099/jmm.0.46387-0>

28. [Brandileone MCC, Almeida SCG, Bokermann S, Minamisava R, Berezin EN, Harrison LH, et al. Dynamics of antimicrobial resistance of Streptococcus pneumoniae following PCV10 introduction in Brazil: Nationwide surveillance from 2007 to 2019. Vaccine [Internet]. 2021 May 27;39(23):3207–15. Available from:](http://paperpile.com/b/sdFrri/YLC4) <http://dx.doi.org/10.1016/j.vaccine.2021.02.063>

29. [Caierão J, Hawkins P, Sant’anna FH, da Cunha GR, d’Azevedo PA, McGee L, et al. Serotypes and genotypes of invasive Streptococcus pneumoniae before and after PCV10 implementation in southern Brazil. PLoS One [Internet]. 2014 Oct 30;9(10):e111129. Available from:](http://paperpile.com/b/sdFrri/Xaj2) <http://dx.doi.org/10.1371/journal.pone.0111129>

30. [Cassiolato AP. Características fenotípicas e moleculares de cepas de Streptococcus pneumoniae do sorotipo 19A isoladas de doenças invasivas e de portadores antes e após a introdução da vacina conjugada 10-valente no Brasil [Internet]. 2019 [cited 2023 Sep 17]. Available from:](http://paperpile.com/b/sdFrri/nNa6) <https://fi-admin.bvsalud.org/document/view/jaaue>

31. [Cazentini Medeiros MI, Almeida SCG, Bokermann S, Watanabe E, Guerra MLLS, Andrade D de. Antimicrobial susceptibility of Streptococcus pneumoniae isolated from patients in the northeastern macroregion of São Paulo state, Brazil, 1998-2013. J Bras Patol Med Lab [Internet]. 2017 [cited 2023 Sep 17];53(3):177–82. Available from:](http://paperpile.com/b/sdFrri/9OrS) <https://www.scielo.br/j/jbpml/a/ydP4D5YFchfGg5vcQXxt3JJ/?lang=en>

32. [Christophe BL, Mott M, da Cunha G, Caierão J, D Azevedo P, Dias C. Characterisation of Streptococcus pneumoniae isolates from invasive disease in adults following the introduction of PCV10 in Brazil. J Med Microbiol [Internet]. 2018 May;67(5):687–94. Available from:](http://paperpile.com/b/sdFrri/2zYg) <http://dx.doi.org/10.1099/jmm.0.000717>

33. [da Silva WA, Pinheiro AM, Coutinho LG, Marinho LAC, Lima LFA. Epidemiological profile of acute bacterial meningitis in the state of Rio Grande do Norte, Brazil. Rev Soc Bras Med Trop [Internet]. 2010 Jul-Aug;43(4):455–7. Available from:](http://paperpile.com/b/sdFrri/rr9C) <http://dx.doi.org/10.1590/s0037-86822010000400023>

34. [dos Santos SR, Passadore LF, Takagi EH, Fujii CM, Yoshioka CRM, Gilio AE, et al. Serotype distribution of Streptococcus pneumoniae isolated from patients with invasive pneumococcal disease in Brazil before and after ten-pneumococcal conjugate vaccine implementation. Vaccine [Internet]. 2013 Dec 9;31(51):6150–4. Available from:](http://paperpile.com/b/sdFrri/upvW) <http://dx.doi.org/10.1016/j.vaccine.2013.05.042>

35. [Gomes de Oliveira Magalhães AP, Pinto A da S. Antimicrobial resistance and serotyping of Streptococcus pneumoniae isolated from pediatric patients in Belo Horizonte, MG, Brazil. Braz J Microbiol [Internet]. 2003 Jul [cited 2023 Sep 17];34(3):210–2. Available from:](http://paperpile.com/b/sdFrri/h45Z) <https://www.scielo.br/j/bjm/a/H8bjpy6kPZfY6hn4HYJWNrG/?lang=en>

36. [Gouveia EL, Reis JN, Flannery B, Cordeiro SM, Lima JBT, Pinheiro RM, et al. Clinical outcome of pneumococcal meningitis during the emergence of pencillin-resistant Streptococcus pneumoniae: an observational study. BMC Infect Dis [Internet]. 2011 Nov 21;11:323. Available from:](http://paperpile.com/b/sdFrri/Y73D) <http://dx.doi.org/10.1186/1471-2334-11-323>

37. [Laval CB, de Andrade ALSS, Pimenta FC, de Andrade JG, de Oliveira RM, Silva SA, et al. Serotypes of carriage and invasive isolates of Streptococcus pneumoniae in Brazilian children in the era of pneumococcal vaccines. Clin Microbiol Infect [Internet]. 2006 Jan;12(1):50–5. Available from:](http://paperpile.com/b/sdFrri/Ihlr) <http://dx.doi.org/10.1111/j.1469-0691.2005.01304.x>

38. [Levin AS, Sessegolo JF, Teixeira LM, Barone AA. Factors associated with penicillin-nonsusceptible pneumococcal infections in Brazil. Braz J Med Biol Res [Internet]. 2003 Jun;36(6):807–13. Available from:](http://paperpile.com/b/sdFrri/NDoO) <http://dx.doi.org/10.1590/s0100-879x2003000600017>

39. [Mantese OC, Paula A de, Almeida VVP, Aguiar PADF de, Wolkers PCB, Alvares JR, et al. Prevalence of serotypes and antimicrobial resistance of invasive strains of pneumococcus in children: analysis of 9 years. J Pediatr [Internet]. 2009 Oct 26;85(6):495–502. Available from:](http://paperpile.com/b/sdFrri/9azR) <http://dx.doi.org/10.2223/JPED.1950>

40. [Menezes APDO, Campos LC, dos Santos MS, Azevedo J, Dos Santos RCN, Carvalho M da GS, et al. Serotype distribution and antimicrobial resistance of Streptococcus pneumoniae prior to introduction of the 10-valent pneumococcal conjugate vaccine in Brazil, 2000-2007. Vaccine [Internet]. 2011 Feb 1;29(6):1139–44. Available from:](http://paperpile.com/b/sdFrri/iXfi) <http://dx.doi.org/10.1016/j.vaccine.2010.12.021>

41. [Mott M, Caierão J, Rosa da Cunha G, Rodrigues Perez LR, Matusiak R, Pilger de Oliveira KR, et al. Susceptibility profiles and correlation with pneumococcal serotypes soon after implementation of the 10-valent pneumococcal conjugate vaccine in Brazil. Int J Infect Dis [Internet]. 2014 Mar;20:47–51. Available from:](http://paperpile.com/b/sdFrri/KoBe) <http://dx.doi.org/10.1016/j.ijid.2013.11.009>

42. [Nascimento-Carvalho CM, Freitas-Souza LS, Moreno-Carvalho OA, Alves NN, Caldas RM, Barberino MG, et al. Invasive pneumococcal strains isolated from children and adolescents in Salvador. J Pediatr [Internet]. 2003 Jun [cited 2023 Sep 17];79(3):209–14. Available from:](http://paperpile.com/b/sdFrri/CG71) <https://www.scielo.br/j/jped/a/6gBgMwkQ5MVq57QjjxWfrCq/abstract/?lang=en&format=html>

43. [Neves Reis J, Cordeiro SM, Coppola SJ, Salgado K, Carvalho MGS, Teixeira LM, et al. Population-based survey of antimicrobial susceptibility and serotype distribution of Streptococcus pneumoniae from meningitis patients in Salvador, Brazil. J Clin Microbiol [Internet]. 2002 Jan;40(1):275–7. Available from:](http://paperpile.com/b/sdFrri/vjNv) <http://dx.doi.org/10.1128/JCM.40.1.275-277.2002>

44. [Pinto TCA, Neves FPG, Souza ARV, Oliveira LMA, Costa NS, Castro LFS, et al. Evolution of Penicillin Non-susceptibility Among Streptococcus pneumoniae Isolates Recovered From Asymptomatic Carriage and Invasive Disease Over 25 years in Brazil, 1990-2014. Front Microbiol [Internet]. 2019 Mar 14;10:486. Available from:](http://paperpile.com/b/sdFrri/FVou) <http://dx.doi.org/10.3389/fmicb.2019.00486>

45. [Rocha Dullius C, Zani L, Chatkin JM. Theoretical pneumococcal vaccine coverage: analysis of serotypes isolated from inpatients at a tertiary care hospital. J Bras Pneumol [Internet]. 2018 Jun 25;44(5):361–6. Available from:](http://paperpile.com/b/sdFrri/RYx2) <http://dx.doi.org/10.1590/S1806-37562017000000056>

46. [Rossoni AM de O, Dalla Costa LM, Berto DB, Farah SS, Gelain M, Brandileone MC de C, et al. Acute bacterial meningitis caused by Streptococcus pneumoniae resistant to the antimicrobian agents and their serotypes. Arq Neuropsiquiatr [Internet]. 2008 Sep;66(3A):509–15. Available from:](http://paperpile.com/b/sdFrri/7KDd) <http://dx.doi.org/10.1590/s0004-282x2008000400014>

47. [Soares dos Santos M, Azevedo J, Menezes AP de O, Cordeiro SM, Escobar EC, Lima JB, et al. Temporal trends and clonal diversity of penicillin non-susceptible pneumococci from meningitis cases from 1996 to 2012, in Salvador, Brazil. BMC Infect Dis [Internet]. 2015 Jul 30;15:302. Available from:](http://paperpile.com/b/sdFrri/aRUw) <http://dx.doi.org/10.1186/s12879-015-1049-y>

48. [Vieira AC, Gomes MC, Rolo Filho M, Eudes Filho J, Bello EJM, de Figueiredo RB. Streptococcus pneumoniae: a study of strains isolated from cerebrospinal fluid. J Pediatr [Internet]. 2007 Jan 23;83(1):71–8. Available from:](http://paperpile.com/b/sdFrri/LHye) <http://dx.doi.org/10.2223/JPED.1580>

49. [Yoshioka CRM, Martinez MB, Brandileone MCC, Ragazzi SB, Guerra MLLS, Santos SR, et al. Análise das cepas de Streptococcus pneumoniae causadores de pneumonia invasiva: sorotipos e sensibilidade aos antimicrobianos. J Pediatr [Internet]. 2011 Feb [cited 2023 Sep 17];87(1):70–5. Available from:](http://paperpile.com/b/sdFrri/C2P5) <https://www.scielo.br/j/jped/a/vZRQbX4yWjsDVQ4SkWnzr4k/?lang=pt>

50. [Abarca VK, Vergara FR, Tassara PE, Ibáñez WI, García BC, Potin SM. Infección neumocóccica invasora y neumonía consolidante en lactantes: Un año de vigilancia en tres centros hospitalarios chilenos. Rev Chilena Infectol [Internet]. 2008 Apr;25(2). Available from:](http://paperpile.com/b/sdFrri/MGha) <http://www.scielo.cl/scielo.php?script=sci_arttext&pid=S0716-10182008000200001&lng=en&nrm=iso&tlng=en>

51. [Aguilera R C, González R G, Bello T H, Mella M S, Blamey D R, Chabouty G H, et al. Antimicrobial susceptibility, capsular serotypes and clonal relationship of invasive streptococcus pneumoniae isolates in adult population of the Bio-Bio region, Chile. 2005-2006. Rev Chilena Infectol [Internet]. 2010 Oct;27(5):392–7. Available from:](http://paperpile.com/b/sdFrri/6k4n) <http://dx.doi.org/10.4067/s0716-10182010000600002>

52. [Contreras L, Fica A, Figueroa O, Enríquez N, Urrutia P, Herrera P. Resistance to Streptococcus pneumoniae to penicillin and its association with clinical and epidemiological factors. Rev Med Chil [Internet]. 2002 Jan [cited 2023 Sep 17];130(1). Available from:](http://paperpile.com/b/sdFrri/Ovfi) <https://pubmed.ncbi.nlm.nih.gov/11961958/>

53. [Fica A, Bunster N, Aliaga F, Olivares F, Porte L, Braun S, et al. Bacteremic pneumococcal pneumonia: serotype distribution, antimicrobial susceptibility, severity scores, risk factors, and mortality in a single center in Chile. Braz J Infect Dis [Internet]. 2014 Mar-Apr;18(2):115–23. Available from:](http://paperpile.com/b/sdFrri/9TIL) <http://dx.doi.org/10.1016/j.bjid.2013.06.001>

54. [Rioseco ML, Riquelme R. Bacteremic pneumococcal pneumonia in 45 immunocompromised hospitalized adults. Rev Med Chil [Internet]. 2004 May;132(5):588–94. Available from:](http://paperpile.com/b/sdFrri/WHcR) <http://dx.doi.org/10.4067/s0034-98872004000500008>

55. [Rioseco Z ML, Riquelme O R, Riquelme O M, Inzunza P C, Riquelme D J, Sanhueza R A. Bacteremic pneumococcal pneumonia in adults admitted to a general hospital. Experience in 60 cases. Rev Med Chil [Internet]. 2018 Jul;146(7):839–45. Available from:](http://paperpile.com/b/sdFrri/akuk) <http://dx.doi.org/10.4067/s0034-98872018000700839>

56. [Saldías P F, Díaz P O. Índices predictores de eventos adversos en el adulto inmunocompetente hospitalizado por neumonía neumocóccica adquirida en la comunidad. Rev Chilena Infectol [Internet]. 2011 Aug;28(4):303–9. Available from:](http://paperpile.com/b/sdFrri/rzbx) <http://www.scielo.cl/scielo.php?script=sci_arttext&pid=S0716-10182011000500001&lng=en&nrm=iso&tlng=en>

57. [Agudelo CI, Moreno J, Sanabria OM, Ovalle MV, Di Fabio JL, Castañeda E. Streptococcus pneumoniae: evolución de los serotipos y los patrones de susceptibilidad antimicrobiana en aislamientos invasores en 11 años de vigilancia en colombia (1994 -2004). Biomedica [Internet]. 2006 Jun 1 [cited 2023 Sep 17];26(2):234–49. Available from:](http://paperpile.com/b/sdFrri/pgXM) <https://revistabiomedica.org/index.php/biomedica/article/view/1413>

58. [Camacho Moreno G, Imbachi LF, Leal AL, Moreno VM, Patiño JA, Gutiérrez IF, et al. Emergence of Streptococcus pneumoniae serotype 19A (Spn19A) in the pediatric population in Bogotá, Colombia as the main cause of invasive pneumococcal disease after the introduction of PCV10. Hum Vaccin Immunother [Internet]. 2020 Sep 1;16(9):2300–6. Available from:](http://paperpile.com/b/sdFrri/aLvN) <http://dx.doi.org/10.1080/21645515.2019.1710411>

59. [Farfán-Albarracín JD, Camacho-Moreno G, Leal AL, Patiño J, Coronell W, Gutiérrez IF, et al. Changes in the incidence of acute bacterial meningitis caused by Streptococcus pneumoniae and the implications of serotype replacement in children in Colombia after mass vaccination with PCV10. Front Pediatr [Internet]. 2022 Sep 23;10:1006887. Available from:](http://paperpile.com/b/sdFrri/I8ys) <http://dx.doi.org/10.3389/fped.2022.1006887>

60. [Gutiérrez-Tobar IF, Londoño-Ruiz JP, Mariño-Drews C, Beltrán-Higuera S, Camacho-Moreno G, Leal-Castro AL, et al. Epidemiological characteristics and serotype distribution of culture-confirmed pediatric pneumococcal pneumonia before and after PCV 10 introduction, a multicenter study in Bogota, Colombia, 2008-2019. Vaccine [Internet]. 2022 May 3;40(20):2875–83. Available from:](http://paperpile.com/b/sdFrri/nfFh) <http://dx.doi.org/10.1016/j.vaccine.2022.03.022>

61. [Leal Castro AL, Camacho-Moreno G, Montañez-Ayala A, Varón-Vega F, Alvarez-Rodríguez JC, Valderrama-Beltrán S, et al. Invasive Pneumococcal Disease Characterization in Adults and Subgroups aged < 60 years and ≥ 60 years in Bogota, Colombia. IJID Reg [Internet]. 2022 Jun;3:293–9. Available from:](http://paperpile.com/b/sdFrri/lbs2) <http://dx.doi.org/10.1016/j.ijregi.2022.04.007>

62. [Parra EL, Ramos V, Sanabria O, Moreno J. Serotype and genotype distribution among invasive Streptococcus pneumoniae isolates in Colombia, 2005-2010. PLoS One [Internet]. 2014 Jan 8;9(1):e84993. Available from:](http://paperpile.com/b/sdFrri/XHNB) <http://dx.doi.org/10.1371/journal.pone.0084993>

63. [Parra EL, Duarte C, Rodríguez K, Sanabria O, Moreno J. Frequency and molecular characterization of invasive isolates of Streptococcus pneumoniae serotypes 6C and 6D in Colombia. Enferm Infecc Microbiol Clin [Internet]. 2017 May;35(5):283–6. Available from:](http://paperpile.com/b/sdFrri/e0qM) <http://dx.doi.org/10.1016/j.eimc.2016.01.014>

64. [Vela MC, Fonseca N, Di Fabio JL, Castañeda E. Presence of international multiresistant clones of Streptococcus pneumoniae in Colombia. Microb Drug Resist [Internet]. 2001 Summer;7(2):153–64. Available from:](http://paperpile.com/b/sdFrri/xhhk) <http://dx.doi.org/10.1089/10766290152045020>

65. [Barboza C, Brenes H, Avila-Aguero ML, Avila L, Camacho K. Epidemiology of Bacterial Meningitis in Pediatric Population After the Introduction of Pneumococcal Conjugated Vaccine in Costa Rica. Open Forum Infect Dis [Internet]. 2018 Nov 26 [cited 2023 Sep 17];5(suppl_1):S138–S138. Available from:](http://paperpile.com/b/sdFrri/AGZP) <https://academic.oup.com/ofid/article/5/suppl_1/S138/5207549>

66. [Ulloa-Gutierrez R, Avila-Aguero ML, Herrera ML, Herrera JF, Arguedas A. Invasive pneumococcal disease in Costa Rican children: a seven year survey. Pediatr Infect Dis J [Internet]. 2003 Dec;22(12):1069–74. Available from:](http://paperpile.com/b/sdFrri/1qjo) <http://dx.doi.org/10.1097/01.inf.0000101475.45195.b1>

67. [Vargas-Gutierrez M, Guila-Esquivel E, Vargas-Acuna MT, Ulloa-Gutierrez R, Soto-Martinez ME. Hospitalizations Due To Complicated Pneumonia With Parapneumonic Pleural Effusion Or Empyema In Costa Rican Children: A 9-Year Study. Am J Respir Crit Care Med [Internet]. 2015 [cited 2023 Sep 18];191:1. Available from:](http://paperpile.com/b/sdFrri/IG1I) <https://search.proquest.com/openview/381c57d9c8cab4672c166fd27f6db45f/1?pq-origsite=gscholar&cbl=40575>

68. [Fonseca Hernández M, Martínez Utrera A, Montes de Oca Rivero M, Cardoso Hernández E, Reyes Sebasco A, Llull Tombo CT, et al. Enfermedad neumocócica invasiva en niños menores de 6 años hospitalizados. Rev Cubana Pediatr [Internet]. 2017 [cited 2023 Sep 18];89:133–43. Available from:](http://paperpile.com/b/sdFrri/Isga) <http://scielo.sld.cu/scielo.php?script=sci_abstract&pid=S0034-75312017000500013&lng=es&nrm=iso&tlng=en>

69. [Toraño-Peraza G, Pías-Solis L, Abreu-Capote M, Rodríguez-Ortega M, Dickinson-Meneses F, Varcárcel-Sánchez M. Serotipos y resistencia antimicrobiana de aislamientos meníngeos de Streptococcus pneumoniae. Cuba, 2007-2012. Vaccimonitor [Internet]. 2014 [cited 2023 Sep 18];23(3):117–23. Available from:](http://paperpile.com/b/sdFrri/89UW) <https://www.medigraphic.com/pdfs/vaccimonitor/vcm-2014/vcm143f.pdf>

70. [Elenga N, Sicard S, Cuadro-Alvarez E, Long L, Njuieyon F, Martin E, et al. Pediatric bacterial meningitis in French Guiana. Med Mal Infect [Internet]. 2015 Nov 20;45(11-12):441–5. Available from:](http://paperpile.com/b/sdFrri/eFrI) <http://dx.doi.org/10.1016/j.medmal.2015.10.003>

71. [Trotman H, Olugbuyi O, Barton M, McGregor D, Thomas S. Pneumococcal meningitis in Jamaican children. West Indian Med J [Internet]. 2009 Dec [cited 2023 Sep 18];58(6). Available from:](http://paperpile.com/b/sdFrri/vogd) <https://pubmed.ncbi.nlm.nih.gov/20583688/>

72. [Cardoso MRA, Nascimento-Carvalho CM, Ferrero F, Berezin EN, Ruvinsky R, Camargos PAM, et al. Penicillin-resistant pneumococcus and risk of treatment failure in pneumonia. Arch Dis Child [Internet]. 2008 Mar;93(3):221–5. Available from:](http://paperpile.com/b/sdFrri/D9UG) <http://dx.doi.org/10.1136/adc.2006.111625>

73. [Hortal M, Ruvinsky R, Rossi A, Agudelo CI, Castañeda E, Brandileone C, et al. Impacto de Streptococcus pneumoniae en las neumonías del niño latinoamericano. Rev Panam Salud Publica [Internet]. 2000 Sep [cited 2023 Sep 18];8(3):185–95. Available from:](http://paperpile.com/b/sdFrri/PyRD) <https://scielosp.org/article/ssm/content/raw/?resource_ssm_path=/media/assets/rpsp/v8n3/3027.pdf>

74. [Hortal M, Lovgren M, de la Hoz F, Agudelo CI, Brandileone MC, Camou T, et al. Antibiotic resistance in Streptococcus pneumoniae in six Latin American countries: 1993-1999 surveillance. Microb Drug Resist [Internet]. 2001 Winter;7(4):391–401. Available from:](http://paperpile.com/b/sdFrri/66kj) <http://dx.doi.org/10.1089/10766290152773400>

75. [Moreno J, Duarte C, Cassiolato AP, Chacón GC, Alarcon P, Sánchez J, et al. Molecular characterization of Latin American invasive Streptococcus pneumoniae serotype 19A isolates. Vaccine [Internet]. 2020 Apr 23;38(19):3524–30. Available from:](http://paperpile.com/b/sdFrri/j7Oi) <http://dx.doi.org/10.1016/j.vaccine.2020.03.030>

76. [Arredondo-García JL, Calderón E, Echániz-Aviles G, Soto-Noguerón A, Arzate P, Amabile-Cuevas CF. Serotypes and antibiotic susceptibility of Streptococcus pneumoniae isolates causative of invasive diseases in Mexican children. J Infect Dev Ctries [Internet]. 2011 Mar 2;5(2):119–22. Available from:](http://paperpile.com/b/sdFrri/frXo) <http://dx.doi.org/10.3855/jidc.1348>

77. [Echaniz-Aviles G, Garza-González E, Román-Mancha AL, Morfín-Otero R, Rodríguez-Noriega E, Ayala-Gaytán JJ, et al. Clinical and microbiological characteristics of community-acquired pneumonia associated with Streptococcus pneumoniae in adult patients in Mexico. Rev Argent Microbiol [Internet]. 2019 Jan 8;51(3):234–40. Available from:](http://paperpile.com/b/sdFrri/GrrN) <http://dx.doi.org/10.1016/j.ram.2018.10.002>

78. [Gómez-Barreto D, Espinosa-Monteros LE, López-Enríquez C, Jiménez-Rojas V, Rodríguez-Suárez R. Invasive pneumococcal disease in a third level pediatric hospital in Mexico City: epidemiology and mortality risk factors. Salud Publica Mex [Internet]. 2010 Sep-Oct;52(5):391–7. Available from:](http://paperpile.com/b/sdFrri/EeOB) <http://dx.doi.org/10.1590/s0036-36342010000500006>

79. [Villaseñor-Sierra A, Lomas-Bautista M, Aguilar-Benavides S, Martínez-Aguilar G. Serotypes and susceptibility of Streptococcus pneumoniae strains isolated from children in Mexico. Salud Publica Mex [Internet]. 2008 Jul-Aug;50(4):330–3. Available from:](http://paperpile.com/b/sdFrri/lBQm) <http://dx.doi.org/10.1590/s0036-36342008000400012>

80. [De León T, Daza C, Cukier G, Chong E, Saldaña R, Samudio Castillo R. Impacto del serotipo 5 en la enfermedad invasora por Streptococcus pneumoniae en la población pediátrica ingresada en el Hospital Materno Infantil José Domingo de Obaldía. Pediátr Panamá [Internet]. 2011 [cited 2023 Sep 18];7–15. Available from:](http://paperpile.com/b/sdFrri/chOl) <http://fi-admin.bvsalud.org/document/view/baes8>

81. [Aranda C, Lovera D, Arbo A. Cambios en el Patrón Epidemiológico y resistencia Bacteriana de la Meningitis Bacteriana Aguda en Niños en un hospital de referencia. Rev Inst Med Trop Sao Paulo [Internet]. 2014 [cited 2023 Sep 17];9(2):10–20. Available from:](http://paperpile.com/b/sdFrri/3kbU) <http://scielo.iics.una.py/scielo.php?script=sci_abstract&pid=S1996-36962014000200003&lng=en&nrm=iso&tlng=es>

82. [León ME, Kawabata A, Nagai M, Rojas L, Zárate N, Irala J, et al. Frecuencia de Streptococcus pneumoniae aislados de enfermedad invasiva en Paraguay, serotipos y perfil de sensibilidad (2010-2018). Mem Inst Investig Cienc Salud [Internet]. 2020 Apr 30;18(1):38–46. Available from:](http://paperpile.com/b/sdFrri/Jc6k) <http://scielo.iics.una.py/scielo.php?script=sci_arttext&pid=S1812-95282020000100038&lng=es&nrm=iso&tlng=es>

83. [Lovera D, Aranda C, Duarte M, Apodaca S, Acuña J, Arbo A. Predicción de la Mortalidad de la Meningitis Neumocóccica en Niños. Pediatr (Asunción) [Internet]. 2011 [cited 2023 Sep 18];38(2):111–7. Available from:](http://paperpile.com/b/sdFrri/hzFG) <http://scielo.iics.una.py/scielo.php?script=sci_abstract&pid=S1683-98032011000200006&lng=en&nrm=iso&tlng=es>

84. [Sanabria G, Araya S, Chamorro G, Lovera D, Arbo A. Correlación de serotipos, sensibilidad y resistencia antimicrobiana en niños con infecciones invasivas por Streptococcus pneumoniae en un centro de referencia de Asunción-Paraguay. Revisión de 6 años. Rev Inst Med Trop Sao Paulo [Internet]. 2009 [cited 2023 Sep 18];4(2):14–24. Available from:](http://paperpile.com/b/sdFrri/15Ol) <http://scielo.iics.una.py/scielo.php?script=sci_abstract&pid=S1996-36962009000200003&lng=en&nrm=iso&tlng=es>

85. [Castro JD, Siccha SM, Egoavil M, Chaparro E, Hernandez R, Silva W, et al. Antibiotic resistance and distribution of serotypes of invasive pneumococcal strains isolated from hospitalized adults in Lima, Peru. Rev Peru Med Exp Salud Publica [Internet]. 2017 Oct-Dec;34(4):633–41. Available from:](http://paperpile.com/b/sdFrri/OFeg) <http://dx.doi.org/10.17843/rpmesp.2017.344.2884>

86. [Castillo-Tokumori F, Mercado E, Marcelo M, Del Aguila O, Reyes I, Campos F, et al. Demographic and clinical characteristics of children with invasive pneumococcal disease in Lima, Peru. In: American Journal of Tropical Medicine and Hygiene. 2018. p. 630–630.](http://paperpile.com/b/sdFrri/NgkE)

87. [Hawkins P, Mercado E, Chochua S, Castillo ME, Reyes I, Chaparro E, et al. Key features of invasive pneumococcal isolates recovered in Lima, Peru determined through whole genome sequencing. Int J Med Microbiol [Internet]. 2017 Oct;307(7):415–21. Available from:](http://paperpile.com/b/sdFrri/aTYC) <http://dx.doi.org/10.1016/j.ijmm.2017.07.008>

88. [Luna-Muschi A, Castillo-Tokumori F, Deza MP, Mercado EH, Egoavil M, Sedano K, et al. Invasive pneumococcal disease in hospitalised children from Lima, Peru before and after introduction of the 7-valent conjugated vaccine. Epidemiol Infect [Internet]. 2019 Jan;147:e91. Available from:](http://paperpile.com/b/sdFrri/pHCt) <http://dx.doi.org/10.1017/S0950268819000037>

89. [Morales de Santa Gadea S. Vigilancia epidemiológica centinela de Haemophilus influenzae y Streptococcus pneumoniae en menores de 5 años en el Perú. Rev Peru Med Exp Salud Publica [Internet]. 2003 [cited 2023 Sep 18];20(3):150–5. Available from:](http://paperpile.com/b/sdFrri/zvqc) <http://www.scielo.org.pe/scielo.php?script=sci_abstract&pid=S1726-46342003000300007&lng=es&nrm=iso&tlng=es>

90. [Rivera-Matos IR, Rios-Olivares E. A multicenter hospital surveillance of invasive Streptococcus pneumoniae, Puerto Rico, 2001. P R Health Sci J [Internet]. 2005 Sep [cited 2023 Sep 18];24(3). Available from:](http://paperpile.com/b/sdFrri/raOq) <https://pubmed.ncbi.nlm.nih.gov/16329681/>

91. [Nurse-Lucas M, McGee L, Hawkins PA, Swanston WH, Akpaka PE. Serotypes and genotypes of Streptococcus pneumoniae isolates from Trinidad and Tobago. Int J Infect Dis [Internet]. 2016 May;46:100–6. Available from:](http://paperpile.com/b/sdFrri/Iijy) <http://dx.doi.org/10.1016/j.ijid.2016.04.005>

92. [Assandri E, Amorín B, Gesuele JP, Algorta G, Pírez MC. Pneumococcal invasive disease in newborns before and after 7-valent and 13-valent universal pneumococcal vaccination in Uruguay. Rev Chilena Infectol [Internet]. 2015 Apr;32(2):167–74. Available from:](http://paperpile.com/b/sdFrri/o0Uc) <http://dx.doi.org/10.4067/S0716-10182015000300005>

93. [Camou T, Palacio R, Di Fabio JL, Hortal M. Invasive pneumococcal diseases in Uruguayan children: comparison between serotype distribution and conjugate vaccine formulations. Vaccine [Internet]. 2003 May 16;21(17-18):2093–6. Available from:](http://paperpile.com/b/sdFrri/EmIJ) <http://dx.doi.org/10.1016/s0264-410x(02)00806-x>

94. [Ferrari Castilla AM, Pirez G MC, Martínez A A, Algorta R G, Chamorro F V, Guala B MJ, et al. Etiology of community acquired pneumonia in inpatients children. Uruguay 1998-2004. Rev Chilena Infectol [Internet]. 2007 Feb;24(1):40–7. Available from:](http://paperpile.com/b/sdFrri/c1OH) <http://dx.doi.org/10.4067/s0716-10182007000100006>

95. [Hortal M, Camou T, Palacio R, Dibarboure H, García A. Ten-year review of invasive pneumococcal diseases in children and adults from Uruguay: clinical spectrum, serotypes, and antimicrobial resistance. Int J Infect Dis [Internet]. 2000;4(2):91–5. Available from:](http://paperpile.com/b/sdFrri/JM4M) <http://dx.doi.org/10.1016/s1201-9712(00)90100-0>

96. [Hortal M, Estevan M, Iraola I, De Mucio B. A population-based assessment of the disease burden of consolidated pneumonia in hospitalized children under five years of age. Int J Infect Dis [Internet]. 2007 May;11(3):273–7. Available from:](http://paperpile.com/b/sdFrri/0NoP) <http://dx.doi.org/10.1016/j.ijid.2006.05.006>

97. [Hortal M, Sehabiague G, Camou T, Iraola I, Estevan M, Pujadas M. Pneumococcal pneumonia in hospitalized Uruguayan children and potential prevention with different vaccine formulations. J Pediatr [Internet]. 2008 Jun;152(6):850–3. Available from:](http://paperpile.com/b/sdFrri/XtOj) <http://dx.doi.org/10.1016/j.jpeds.2007.11.008>

98. [Machado K, López A, Pacheco H, Algorta G, Pírez C. Características del empiema paraneumónico luego del inicio de la vacunación antineumocócica: Centro Hospitalario Pereira Rossell, año 2010. Arch Pediatr Urug [Internet]. 2014 [cited 2023 Sep 18];85(4):212–2219. Available from:](http://paperpile.com/b/sdFrri/2DrY) <http://www.scielo.edu.uy/scielo.php?script=sci_abstract&pid=S1688-12492014000400002&lng=es&nrm=iso&tlng=es>

99. [Machado K, Badía F, Assandri E, Gutiérrez C, Motta I, Varela A, et al. Neumonía necrotizante en niños: 10 años de experiencia en un hospital pediátrico de referencia. Arch Pediatr Urug [Internet]. 2020 [cited 2023 Sep 17];91(5):294–302. Available from:](http://paperpile.com/b/sdFrri/3som) <http://www.scielo.edu.uy/scielo.php?script=sci_arttext&pid=S1688-12492020000500294>

100. [Pírez MC, Martínez O, Ferrari AM, Nairac A, Montano A, Rubio I, et al. Standard case management of pneumonia in hospitalized children in Uruguay, 1997 to 1998. Pediatr Infect Dis J [Internet]. 2001 Mar;20(3):283–9. Available from:](http://paperpile.com/b/sdFrri/VEoM) <http://dx.doi.org/10.1097/00006454-200103000-00013>

101. [Pírez MC, Algorta G, Chamorro F, Romero C, Varela A, Cedres A, et al. Changes in hospitalizations for pneumonia after universal vaccination with pneumococcal conjugate vaccines 7/13 valent and haemophilus influenzae type b conjugate vaccine in a Pediatric Referral Hospital in Uruguay. Pediatr Infect Dis J [Internet]. 2014 Jul;33(7):753–9. Available from:](http://paperpile.com/b/sdFrri/YFik) <http://dx.doi.org/10.1097/INF.0000000000000294>

102. [Pírez MC, Mota MI, Giachetto G, Sánchez Varela M, Galazka J, Gutierrez S, et al. Pneumococcal Meningitis Before and After Universal Vaccination With Pneumococcal Conjugate Vaccines 7/13, Impact on Pediatric Hospitalization in Public and Nonpublic Institutions, in Uruguay. Pediatr Infect Dis J [Internet]. 2017 Oct;36(10):1000–1. Available from:](http://paperpile.com/b/sdFrri/pNN0) <http://dx.doi.org/10.1097/INF.0000000000001671>

103. [Pírez García MC, Giachetto Larraz G, Romero Rostagno C, Zabala Chain C, Algorta Rusiñol G, Montano Lotito A, et al. Neumonía neumocócica invasiva en niños de 0 a 24 meses: ¿influye la resistencia bacteriana en la evolución? Anales de Pediatría [Internet]. 2008 Sep 1;69(3):205–9. Available from:](http://paperpile.com/b/sdFrri/dVUs) <https://www.sciencedirect.com/science/article/pii/S1695403308720472>

# References of included studies

1. [Abate HJ, Falaschi A, Balbi L, García B. Diecinueve años de vigilancia de enfermedad invasiva neumocócica en un hospital pediátrico de Mendoza, Argentina. Arch Argent Pediatr 2014;112. https://doi.org/](http://paperpile.com/b/llchss/vF5u)[10.5546/aap.2014.352](http://dx.doi.org/10.5546/aap.2014.352)[.](http://paperpile.com/b/llchss/vF5u)
2. [Altclas J, Salgueira C, Di Martino A. Pneumococcal bacteremia in a single center in Argentina. Int J Infect Dis 2004;8:53–8. https://doi.org/](http://paperpile.com/b/llchss/XO3p)[10.1016/j.ijid.2003.04.002](http://dx.doi.org/10.1016/j.ijid.2003.04.002)[.](http://paperpile.com/b/llchss/XO3p)
3. [Bakir J, de Gentile AS, López H G, Procopio A, Vázquez M. Perfil epidemiológico de las infecciones invasivas por Streptococcus pneumoniae. Rev Chil Pediatr 2003;74:105–13. https://doi.org/](http://paperpile.com/b/llchss/5bdT)[10.4067/S0370-41062003000100015](http://dx.doi.org/10.4067/S0370-41062003000100015)[.](http://paperpile.com/b/llchss/5bdT)
4. [Benitez JD, Martínez ME, Von Specht MH, Gerlach É, Gónzalez CA, Grenón SL. Epidemiología y factores de riesgo de enfermedad invasiva neumocócica en pediatría: Estudio descriptivo, postvacunal. Rev Cienc Tecnol 2017:4–10.](http://paperpile.com/b/llchss/08Gs)
5. [Berberian G, Pérez MG, Epelbaum C, Ceinos MDC, Lopardo H, Rosanova MT. Meningitis neumocócica: experiencia de 12 años en un hospital pediátrico, previa a la inmunización universal con vacuna conjugada. Arch Argent Pediatr 2014;112. https://doi.org/](http://paperpile.com/b/llchss/Yaht)[10.5546/aap.2014.332](http://dx.doi.org/10.5546/aap.2014.332)[.](http://paperpile.com/b/llchss/Yaht)
6. [Fonaroff EG. Neumonía Neumocócica Bacteriémica en el adulto. Rev panam infectol 2014:79–85.](http://paperpile.com/b/llchss/Ou9S)
7. [Gagetti P, Faccone D, Reijtman V, Fossati S, Rodriguez M, Veliz O, et al. Characterization of Streptococcus pneumoniae invasive serotype 19A isolates from Argentina (1993-2014). Vaccine 2017;35:4548–53. https://doi.org/](http://paperpile.com/b/llchss/092N)[10.1016/j.vaccine.2017.07.030](http://dx.doi.org/10.1016/j.vaccine.2017.07.030)[.](http://paperpile.com/b/llchss/092N)
8. [Gagetti P, Lo SW, Hawkins PA, Gladstone RA, Regueira M, Faccone D, et al. Population genetic structure, serotype distribution and antibiotic resistance of Streptococcus pneumoniae causing invasive disease in children in Argentina. Microb Genom 2021;7. https://doi.org/](http://paperpile.com/b/llchss/37vw)[10.1099/mgen.0.000636](http://dx.doi.org/10.1099/mgen.0.000636)[.](http://paperpile.com/b/llchss/37vw)
9. [Gentile JH, Sparo, Mercapide ME, Luna CM. Adult bacteremic pneumococcal pneumonia acquired in the community. A prospective study on 101 patients. Medicina 2003;63.](http://paperpile.com/b/llchss/mieY)
10. [Gentile A, Bakir J, Firpo V, Casanueva EV, Ensinck G, Lopez Papucci S, et al. PCV13 vaccination impact: A multicenter study of pneumonia in 10 pediatric hospitals in Argentina. PLoS One 2018;13:e0199989. https://doi.org/](http://paperpile.com/b/llchss/fgHk)[10.1371/journal.pone.0199989](http://dx.doi.org/10.1371/journal.pone.0199989)[.](http://paperpile.com/b/llchss/fgHk)
11. [Gentile A, Bakir J, Lucion MF, Juarez MDV, Rapaport S, Areso MS. Community-Acquired Bacteremic Pneumonia in Post-pneumococcal Vaccination Era in a Pediatric Hospital. Open Forum Infect Dis 2018;5:S451–2. https://doi.org/](http://paperpile.com/b/llchss/tlC4)[10.1093/ofid/ofy210.1290](http://dx.doi.org/10.1093/ofid/ofy210.1290)[.](http://paperpile.com/b/llchss/tlC4)
12. [Grenon S, Von Specht M, Corso A, Pace J, Regueira M. Distribución de serotipos y perfiles de sensibilidad a los antimicrobianos de cepas de Streptococcus pneumoniae aisladas en niños en Misiones, Argentina. Enfermedades Infecciosas Y Microbiología Clínica 2005;23:10–4. https://doi.org/](http://paperpile.com/b/llchss/I5I5)[10.1157/13070402](http://dx.doi.org/10.1157/13070402)[.](http://paperpile.com/b/llchss/I5I5)
13. [Grenón SL, Salvi Grabulosa MC, Regueira MM, Fossati MS, von Specht MH. Meningitis neumocócica en niños menores de 15 años. Dieciséis años de vigilancia epidemiológica en Misiones, Argentina. Revista Argentina de Microbiología 2014;46:14–23. https://doi.org/](http://paperpile.com/b/llchss/e9BW)[10.1016/S0325-7541(14)70042-2](http://dx.doi.org/10.1016/S0325-7541(14)70042-2)[.](http://paperpile.com/b/llchss/e9BW)
14. [Mathurin S, Jaimet C, Agüero A, Moro J, del Pino A, Arosio A, et al. Estudio prospectivo observacional de bacteriemia neumocóccica en adultos. Aspectos clínicos y factores pronósticos 2008.](http://paperpile.com/b/llchss/kxYY) <http://www.cimero.org.ar/Upload/Directos/Revista/fe2ca2Mathurin%20et%20al.pdf> [(accessed September 17, 2023).](http://paperpile.com/b/llchss/kxYY)
15. [Mayoral C, Baroni MR, Giani R, Virgolini S, Zurbriggen L, Regueira M. Serotype distribution of Streptococcus pneumoniae isolated from invasive infections at the Hospital de Niños of Santa Fe. Rev Argent Microbiol 2008;40:13–6.](http://paperpile.com/b/llchss/GbqB)
16. [Paganini H, Guiñazú JR, Hernández C, Lopardo H, Gonzalez F, Berberian G. Comparative analysis of outcome and clinical features in children with pleural empyema caused by penicillin-nonsusceptible and penicillin-susceptible Streptococcus pneumoniae. Int J Infect Dis 2001;5:86–8. https://doi.org/](http://paperpile.com/b/llchss/LBMj)[10.1016/s1201-9712(01)90031-1](http://dx.doi.org/10.1016/s1201-9712(01)90031-1)[.](http://paperpile.com/b/llchss/LBMj)
17. [Pérez G, Mastroianni A, Parra A, Casimir L, Reijtman V, Lopardo H, et al. Infecciones invasivas con bacteriemia por Streptococcus pneumoniae en niños: ¿qué pasó en los últimos 5 años? Med Infant 2014:318–23.](http://paperpile.com/b/llchss/4TFT)
18. [Ruvinsky R, Regueira M, Fossati M, Gagetti P, Pace J, Rodríguez M, et al. Surveillance of invasive in Streptococcus pneumoniae in Argentina 1994–2007: Changes in serotype distribution, serotype coverage of pneumococcal conjugate vaccines and antibiotic resistance. Pediatr Infect Dis J 2015;05:263–9. https://doi.org/](http://paperpile.com/b/llchss/jvgi)[10.3233/jpi-2010-0261](http://dx.doi.org/10.3233/jpi-2010-0261)[.](http://paperpile.com/b/llchss/jvgi)
19. [Zintgraff J, Gagetti P, Napoli D, Sanchez Eluchans N, Irazu L, Moscoloni M, et al. Invasive Streptococcus pneumoniae isolates from pediatric population in Argentina for the period 2006-2019. Temporal progression of serotypes distribution and antibiotic resistance. Vaccine 2022;40:459–70. https://doi.org/](http://paperpile.com/b/llchss/wrFG)[10.1016/j.vaccine.2021.12.008](http://dx.doi.org/10.1016/j.vaccine.2021.12.008)[.](http://paperpile.com/b/llchss/wrFG)
20. [Alvares JR, Mantese OC, Paula A de, Wolkers PCB, Almeida VVP, Almeida SCG, et al. Prevalence of pneumococcal serotypes and resistance to antimicrobial agents in patients with meningitis: ten-year analysis. Braz J Infect Dis 2011;15:22–7. https://doi.org/](http://paperpile.com/b/llchss/9dLW)[10.1016/S1413-8670(11)70135-0](http://dx.doi.org/10.1016/S1413-8670(11)70135-0)[.](http://paperpile.com/b/llchss/9dLW)
21. [Azevedo J, Dos Anjos ES, Cordeiro SM, Dos Santos MS, Escobar EC, Lobo PR, et al. Genetic profiles and antimicrobial resistance of Streptococcus pneumoniae non-PCV10 serotype isolates recovered from meningitis cases in Salvador, Brazil. J Med Microbiol 2016;65:1164–70. https://doi.org/](http://paperpile.com/b/llchss/vQtv)[10.1099/jmm.0.000346](http://dx.doi.org/10.1099/jmm.0.000346)[.](http://paperpile.com/b/llchss/vQtv)
22. [Barroso DE, Godoy D, Castiñeiras TMPP, Tulenko MM, Rebelo MC, Harrison LH. β-Lactam resistance, serotype distribution, and genotypes of meningitis-causing Streptococcus pneumoniae, Rio de Janeiro, Brazil. Pediatr Infect Dis J 2012;31:30–6. https://doi.org/](http://paperpile.com/b/llchss/4Xix)[10.1097/INF.0b013e31822f8a92](http://dx.doi.org/10.1097/INF.0b013e31822f8a92)[.](http://paperpile.com/b/llchss/4Xix)
23. [Bedran MBM, Camargos PAM, Leocádio Filho G, Bedran RM, Najar HC. Susceptibility of Streptococcus pneumoniae to penicillin in the state of Minas Gerais, Brazil from 1997-2004. Braz J Infect Dis 2005;9:390–7. https://doi.org/](http://paperpile.com/b/llchss/e0aW)[10.1590/s1413-86702005000500006](http://dx.doi.org/10.1590/s1413-86702005000500006)[.](http://paperpile.com/b/llchss/e0aW)
24. [Berezin EN, Falleiros-Carvalho LH, Lopes CR, Sanajotta AT, Brandileone MC, Menegatti S, et al. Pneumococcal meningitis in children: clinical findings, most frequent serotypes and outcome. J Pediatr 2002;78.](http://paperpile.com/b/llchss/EvT7)
25. [Berezin EN, Cardenuto MD, Ferreira LL, Otsuka M, Guerra ML, Brandileone MCC. Distribution of Streptococcus pneumoniae serotypes in nasopharyngeal carriage and in invasive pneumococcal disease in Sao Paulo, Brazil. Pediatr Infect Dis J 2007;26:643–5. https://doi.org/](http://paperpile.com/b/llchss/tp4q)[10.1097/INF.0b013e3180616d0f](http://dx.doi.org/10.1097/INF.0b013e3180616d0f)[.](http://paperpile.com/b/llchss/tp4q)
26. [Berezin EN, Jarovsky D, Cardoso MRA, Mantese OC. Invasive pneumococcal disease among hospitalized children in Brazil before and after the introduction of a pneumococcal conjugate vaccine. Vaccine 2020;38:1740–5. https://doi.org/](http://paperpile.com/b/llchss/oFrH)[10.1016/j.vaccine.2019.12.038](http://dx.doi.org/10.1016/j.vaccine.2019.12.038)[.](http://paperpile.com/b/llchss/oFrH)
27. [Brandileone M-CC, Casagrande ST, Guerra M-LLS, Zanella RC, Andrade A-LSS, Fabio J-LD. Increase in numbers of beta-lactam-resistant invasive Streptococcus pneumoniae in Brazil and the impact of conjugate vaccine coverage. J Med Microbiol 2006;55:567–74. https://doi.org/](http://paperpile.com/b/llchss/CpEo)[10.1099/jmm.0.46387-0](http://dx.doi.org/10.1099/jmm.0.46387-0)[.](http://paperpile.com/b/llchss/CpEo)
28. [Brandileone M-CC, Almeida SCG, Bokermann S, Minamisava R, Berezin EN, Harrison LH, et al. Dynamics of antimicrobial resistance of Streptococcus pneumoniae following PCV10 introduction in Brazil: Nationwide surveillance from 2007 to 2019. Vaccine 2021;39:3207–15. https://doi.org/](http://paperpile.com/b/llchss/UoPN)[10.1016/j.vaccine.2021.02.063](http://dx.doi.org/10.1016/j.vaccine.2021.02.063)[.](http://paperpile.com/b/llchss/UoPN)
29. [Caierão J, Hawkins P, Sant’anna FH, da Cunha GR, d’Azevedo PA, McGee L, et al. Serotypes and genotypes of invasive Streptococcus pneumoniae before and after PCV10 implementation in southern Brazil. PLoS One 2014;9:e111129. https://doi.org/](http://paperpile.com/b/llchss/8RQT)[10.1371/journal.pone.0111129](http://dx.doi.org/10.1371/journal.pone.0111129)[.](http://paperpile.com/b/llchss/8RQT)
30. [Cassiolato AP. Características fenotípicas e moleculares de cepas de Streptococcus pneumoniae do sorotipo 19A isoladas de doenças invasivas e de portadores antes e após a introdução da vacina conjugada 10-valente no Brasil. 2019.](http://paperpile.com/b/llchss/HctA)
31. [Cazentini Medeiros MI, Almeida SCG, Bokermann S, Watanabe E, Guerra MLLS, Andrade D de. Antimicrobial susceptibility of Streptococcus pneumoniae isolated from patients in the northeastern macroregion of São Paulo state, Brazil, 1998-2013. J Bras Patol Med Lab 2017;53:177–82. https://doi.org/](http://paperpile.com/b/llchss/UjOt)[10.5935/1676-2444.20170029](http://dx.doi.org/10.5935/1676-2444.20170029)[.](http://paperpile.com/b/llchss/UjOt)
32. [Christophe BL, Mott M, da Cunha G, Caierão J, D Azevedo P, Dias C. Characterisation of Streptococcus pneumoniae isolates from invasive disease in adults following the introduction of PCV10 in Brazil. J Med Microbiol 2018;67:687–94. https://doi.org/](http://paperpile.com/b/llchss/SQJo)[10.1099/jmm.0.000717](http://dx.doi.org/10.1099/jmm.0.000717)[.](http://paperpile.com/b/llchss/SQJo)
33. [da Silva WA, Pinheiro AM, Coutinho LG, Marinho LAC, Lima LFA. Epidemiological profile of acute bacterial meningitis in the state of Rio Grande do Norte, Brazil. Rev Soc Bras Med Trop 2010;43:455–7. https://doi.org/](http://paperpile.com/b/llchss/64Km)[10.1590/s0037-86822010000400023](http://dx.doi.org/10.1590/s0037-86822010000400023)[.](http://paperpile.com/b/llchss/64Km)
34. [dos Santos SR, Passadore LF, Takagi EH, Fujii CM, Yoshioka CRM, Gilio AE, et al. Serotype distribution of Streptococcus pneumoniae isolated from patients with invasive pneumococcal disease in Brazil before and after ten-pneumococcal conjugate vaccine implementation. Vaccine 2013;31:6150–4. https://doi.org/](http://paperpile.com/b/llchss/sLG6)[10.1016/j.vaccine.2013.05.042](http://dx.doi.org/10.1016/j.vaccine.2013.05.042)[.](http://paperpile.com/b/llchss/sLG6)
35. [Gomes de Oliveira Magalhães AP, Pinto A da S. Antimicrobial resistance and serotyping of Streptococcus pneumoniae isolated from pediatric patients in Belo Horizonte, MG, Brazil. Braz J Microbiol 2003;34:210–2. https://doi.org/](http://paperpile.com/b/llchss/BP03)[10.1590/S1517-83822003000300005](http://dx.doi.org/10.1590/S1517-83822003000300005)[.](http://paperpile.com/b/llchss/BP03)
36. [Gouveia EL, Reis JN, Flannery B, Cordeiro SM, Lima JBT, Pinheiro RM, et al. Clinical outcome of pneumococcal meningitis during the emergence of pencillin-resistant Streptococcus pneumoniae: an observational study. BMC Infect Dis 2011;11:323. https://doi.org/](http://paperpile.com/b/llchss/8hml)[10.1186/1471-2334-11-323](http://dx.doi.org/10.1186/1471-2334-11-323)[.](http://paperpile.com/b/llchss/8hml)
37. [Laval CB, de Andrade ALSS, Pimenta FC, de Andrade JG, de Oliveira RM, Silva SA, et al. Serotypes of carriage and invasive isolates of Streptococcus pneumoniae in Brazilian children in the era of pneumococcal vaccines. Clin Microbiol Infect 2006;12:50–5. https://doi.org/](http://paperpile.com/b/llchss/ymqn)[10.1111/j.1469-0691.2005.01304.x](http://dx.doi.org/10.1111/j.1469-0691.2005.01304.x)[.](http://paperpile.com/b/llchss/ymqn)
38. [Levin AS, Sessegolo JF, Teixeira LM, Barone AA. Factors associated with penicillin-nonsusceptible pneumococcal infections in Brazil. Braz J Med Biol Res 2003;36:807–13. https://doi.org/](http://paperpile.com/b/llchss/YABr)[10.1590/s0100-879x2003000600017](http://dx.doi.org/10.1590/s0100-879x2003000600017)[.](http://paperpile.com/b/llchss/YABr)
39. [Mantese OC, Paula A de, Almeida VVP, Aguiar PADF de, Wolkers PCB, Alvares JR, et al. Prevalence of serotypes and antimicrobial resistance of invasive strains of pneumococcus in children: analysis of 9 years. J Pediatr 2009;85:495–502. https://doi.org/](http://paperpile.com/b/llchss/NZBM)[10.2223/JPED.1950](http://dx.doi.org/10.2223/JPED.1950)[.](http://paperpile.com/b/llchss/NZBM)
40. [Menezes APDO, Campos LC, dos Santos MS, Azevedo J, Dos Santos RCN, Carvalho M da GS, et al. Serotype distribution and antimicrobial resistance of Streptococcus pneumoniae prior to introduction of the 10-valent pneumococcal conjugate vaccine in Brazil, 2000-2007. Vaccine 2011;29:1139–44. https://doi.org/](http://paperpile.com/b/llchss/qQ3C)[10.1016/j.vaccine.2010.12.021](http://dx.doi.org/10.1016/j.vaccine.2010.12.021)[.](http://paperpile.com/b/llchss/qQ3C)
41. [Mott M, Caierão J, Rosa da Cunha G, Rodrigues Perez LR, Matusiak R, Pilger de Oliveira KR, et al. Susceptibility profiles and correlation with pneumococcal serotypes soon after implementation of the 10-valent pneumococcal conjugate vaccine in Brazil. Int J Infect Dis 2014;20:47–51. https://doi.org/](http://paperpile.com/b/llchss/xKGe)[10.1016/j.ijid.2013.11.009](http://dx.doi.org/10.1016/j.ijid.2013.11.009)[.](http://paperpile.com/b/llchss/xKGe)
42. [Nascimento-Carvalho CM, Freitas-Souza LS, Moreno-Carvalho OA, Alves NN, Caldas RM, Barberino MG, et al. Invasive pneumococcal strains isolated from children and adolescents in Salvador. J Pediatr 2003;79:209–14. https://doi.org/](http://paperpile.com/b/llchss/ivS2)[10.1590/S0021-75572003000300005](http://dx.doi.org/10.1590/S0021-75572003000300005)[.](http://paperpile.com/b/llchss/ivS2)
43. [Neves Reis J, Cordeiro SM, Coppola SJ, Salgado K, Carvalho MGS, Teixeira LM, et al. Population-based survey of antimicrobial susceptibility and serotype distribution of Streptococcus pneumoniae from meningitis patients in Salvador, Brazil. J Clin Microbiol 2002;40:275–7. https://doi.org/](http://paperpile.com/b/llchss/z25v)[10.1128/JCM.40.1.275-277.2002](http://dx.doi.org/10.1128/JCM.40.1.275-277.2002)[.](http://paperpile.com/b/llchss/z25v)
44. [Pinto TCA, Neves FPG, Souza ARV, Oliveira LMA, Costa NS, Castro LFS, et al. Evolution of Penicillin Non-susceptibility Among Streptococcus pneumoniae Isolates Recovered From Asymptomatic Carriage and Invasive Disease Over 25 years in Brazil, 1990-2014. Front Microbiol 2019;10:486. https://doi.org/](http://paperpile.com/b/llchss/vniQ)[10.3389/fmicb.2019.00486](http://dx.doi.org/10.3389/fmicb.2019.00486)[.](http://paperpile.com/b/llchss/vniQ)
45. [Rocha Dullius C, Zani L, Chatkin JM. Theoretical pneumococcal vaccine coverage: analysis of serotypes isolated from inpatients at a tertiary care hospital. J Bras Pneumol 2018;44:361–6. https://doi.org/](http://paperpile.com/b/llchss/KSJV)[10.1590/S1806-37562017000000056](http://dx.doi.org/10.1590/S1806-37562017000000056)[.](http://paperpile.com/b/llchss/KSJV)
46. [Rossoni AM de O, Dalla Costa LM, Berto DB, Farah SS, Gelain M, Brandileone MC de C, et al. Acute bacterial meningitis caused by Streptococcus pneumoniae resistant to the antimicrobian agents and their serotypes. Arq Neuropsiquiatr 2008;66:509–15. https://doi.org/](http://paperpile.com/b/llchss/CIBf)[10.1590/s0004-282x2008000400014](http://dx.doi.org/10.1590/s0004-282x2008000400014)[.](http://paperpile.com/b/llchss/CIBf)
47. [Soares dos Santos M, Azevedo J, Menezes AP de O, Cordeiro SM, Escobar EC, Lima JB, et al. Temporal trends and clonal diversity of penicillin non-susceptible pneumococci from meningitis cases from 1996 to 2012, in Salvador, Brazil. BMC Infect Dis 2015;15:302. https://doi.org/](http://paperpile.com/b/llchss/Txd9)[10.1186/s12879-015-1049-y](http://dx.doi.org/10.1186/s12879-015-1049-y)[.](http://paperpile.com/b/llchss/Txd9)
48. [Vieira AC, Gomes MC, Rolo Filho M, Eudes Filho J, Bello EJM, de Figueiredo RB. Streptococcus pneumoniae: a study of strains isolated from cerebrospinal fluid. J Pediatr 2007;83:71–8. https://doi.org/](http://paperpile.com/b/llchss/TRyK)[10.2223/JPED.1580](http://dx.doi.org/10.2223/JPED.1580)[.](http://paperpile.com/b/llchss/TRyK)
49. [Yoshioka CRM, Martinez MB, Brandileone MCC, Ragazzi SB, Guerra MLLS, Santos SR, et al. Análise das cepas de Streptococcus pneumoniae causadores de pneumonia invasiva: sorotipos e sensibilidade aos antimicrobianos. J Pediatr 2011;87:70–5. https://doi.org/](http://paperpile.com/b/llchss/fSmp)[10.1590/S0021-75572011000100012](http://dx.doi.org/10.1590/S0021-75572011000100012)[.](http://paperpile.com/b/llchss/fSmp)
50. [Abarca VK, Vergara FR, Tassara PE, Ibáñez WI, García BC, Potin SM. Infección neumocóccica invasora y neumonía consolidante en lactantes: Un año de vigilancia en tres centros hospitalarios chilenos. Rev Chilena Infectol 2008;25. https://doi.org/](http://paperpile.com/b/llchss/pMRT)[10.4067/s0716-10182008000200001](http://dx.doi.org/10.4067/s0716-10182008000200001)[.](http://paperpile.com/b/llchss/pMRT)
51. [Aguilera R C, González R G, Bello T H, Mella M S, Blamey D R, Chabouty G H, et al. Antimicrobial susceptibility, capsular serotypes and clonal relationship of invasive streptococcus pneumoniae isolates in adult population of the Bio-Bio region, Chile. 2005-2006. Rev Chilena Infectol 2010;27:392–7. https://doi.org/](http://paperpile.com/b/llchss/ZvlN)[10.4067/s0716-10182010000600002](http://dx.doi.org/10.4067/s0716-10182010000600002)[.](http://paperpile.com/b/llchss/ZvlN)
52. [Contreras L, Fica A, Figueroa O, Enríquez N, Urrutia P, Herrera P. Resistance to Streptococcus pneumoniae to penicillin and its association with clinical and epidemiological factors. Rev Med Chil 2002;130.](http://paperpile.com/b/llchss/ch6h)
53. [Fica A, Bunster N, Aliaga F, Olivares F, Porte L, Braun S, et al. Bacteremic pneumococcal pneumonia: serotype distribution, antimicrobial susceptibility, severity scores, risk factors, and mortality in a single center in Chile. Braz J Infect Dis 2014;18:115–23. https://doi.org/](http://paperpile.com/b/llchss/ht5O)[10.1016/j.bjid.2013.06.001](http://dx.doi.org/10.1016/j.bjid.2013.06.001)[.](http://paperpile.com/b/llchss/ht5O)
54. [Rioseco ML, Riquelme R. Bacteremic pneumococcal pneumonia in 45 immunocompromised hospitalized adults. Rev Med Chil 2004;132:588–94. https://doi.org/](http://paperpile.com/b/llchss/odZo)[10.4067/s0034-98872004000500008](http://dx.doi.org/10.4067/s0034-98872004000500008)[.](http://paperpile.com/b/llchss/odZo)
55. [Rioseco Z ML, Riquelme O R, Riquelme O M, Inzunza P C, Riquelme D J, Sanhueza R A. Bacteremic pneumococcal pneumonia in adults admitted to a general hospital. Experience in 60 cases. Rev Med Chil 2018;146:839–45. https://doi.org/](http://paperpile.com/b/llchss/l9Ui)[10.4067/s0034-98872018000700839](http://dx.doi.org/10.4067/s0034-98872018000700839)[.](http://paperpile.com/b/llchss/l9Ui)
56. [Saldías P F, Díaz P O. Índices predictores de eventos adversos en el adulto inmunocompetente hospitalizado por neumonía neumocóccica adquirida en la comunidad. Rev Chilena Infectol 2011;28:303–9. https://doi.org/](http://paperpile.com/b/llchss/cwj6)[10.4067/s0716-10182011000500001](http://dx.doi.org/10.4067/s0716-10182011000500001)[.](http://paperpile.com/b/llchss/cwj6)
57. [Agudelo CI, Moreno J, Sanabria OM, Ovalle MV, Di Fabio JL, Castañeda E. Streptococcus pneumoniae: evolución de los serotipos y los patrones de susceptibilidad antimicrobiana en aislamientos invasores en 11 años de vigilancia en colombia (1994 -2004). Biomedica 2006;26:234–49. https://doi.org/](http://paperpile.com/b/llchss/SagI)[10.7705/biomedica.v26i2.1413](http://dx.doi.org/10.7705/biomedica.v26i2.1413)[.](http://paperpile.com/b/llchss/SagI)
58. [Camacho Moreno G, Imbachi LF, Leal AL, Moreno VM, Patiño JA, Gutiérrez IF, et al. Emergence of Streptococcus pneumoniae serotype 19A (Spn19A) in the pediatric population in Bogotá, Colombia as the main cause of invasive pneumococcal disease after the introduction of PCV10. Hum Vaccin Immunother 2020;16:2300–6. https://doi.org/](http://paperpile.com/b/llchss/Ud00)[10.1080/21645515.2019.1710411](http://dx.doi.org/10.1080/21645515.2019.1710411)[.](http://paperpile.com/b/llchss/Ud00)
59. [Farfán-Albarracín JD, Camacho-Moreno G, Leal AL, Patiño J, Coronell W, Gutiérrez IF, et al. Changes in the incidence of acute bacterial meningitis caused by Streptococcus pneumoniae and the implications of serotype replacement in children in Colombia after mass vaccination with PCV10. Front Pediatr 2022;10:1006887. https://doi.org/](http://paperpile.com/b/llchss/jinB)[10.3389/fped.2022.1006887](http://dx.doi.org/10.3389/fped.2022.1006887)[.](http://paperpile.com/b/llchss/jinB)
60. [Gutiérrez-Tobar IF, Londoño-Ruiz JP, Mariño-Drews C, Beltrán-Higuera S, Camacho-Moreno G, Leal-Castro AL, et al. Epidemiological characteristics and serotype distribution of culture-confirmed pediatric pneumococcal pneumonia before and after PCV 10 introduction, a multicenter study in Bogota, Colombia, 2008-2019. Vaccine 2022;40:2875–83. https://doi.org/](http://paperpile.com/b/llchss/LJrt)[10.1016/j.vaccine.2022.03.022](http://dx.doi.org/10.1016/j.vaccine.2022.03.022)[.](http://paperpile.com/b/llchss/LJrt)
61. [Leal Castro AL, Camacho-Moreno G, Montañez-Ayala A, Varón-Vega F, Alvarez-Rodríguez JC, Valderrama-Beltrán S, et al. Invasive Pneumococcal Disease Characterization in Adults and Subgroups aged < 60 years and ≥ 60 years in Bogota, Colombia. IJID Reg 2022;3:293–9. https://doi.org/](http://paperpile.com/b/llchss/FY3n)[10.1016/j.ijregi.2022.04.007](http://dx.doi.org/10.1016/j.ijregi.2022.04.007).
62. [Parra EL, Ramos V, Sanabria O, Moreno J. Serotype and genotype distribution among invasive Streptococcus pneumoniae isolates in Colombia, 2005-2010. PLoS One 2014;9:e84993. https://doi.org/](http://paperpile.com/b/llchss/TeOJ)[10.1371/journal.pone.0084993](http://dx.doi.org/10.1371/journal.pone.0084993)[.](http://paperpile.com/b/llchss/TeOJ)
63. [Parra EL, Duarte C, Rodríguez K, Sanabria O, Moreno J. Frequency and molecular characterization of invasive isolates of Streptococcus pneumoniae serotypes 6C and 6D in Colombia. Enferm Infecc Microbiol Clin 2017;35:283–6. https://doi.org/](http://paperpile.com/b/llchss/fR40)[10.1016/j.eimc.2016.01.014](http://dx.doi.org/10.1016/j.eimc.2016.01.014)[.](http://paperpile.com/b/llchss/fR40)
64. [Vela MC, Fonseca N, Di Fabio JL, Castañeda E. Presence of international multiresistant clones of Streptococcus pneumoniae in Colombia. Microb Drug Resist 2001;7:153–64. https://doi.org/](http://paperpile.com/b/llchss/AvRf)[10.1089/10766290152045020](http://dx.doi.org/10.1089/10766290152045020)[.](http://paperpile.com/b/llchss/AvRf)
65. [Barboza C, Brenes H, Avila-Aguero ML, Avila L, Camacho K. Epidemiology of Bacterial Meningitis in Pediatric Population After the Introduction of Pneumococcal Conjugated Vaccine in Costa Rica. Open Forum Infect Dis 2018;5:S138–S138. https://doi.org/](http://paperpile.com/b/llchss/GMLA)[10.1093/ofid/ofy210.361](http://dx.doi.org/10.1093/ofid/ofy210.361)[.](http://paperpile.com/b/llchss/GMLA)
66. [Ulloa-Gutierrez R, Avila-Aguero ML, Herrera ML, Herrera JF, Arguedas A. Invasive pneumococcal disease in Costa Rican children: a seven year survey. Pediatr Infect Dis J 2003;22:1069–74. https://doi.org/](http://paperpile.com/b/llchss/mBNa)[10.1097/01.inf.0000101475.45195.b1](http://dx.doi.org/10.1097/01.inf.0000101475.45195.b1)[.](http://paperpile.com/b/llchss/mBNa)
67. [Vargas-Gutierrez M, Guila-Esquivel E, Vargas-Acuna MT, Ulloa-Gutierrez R, Soto-Martinez ME. Hospitalizations Due To Complicated Pneumonia With Parapneumonic Pleural Effusion Or Empyema In Costa Rican Children: A 9-Year Study. Am J Respir Crit Care Med 2015;191:1.](http://paperpile.com/b/llchss/Q62m)
68. [Fonseca Hernández M, Martínez Utrera A, Montes de Oca Rivero M, Cardoso Hernández E, Reyes Sebasco A, Llull Tombo CT, et al. Enfermedad neumocócica invasiva en niños menores de 6 años hospitalizados. Rev Cubana Pediatr 2017;89:133–43.](http://paperpile.com/b/llchss/teW1)
69. [Toraño-Peraza G, Pías-Solis L, Abreu-Capote M, Rodríguez-Ortega M, Dickinson-Meneses F, Varcárcel-Sánchez M. Serotipos y resistencia antimicrobiana de aislamientos meníngeos de Streptococcus pneumoniae. Cuba, 2007-2012. Vaccimonitor 2014;23:117–23.](http://paperpile.com/b/llchss/FOA3)
70. [Elenga N, Sicard S, Cuadro-Alvarez E, Long L, Njuieyon F, Martin E, et al. Pediatric bacterial meningitis in French Guiana. Med Mal Infect 2015;45:441–5. https://doi.org/](http://paperpile.com/b/llchss/5C8L)[10.1016/j.medmal.2015.10.003](http://dx.doi.org/10.1016/j.medmal.2015.10.003)[.](http://paperpile.com/b/llchss/5C8L)
71. [Trotman H, Olugbuyi O, Barton M, McGregor D, Thomas S. Pneumococcal meningitis in Jamaican children. West Indian Med J 2009;58.](http://paperpile.com/b/llchss/yaVe)
72. [Cardoso MRA, Nascimento-Carvalho CM, Ferrero F, Berezin EN, Ruvinsky R, Camargos PAM, et al. Penicillin-resistant pneumococcus and risk of treatment failure in pneumonia. Arch Dis Child 2008;93:221–5. https://doi.org/](http://paperpile.com/b/llchss/7sxX)[10.1136/adc.2006.111625](http://dx.doi.org/10.1136/adc.2006.111625)[.](http://paperpile.com/b/llchss/7sxX)
73. [Hortal M, Ruvinsky R, Rossi A, Agudelo CI, Castañeda E, Brandileone C, et al. Impacto de Streptococcus pneumoniae en las neumonías del niño latinoamericano. Rev Panam Salud Publica 2000;8:185–95.](http://paperpile.com/b/llchss/rVks)
74. [Hortal M, Lovgren M, de la Hoz F, Agudelo CI, Brandileone MC, Camou T, et al. Antibiotic resistance in Streptococcus pneumoniae in six Latin American countries: 1993-1999 surveillance. Microb Drug Resist 2001;7:391–401. https://doi.org/](http://paperpile.com/b/llchss/ejNM)[10.1089/10766290152773400](http://dx.doi.org/10.1089/10766290152773400)[.](http://paperpile.com/b/llchss/ejNM)
75. [Moreno J, Duarte C, Cassiolato AP, Chacón GC, Alarcon P, Sánchez J, et al. Molecular characterization of Latin American invasive Streptococcus pneumoniae serotype 19A isolates. Vaccine 2020;38:3524–30. https://doi.org/](http://paperpile.com/b/llchss/q9Mr)[10.1016/j.vaccine.2020.03.030](http://dx.doi.org/10.1016/j.vaccine.2020.03.030)[.](http://paperpile.com/b/llchss/q9Mr)
76. [Arredondo-García JL, Calderón E, Echániz-Aviles G, Soto-Noguerón A, Arzate P, Amabile-Cuevas CF. Serotypes and antibiotic susceptibility of Streptococcus pneumoniae isolates causative of invasive diseases in Mexican children. J Infect Dev Ctries 2011;5:119–22. https://doi.org/](http://paperpile.com/b/llchss/sagR)[10.3855/jidc.1348](http://dx.doi.org/10.3855/jidc.1348)[.](http://paperpile.com/b/llchss/sagR)
77. [Echaniz-Aviles G, Garza-González E, Román-Mancha AL, Morfín-Otero R, Rodríguez-Noriega E, Ayala-Gaytán JJ, et al. Clinical and microbiological characteristics of community-acquired pneumonia associated with Streptococcus pneumoniae in adult patients in Mexico. Rev Argent Microbiol 2019;51:234–40. https://doi.org/](http://paperpile.com/b/llchss/zGfi)[10.1016/j.ram.2018.10.002](http://dx.doi.org/10.1016/j.ram.2018.10.002)[.](http://paperpile.com/b/llchss/zGfi)
78. [Gómez-Barreto D, Espinosa-Monteros LE, López-Enríquez C, Jiménez-Rojas V, Rodríguez-Suárez R. Invasive pneumococcal disease in a third level pediatric hospital in Mexico City: epidemiology and mortality risk factors. S](http://paperpile.com/b/llchss/9w9D)
79. [Villaseñor-Sierra A, Lomas-Bautista M, Aguilar-Benavides S, Martínez-Aguilar G. Serotypes and susceptibility of Streptococcus pneumoniae strains isolated from children in Mexico. Salud Publica Mex 2008;50:330–3. https://doi.org/](http://paperpile.com/b/llchss/tW5o)[10.1590/s0036-36342008000400012](http://dx.doi.org/10.1590/s0036-36342008000400012)[.](http://paperpile.com/b/llchss/tW5o)
80. [De León T, Daza C, Cukier G, Chong E, Saldaña R, Samudio Castillo R. Impacto del serotipo 5 en la enfermedad invasora por Streptococcus pneumoniae en la población pediátrica ingresada en el Hospital Materno Infantil José Domingo de Obaldía. Pediátr Panamá 2011:7–15.](http://paperpile.com/b/llchss/I7Yt)
81. [Aranda C, Lovera D, Arbo A. Cambios en el Patrón Epidemiológico y resistencia Bacteriana de la Meningitis Bacteriana Aguda en Niños en un hospital de referencia. Rev Inst Med Trop Sao Paulo 2014;9:10–20.](http://paperpile.com/b/llchss/1xKS)
82. [León ME, Kawabata A, Nagai M, Rojas L, Zárate N, Irala J, et al. Frecuencia de Streptococcus pneumoniae aislados de enfermedad invasiva en Paraguay, serotipos y perfil de sensibilidad (2010-2018). Mem Inst Investig Cienc Salud 2020;18:38–46. https://doi.org/](http://paperpile.com/b/llchss/B14I)[10.18004/mem.iics/1812-9528/2020.018.01.38-046](http://dx.doi.org/10.18004/mem.iics/1812-9528/2020.018.01.38-046)[.](http://paperpile.com/b/llchss/B14I)
83. [Lovera D, Aranda C, Duarte M, Apodaca S, Acuña J, Arbo A. Predicción de la Mortalidad de la Meningitis Neumocóccica en Niños. Pediatr (Asunción) 2011;38:111–7.](http://paperpile.com/b/llchss/3wDU)
84. [Sanabria G, Araya S, Chamorro G, Lovera D, Arbo A. Correlación de serotipos, sensibilidad y resistencia antimicrobiana en niños con infecciones invasivas por Streptococcus pneumoniae en un centro de referencia de Asunción-Paraguay. Revisión de 6 años. Rev Inst Med Trop Sao Paulo 2009;4:14–24.](http://paperpile.com/b/llchss/rf6X)
85. [Castro JD, Siccha SM, Egoavil M, Chaparro E, Hernandez R, Silva W, et al. Antibiotic resistance and distribution of serotypes of invasive pneumococcal strains isolated from hospitalized adults in Lima, Peru. Rev Peru Med Exp Salud Publica 2017;34:633–41. https://doi.org/](http://paperpile.com/b/llchss/1eFu)[10.17843/rpmesp.2017.344.2884](http://dx.doi.org/10.17843/rpmesp.2017.344.2884)[.](http://paperpile.com/b/llchss/1eFu)
86. [Castillo-Tokumori F, Mercado E, Marcelo M, Del Aguila O, Reyes I, Campos F, et al. Demographic and clinical characteristics of children with invasive pneumococcal disease in Lima, Peru. American Journal of Tropical Medicine and Hygiene, vol. 99, 2018, p. 630–630.](http://paperpile.com/b/llchss/JpSZ)
87. [Hawkins P, Mercado E, Chochua S, Castillo ME, Reyes I, Chaparro E, et al. Key features of invasive pneumococcal isolates recovered in Lima, Peru determined through whole genome sequencing. Int J Med Microbiol 2017;307:415–21. https://doi.org/](http://paperpile.com/b/llchss/qhZm)[10.1016/j.ijmm.2017.07.008](http://dx.doi.org/10.1016/j.ijmm.2017.07.008)[.](http://paperpile.com/b/llchss/qhZm)
88. [Luna-Muschi A, Castillo-Tokumori F, Deza MP, Mercado EH, Egoavil M, Sedano K, et al. Invasive pneumococcal disease in hospitalised children from Lima, Peru before and after introduction of the 7-valent conjugated vaccine. Epidemiol Infect 2019;147:e91. https://doi.org/](http://paperpile.com/b/llchss/GMpf)[10.1017/S0950268819000037](http://dx.doi.org/10.1017/S0950268819000037)[.](http://paperpile.com/b/llchss/GMpf)
89. [Morales de Santa Gadea S. Vigilancia epidemiológica centinela de Haemophilus influenzae y Streptococcus pneumoniae en menores de 5 años en el Perú. Rev Peru Med Exp Salud Publica 2003;20:150–5.](http://paperpile.com/b/llchss/9plJ)
90. [Rivera-Matos IR, Rios-Olivares E. A multicenter hospital surveillance of invasive Streptococcus pneumoniae, Puerto Rico, 2001. P R Health Sci J 2005;24.](http://paperpile.com/b/llchss/SQ9E)
91. [Nurse-Lucas M, McGee L, Hawkins PA, Swanston WH, Akpaka PE. Serotypes and genotypes of Streptococcus pneumoniae isolates from Trinidad and Tobago. Int J Infect Dis 2016;46:100–6. https://doi.org/](http://paperpile.com/b/llchss/pWie)[10.1016/j.ijid.2016.04.005](http://dx.doi.org/10.1016/j.ijid.2016.04.005)[.](http://paperpile.com/b/llchss/pWie)
92. [Assandri E, Amorín B, Gesuele JP, Algorta G, Pírez MC. Pneumococcal invasive disease in newborns before and after 7-valent and 13-valent universal pneumococcal vaccination in Uruguay. Rev Chilena Infectol 2015;32:167–74. https://doi.org/](http://paperpile.com/b/llchss/Qztd)[10.4067/S0716-10182015000300005](http://dx.doi.org/10.4067/S0716-10182015000300005)[.](http://paperpile.com/b/llchss/Qztd)
93. [Camou T, Palacio R, Di Fabio JL, Hortal M. Invasive pneumococcal diseases in Uruguayan children: comparison between serotype distribution and conjugate vaccine formulations. Vaccine 2003;21:2093–6. https://doi.org/](http://paperpile.com/b/llchss/kC9C)[10.1016/s0264-410x(02)00806-x](http://dx.doi.org/10.1016/s0264-410x(02)00806-x)[.](http://paperpile.com/b/llchss/kC9C)
94. [Ferrari Castilla AM, Pirez G MC, Martínez A A, Algorta R G, Chamorro F V, Guala B MJ, et al. Etiology of community acquired pneumonia in inpatients children. Uruguay 1998-2004. Rev Chilena Infectol 2007;24:40–7. https://doi.org/](http://paperpile.com/b/llchss/vao6)[10.4067/s0716-10182007000100006](http://dx.doi.org/10.4067/s0716-10182007000100006)[.](http://paperpile.com/b/llchss/vao6)
95. [Hortal M, Camou T, Palacio R, Dibarboure H, García A. Ten-year review of invasive pneumococcal diseases in children and adults from Uruguay: clinical spectrum, serotypes, and antimicrobial resistance. Int J Infect Dis 2000;4:91–5. https://doi.org/](http://paperpile.com/b/llchss/de82)[10.1016/s1201-9712(00)90100-0](http://dx.doi.org/10.1016/s1201-9712(00)90100-0)[.](http://paperpile.com/b/llchss/de82)
96. [Hortal M, Estevan M, Iraola I, De Mucio B. A population-based assessment of the disease burden of consolidated pneumonia in hospitalized children under five years of age. Int J Infect Dis 2007;11:273–7. https://doi.org/](http://paperpile.com/b/llchss/R6kI)[10.1016/j.ijid.2006.05.006](http://dx.doi.org/10.1016/j.ijid.2006.05.006)[.](http://paperpile.com/b/llchss/R6kI)
97. [Hortal M, Sehabiague G, Camou T, Iraola I, Estevan M, Pujadas M. Pneumococcal pneumonia in hospitalized Uruguayan children and potential prevention with different vaccine formulations. J Pediatr 2008;152:850–3. https://doi.org/](http://paperpile.com/b/llchss/2rOY)[10.1016/j.jpeds.2007.11.008](http://dx.doi.org/10.1016/j.jpeds.2007.11.008)[.](http://paperpile.com/b/llchss/2rOY)
98. [Machado K, López A, Pacheco H, Algorta G, Pírez C. Características del empiema paraneumónico luego del inicio de la vacunación antineumocócica: Centro Hospitalario Pereira Rossell, año 2010. Arch Pediatr Urug 2014;85:212–2219.](http://paperpile.com/b/llchss/YKnM)
99. [Machado K, Badía F, Assandri E, Gutiérrez C, Motta I, Varela A, et al. Neumonía necrotizante en niños: 10 años de experiencia en un hospital pediátrico de referencia. Arch Pediatr Urug 2020;91:294–302. https://doi.org/](http://paperpile.com/b/llchss/6ezx)[10.31134/ap.91.5.4](http://dx.doi.org/10.31134/ap.91.5.4)[.](http://paperpile.com/b/llchss/6ezx)
100. [Pírez MC, Martínez O, Ferrari AM, Nairac A, Montano A, Rubio I, et al. Standard case management of pneumonia in hospitalized children in Uruguay, 1997 to 1998. Pediatr Infect Dis J 2001;20:283–9. https://doi.org/](http://paperpile.com/b/llchss/5HNu)[10.1097/00006454-200103000-00013](http://dx.doi.org/10.1097/00006454-200103000-00013)[.](http://paperpile.com/b/llchss/5HNu)
101. [Pírez MC, Algorta G, Chamorro F, Romero C, Varela A, Cedres A, et al. Changes in hospitalizations for pneumonia after universal vaccination with pneumococcal conjugate vaccines 7/13 valent and haemophilus influenzae type b conjugate vaccine in a Pediatric Referral Hospital in Uruguay. Pediatr Infect Dis J 2014;33:753–9. https://doi.org/](http://paperpile.com/b/llchss/fqD2)[10.1097/INF.0000000000000294](http://dx.doi.org/10.1097/INF.0000000000000294)[.](http://paperpile.com/b/llchss/fqD2)
102. [Pírez MC, Mota MI, Giachetto G, Sánchez Varela M, Galazka J, Gutierrez S, et al. Pneumococcal Meningitis Before and After Universal Vaccination With Pneumococcal Conjugate Vaccines 7/13, Impact on Pediatric Hospitalization in Public and Nonpublic Institutions, in Uruguay. Pediatr Infect Dis J 2017;36:1000–1. https://doi.org/](http://paperpile.com/b/llchss/i2Jt)[10.1097/INF.0000000000001671](http://dx.doi.org/10.1097/INF.0000000000001671)[.](http://paperpile.com/b/llchss/i2Jt)
103. [Pírez García MC, Giachetto Larraz G, Romero Rostagno C, Zabala Chain C, Algorta Rusiñol G, Montano Lotito A, et al. Neumonía neumocócica invasiva en niños de 0 a 24 meses: ¿influye la resistencia bacteriana en la evolución? Anales de Pediatría 2008;69:205–9. https://doi.org/](http://paperpile.com/b/llchss/0RDp)[10.1157/13125812](http://dx.doi.org/10.1157/13125812)[.](http://paperpile.com/b/llchss/0RDp)
